# Supplementary material for: Oncolytic viruses expressing MATEs facilitate target-independent T-cell activation in tumors
Source: EMBO Mol Med. 2025 Jan 9;17(2):265–300. doi: 10.1038/s44321-024-00187-y (PMC11821991; doi:10.1038/s44321-024-00187-y)
Supplement: Supplementary file 1 — Appendix [file 44321_2024_187_MOESM1_ESM.pdf]

Appendix

Content	Page
Appendix Figure S1	1

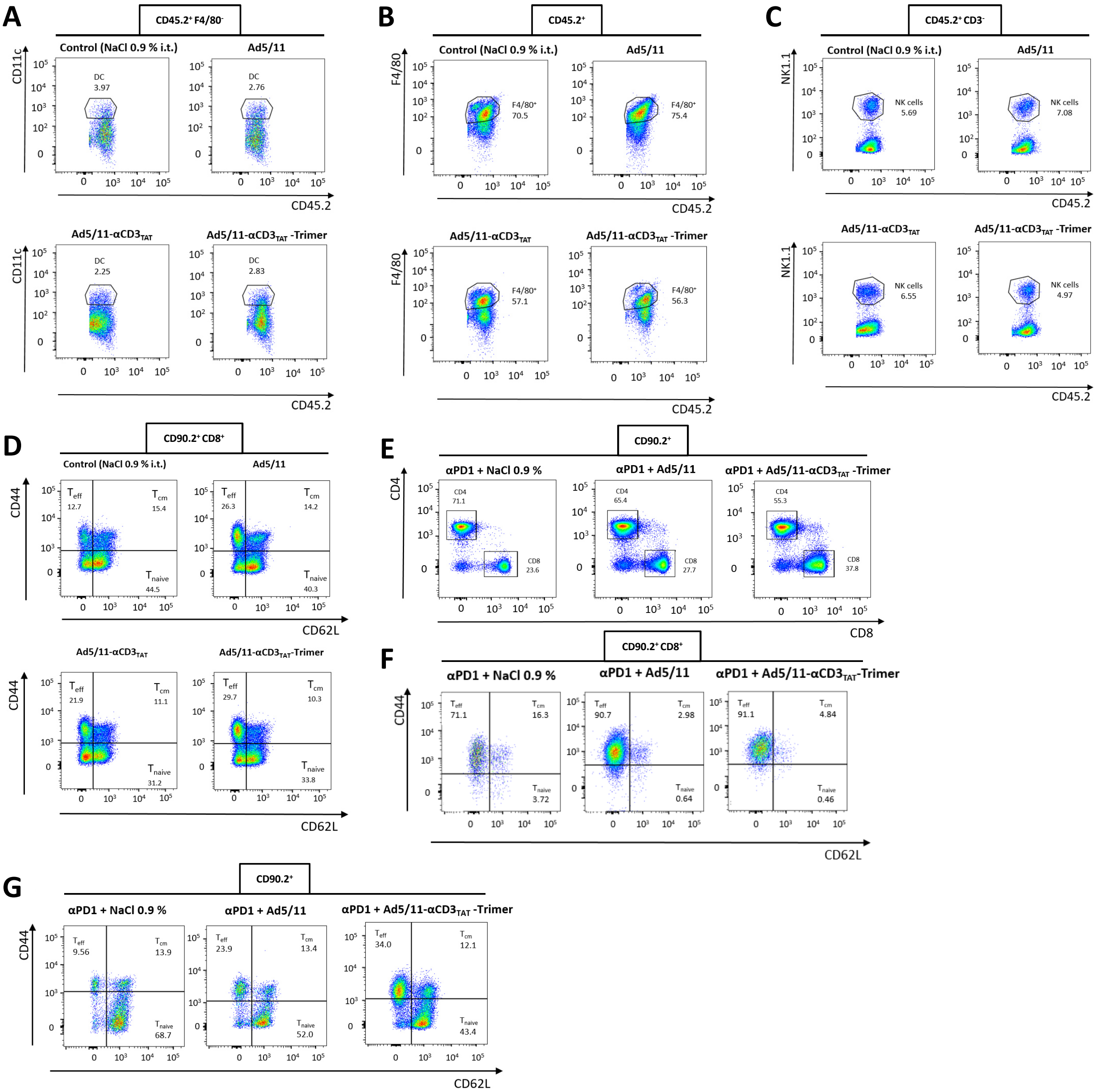

Appendix Figure S1: (A-C) FACS analysis of intratumoral dendritic cells, macrophages and NK cells seven days after virotherapy according the analysis shown in Fig EV4. Frequencies state the proportion of total Leukocytes. (D) FACS analysis of splenocytes for T-cell subsets within the CD90.2+ CD8+ lymphocyte population seven days after treatment according to the analysis shown in Fig EV4 F. (E) Mice were treated with virotherapy followed by two doses of αPD1 antibodies. Spleens were analyzed via FACS for CD4 and CD8 T cells seven days after therapy start according to the analysis shown in Fig 7 E. (F) Mice were treated with virotherapy followed by two doses of αPD1 antibodies and sacrificed seven days after therapy start. FACS analysis of intratumoral T-cell subsets within the CD90.2+ CD8+ lymphocyte population is shown according to the analysis shown in Fig EV5 C. (G) Mice were treated with virotherapy followed by two doses of αPD1 antibodies and analyzed on day seven after therapy start. FACS analysis of spleens for T-cell subsets within the population of CD90.2+ lymphocytes according to the analysis shown in Fig EV 5 F. All images show a representative plot for each group. Pre-gate is specified above the plots.
