# Supplementary figures and images for: Oncolytic viruses expressing MATEs facilitate target-independent T-cell activation in tumors
Source: EMBO Mol Med. 2025 Jan 9;17(2):265–300. doi: 10.1038/s44321-024-00187-y (PMC11821991; doi:10.1038/s44321-024-00187-y)

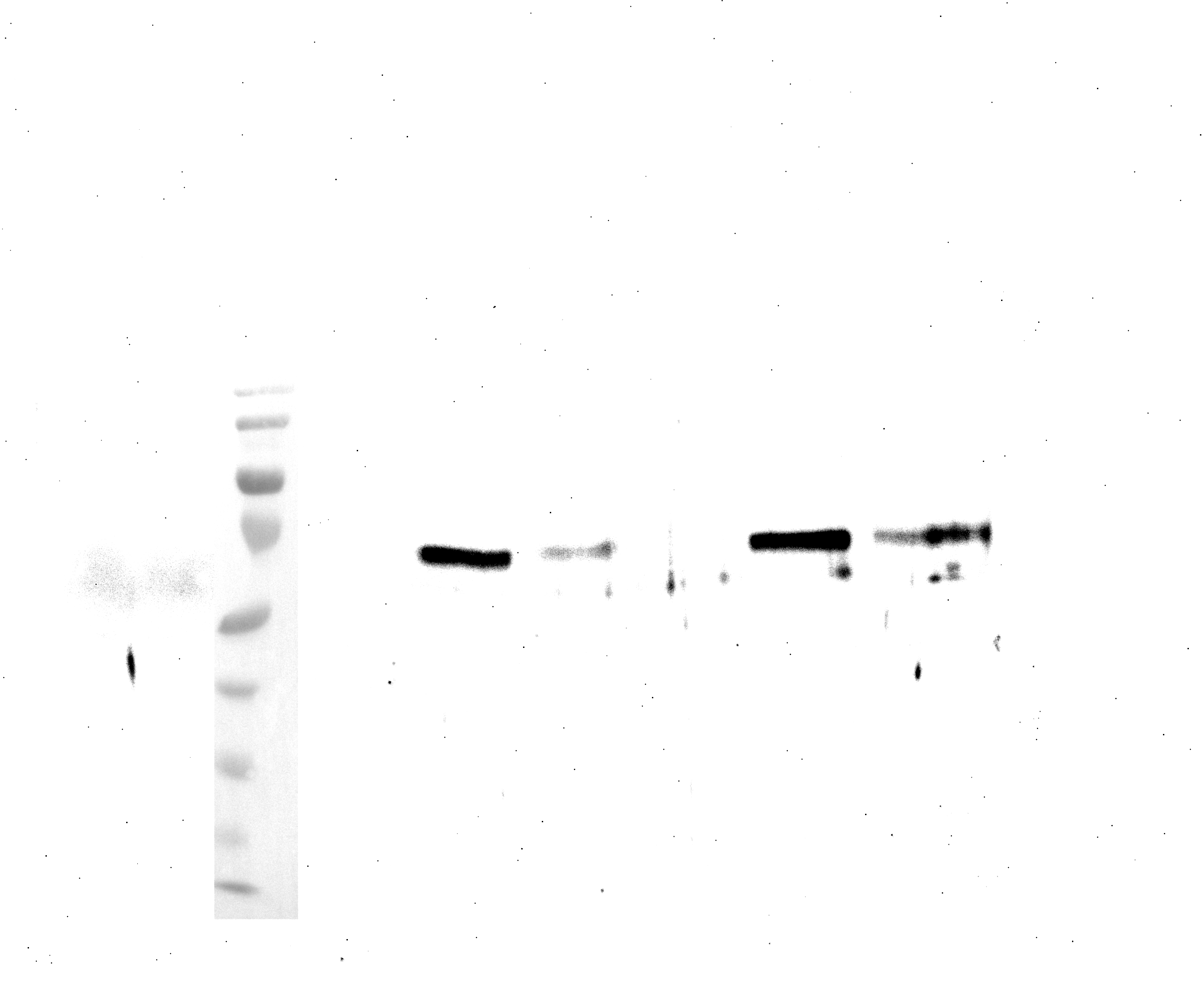

Supplement: Supplementary file 5 — Source data Fig. 3 [file 44321_2024_187_MOESM5_ESM.zip › Figure 3/3B/5.30 min_WB_c-myc.tif]

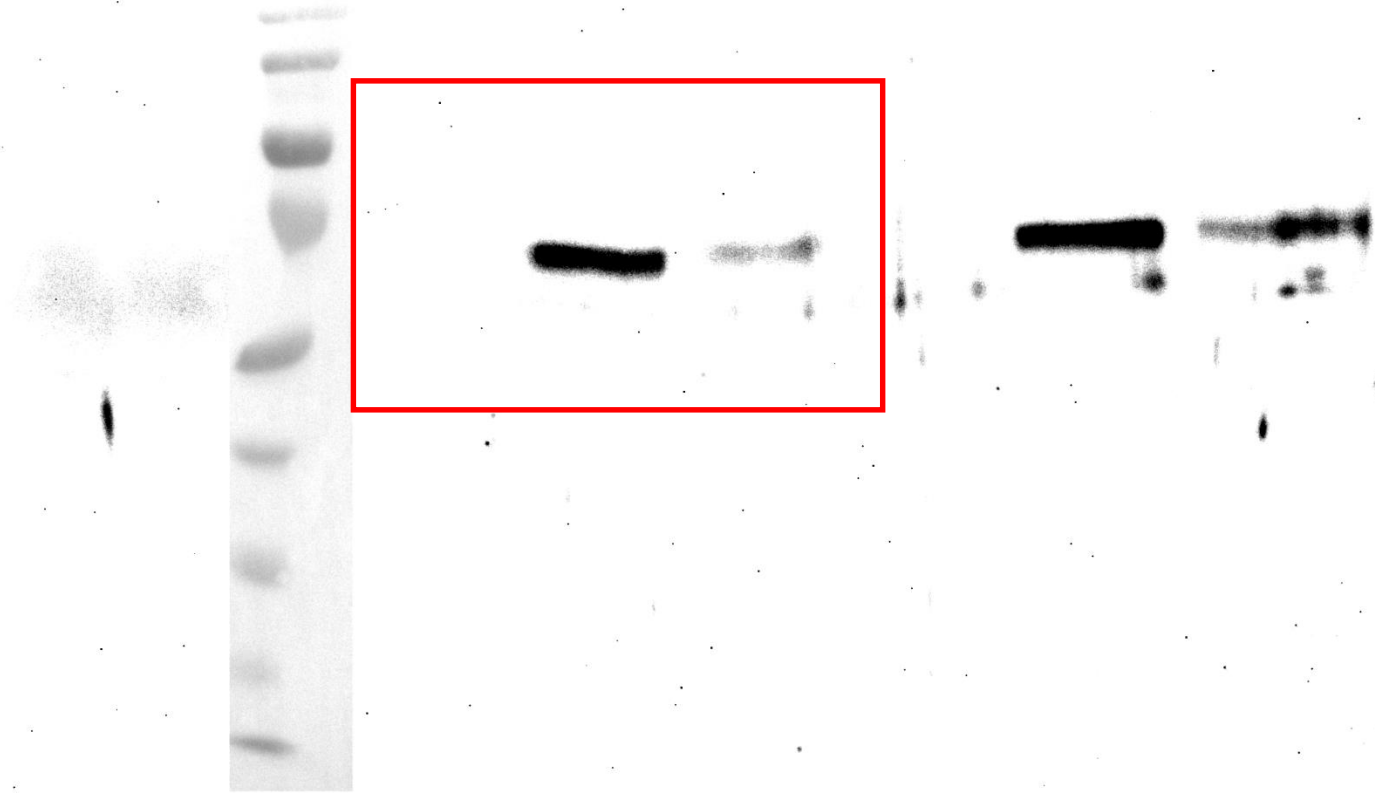

Supplement: Supplementary file 5 — Source data Fig. 3 [file 44321_2024_187_MOESM5_ESM.zip › Figure 3/3B/field of view.pdf]

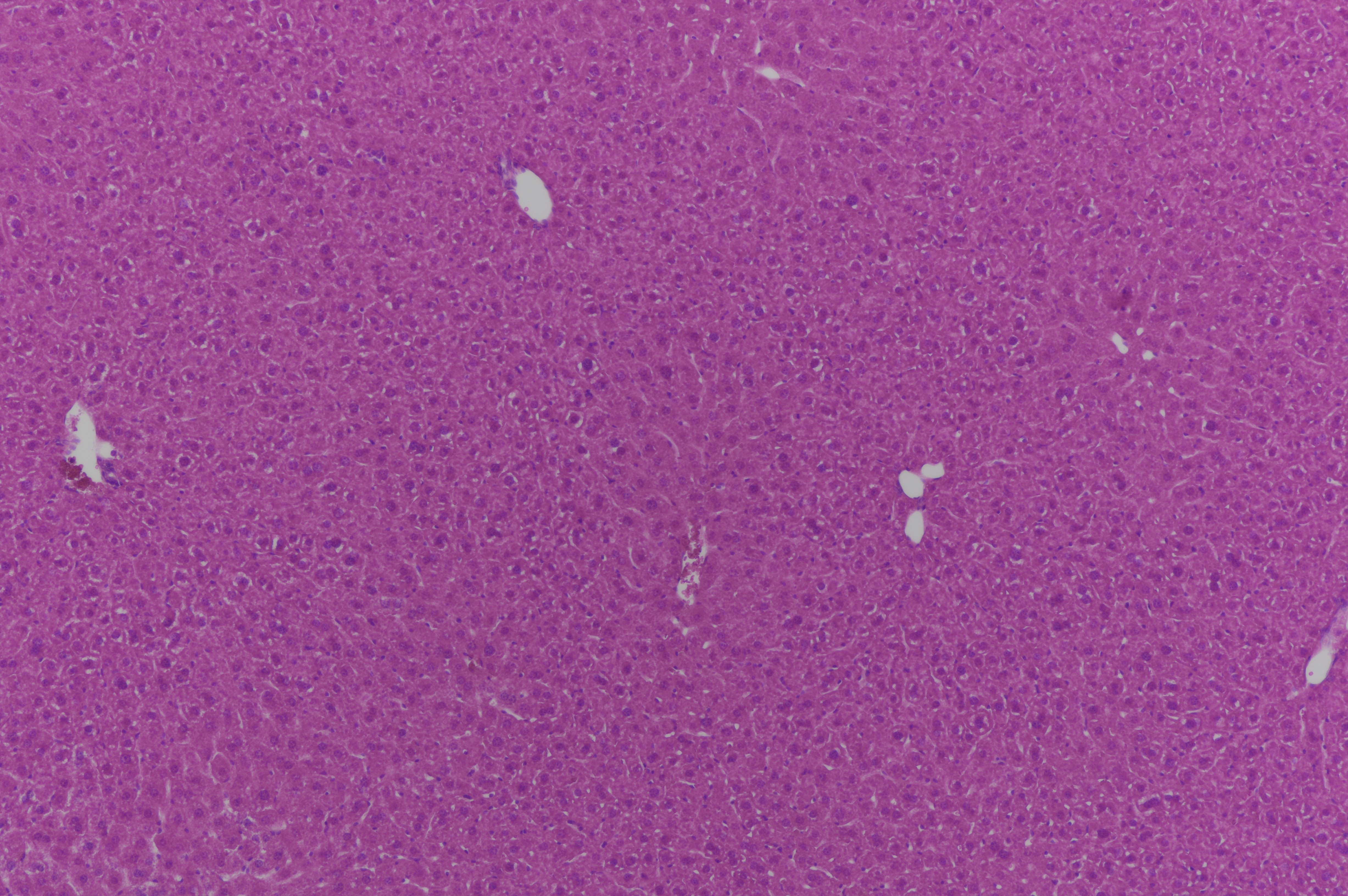

Supplement: Supplementary file 6 — Source data Fig. 4 [file 44321_2024_187_MOESM6_ESM.zip › Figure 4/4D/Ad5_11.jpg]

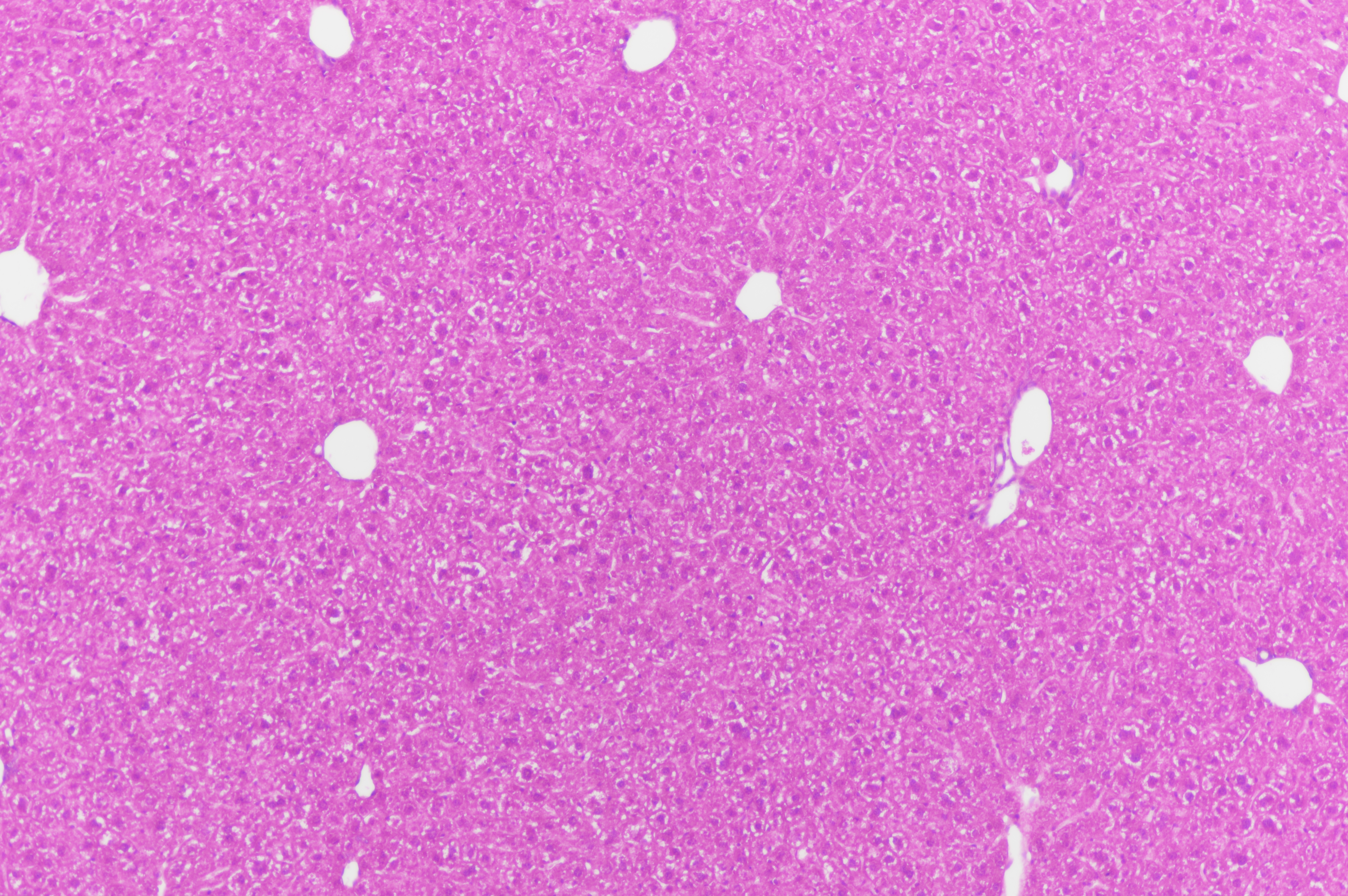

Supplement: Supplementary file 6 — Source data Fig. 4 [file 44321_2024_187_MOESM6_ESM.zip › Figure 4/4D/Ad5_11_CD3_TAT.jpg]

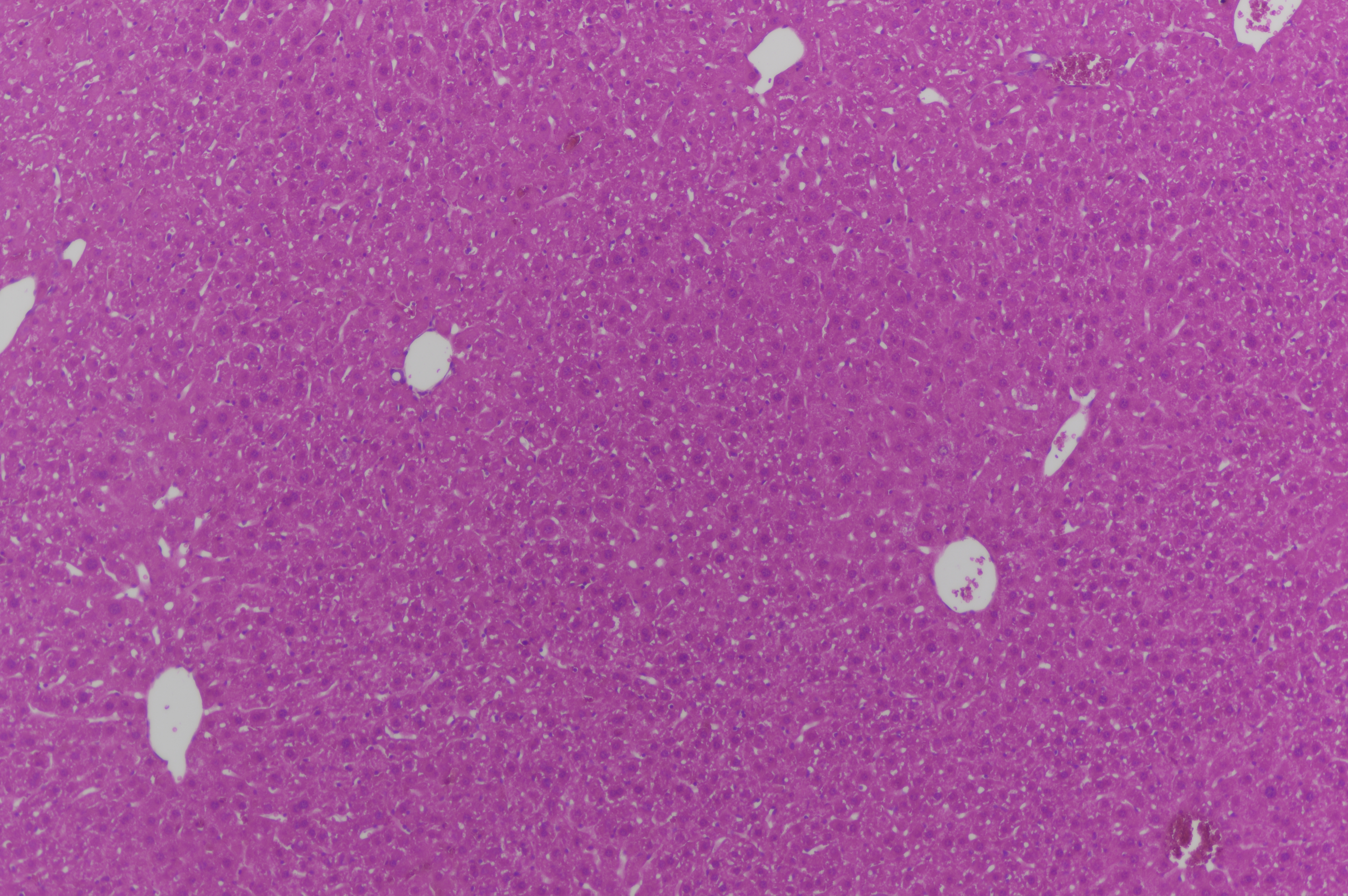

Supplement: Supplementary file 6 — Source data Fig. 4 [file 44321_2024_187_MOESM6_ESM.zip › Figure 4/4D/Ad5_11_CD3_TAT_Trimer.jpg]

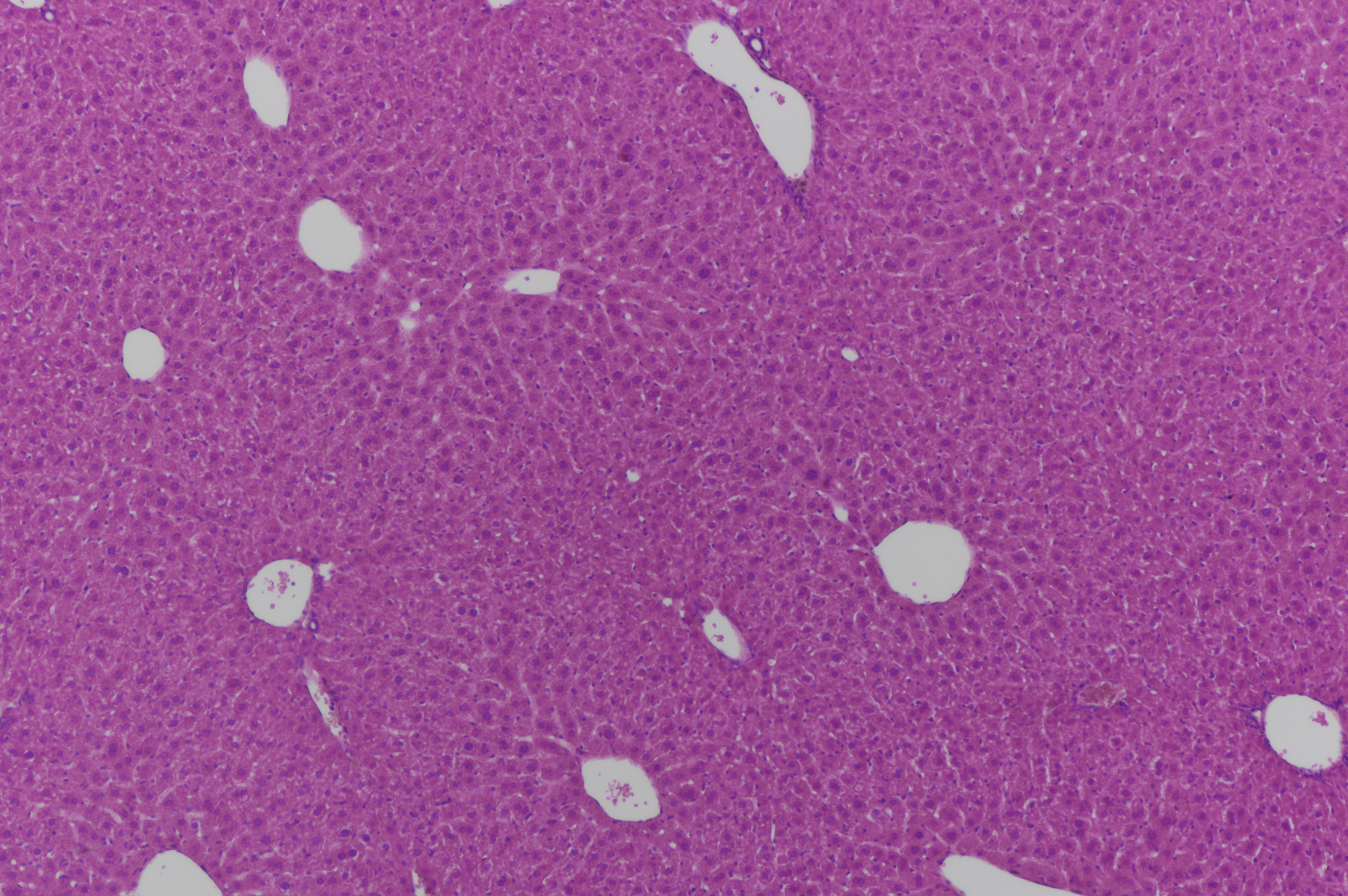

Supplement: Supplementary file 6 — Source data Fig. 4 [file 44321_2024_187_MOESM6_ESM.zip › Figure 4/4D/Mock.jpg]

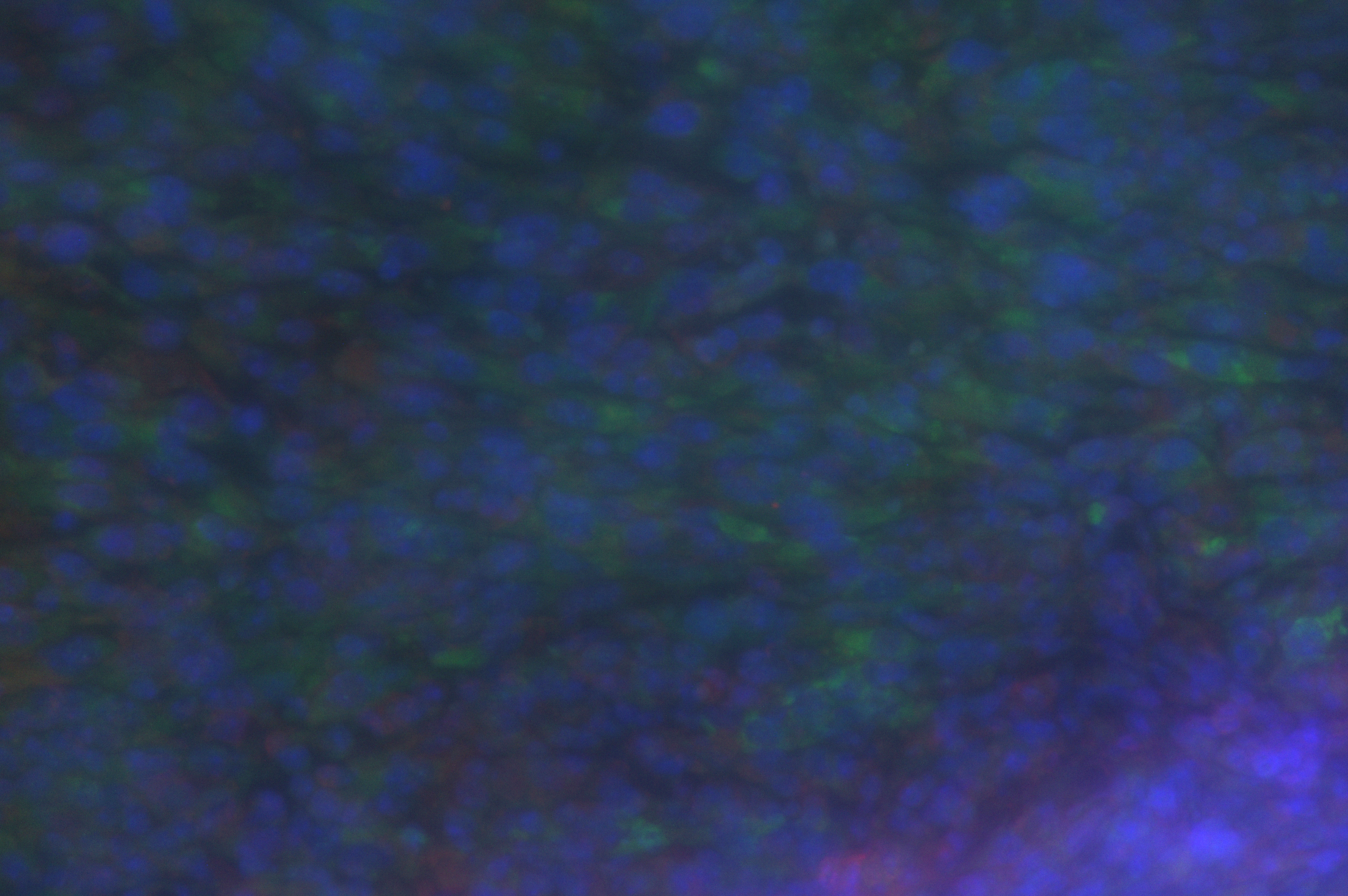

Supplement: Supplementary file 6 — Source data Fig. 4 [file 44321_2024_187_MOESM6_ESM.zip › Figure 4/4E/Ad5_11 40x.tif]

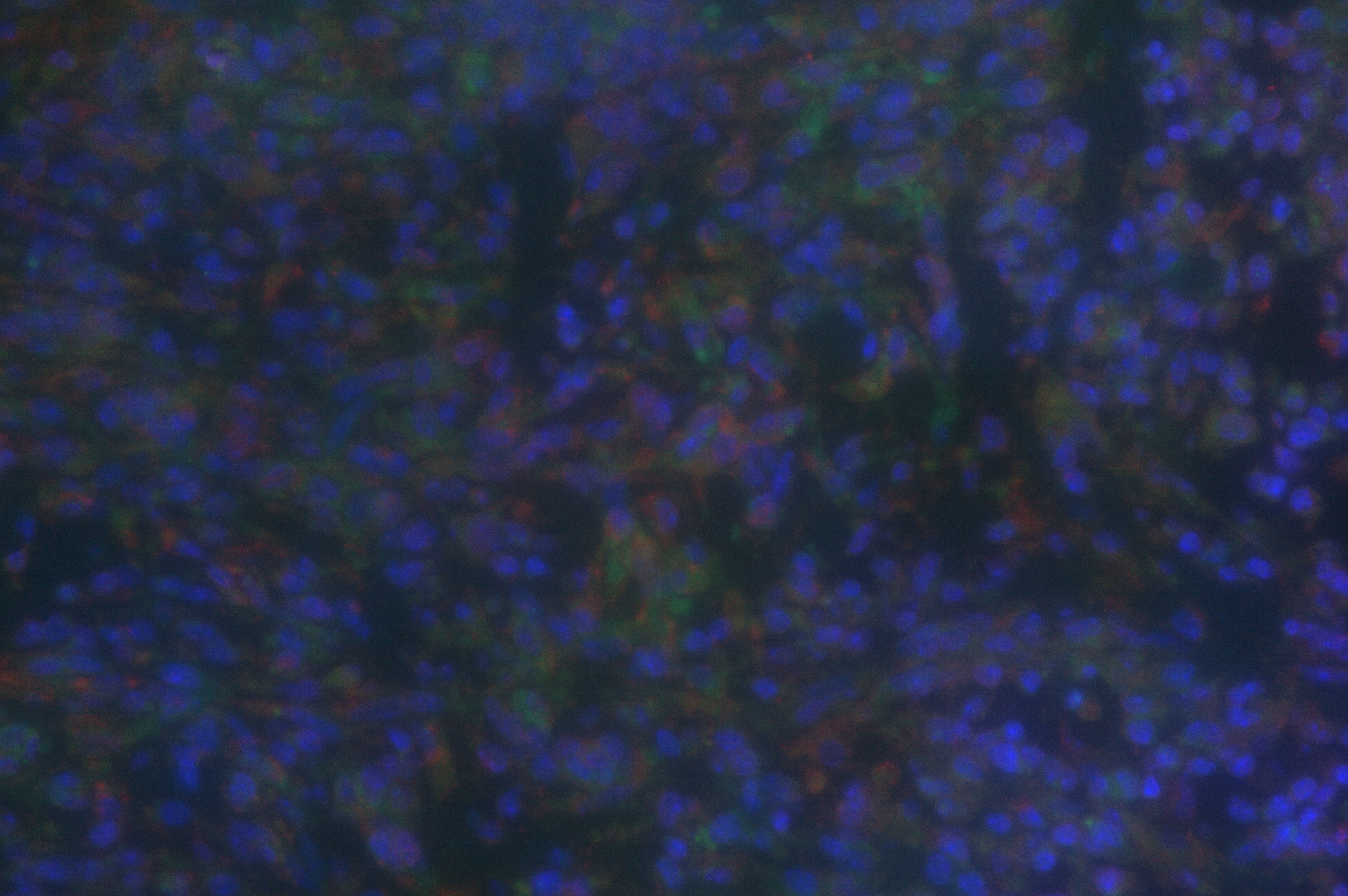

Supplement: Supplementary file 6 — Source data Fig. 4 [file 44321_2024_187_MOESM6_ESM.zip › Figure 4/4E/Ad5_11_CD3_TAT 40x.tif]

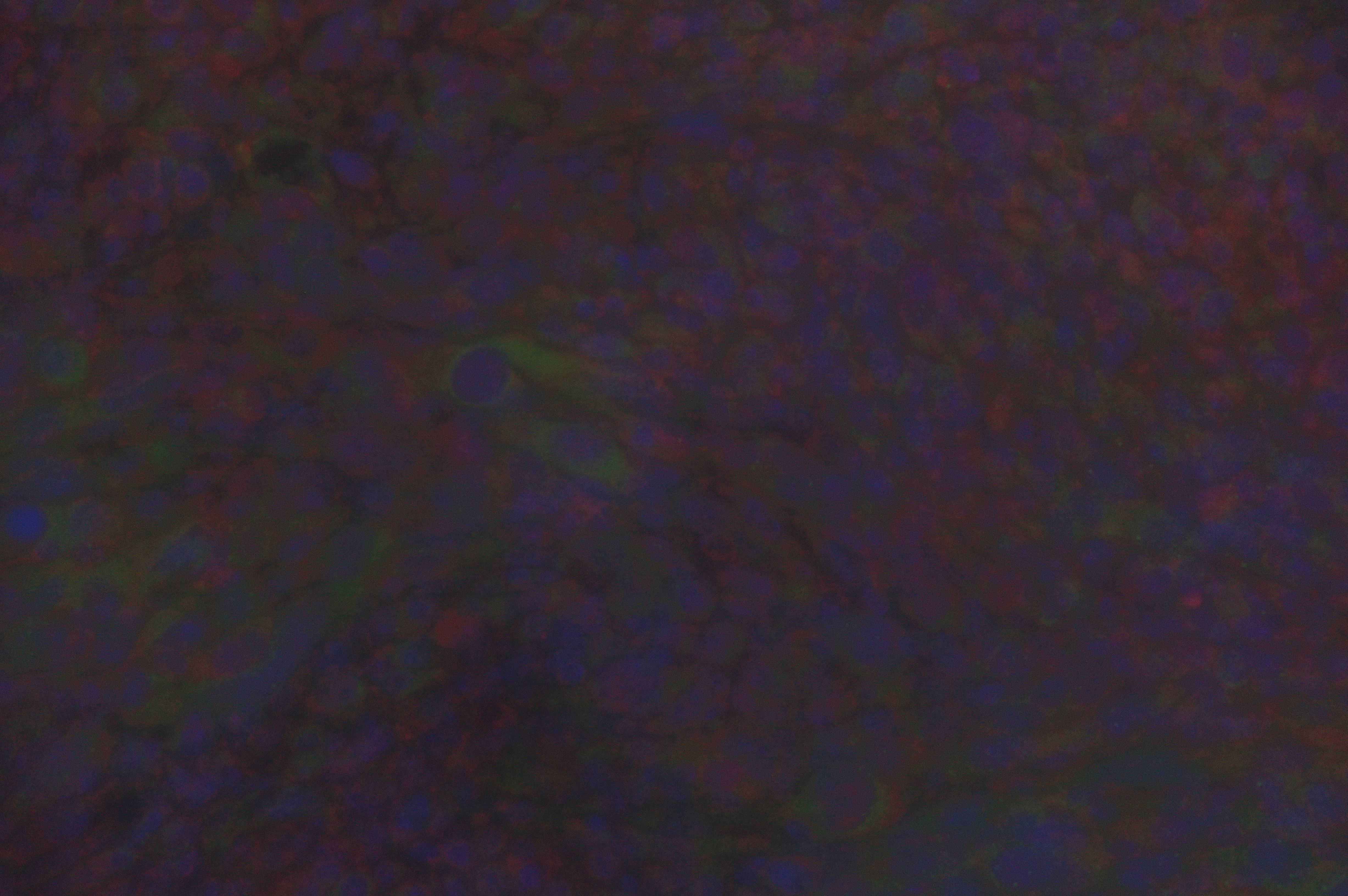

Supplement: Supplementary file 6 — Source data Fig. 4 [file 44321_2024_187_MOESM6_ESM.zip › Figure 4/4E/Ad5_11_CD3_TAT_Trimer 40x.tif]

mock

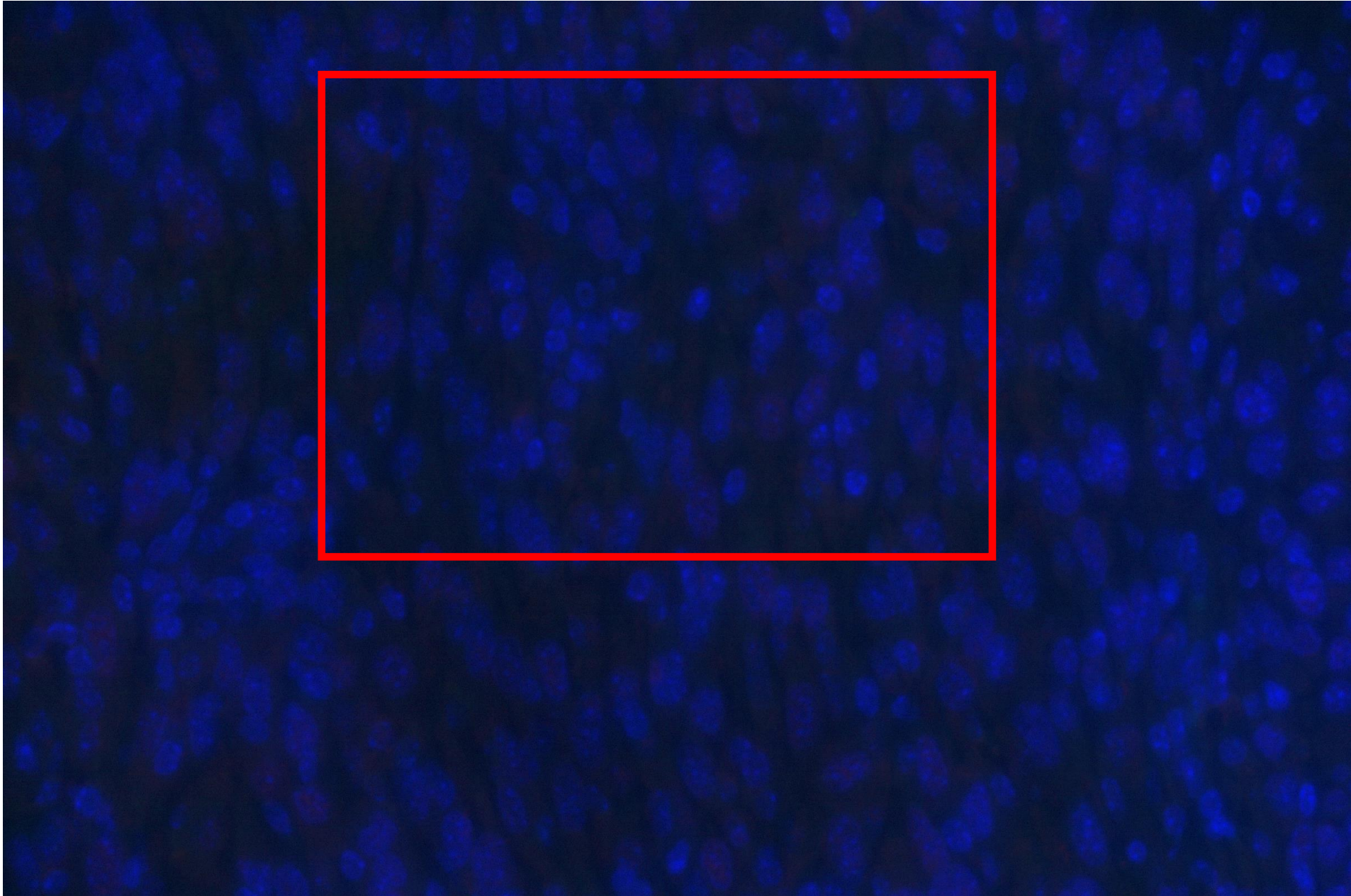

Ad5/11

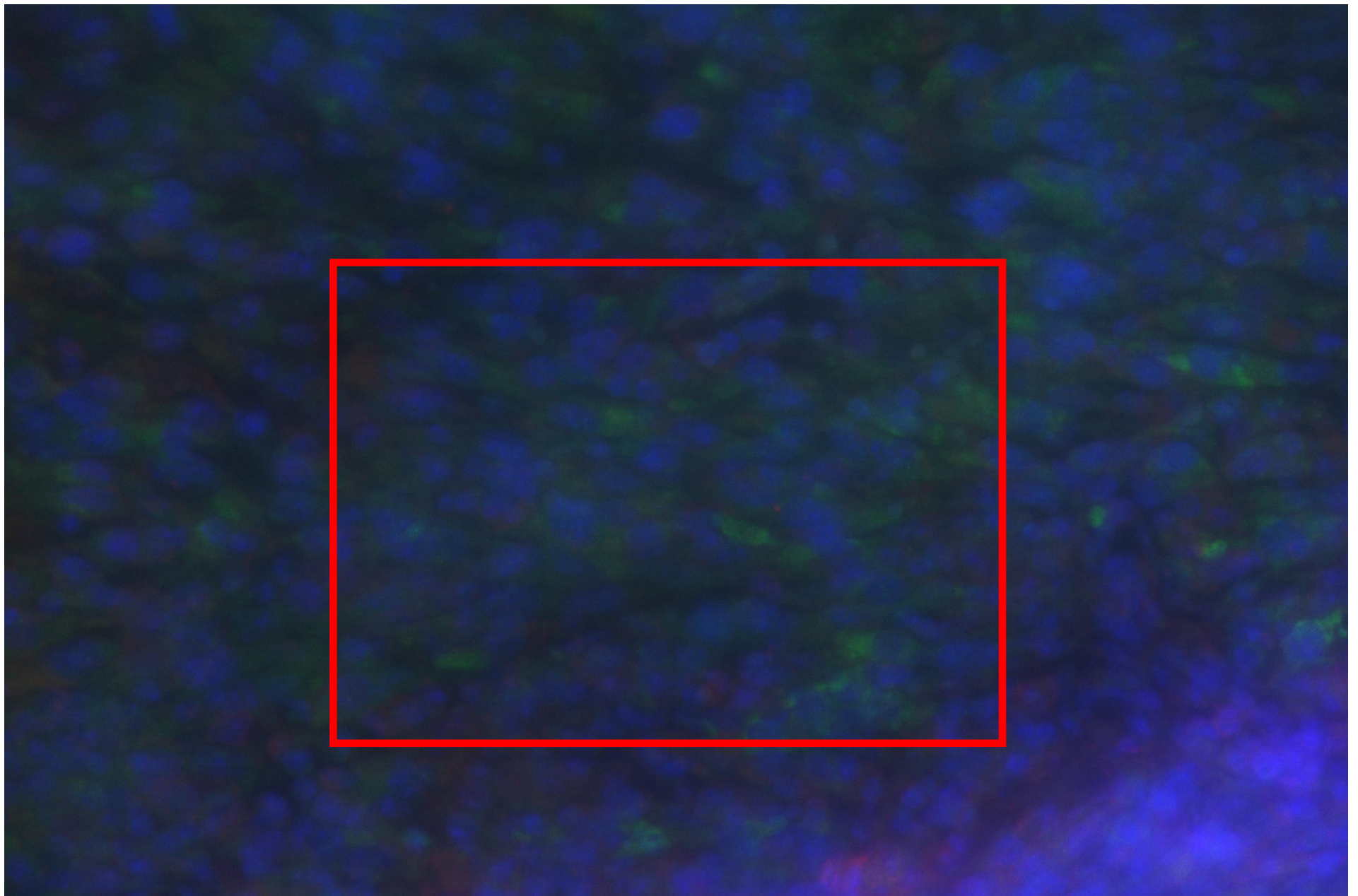

Ad5/11  
CD3 TAT

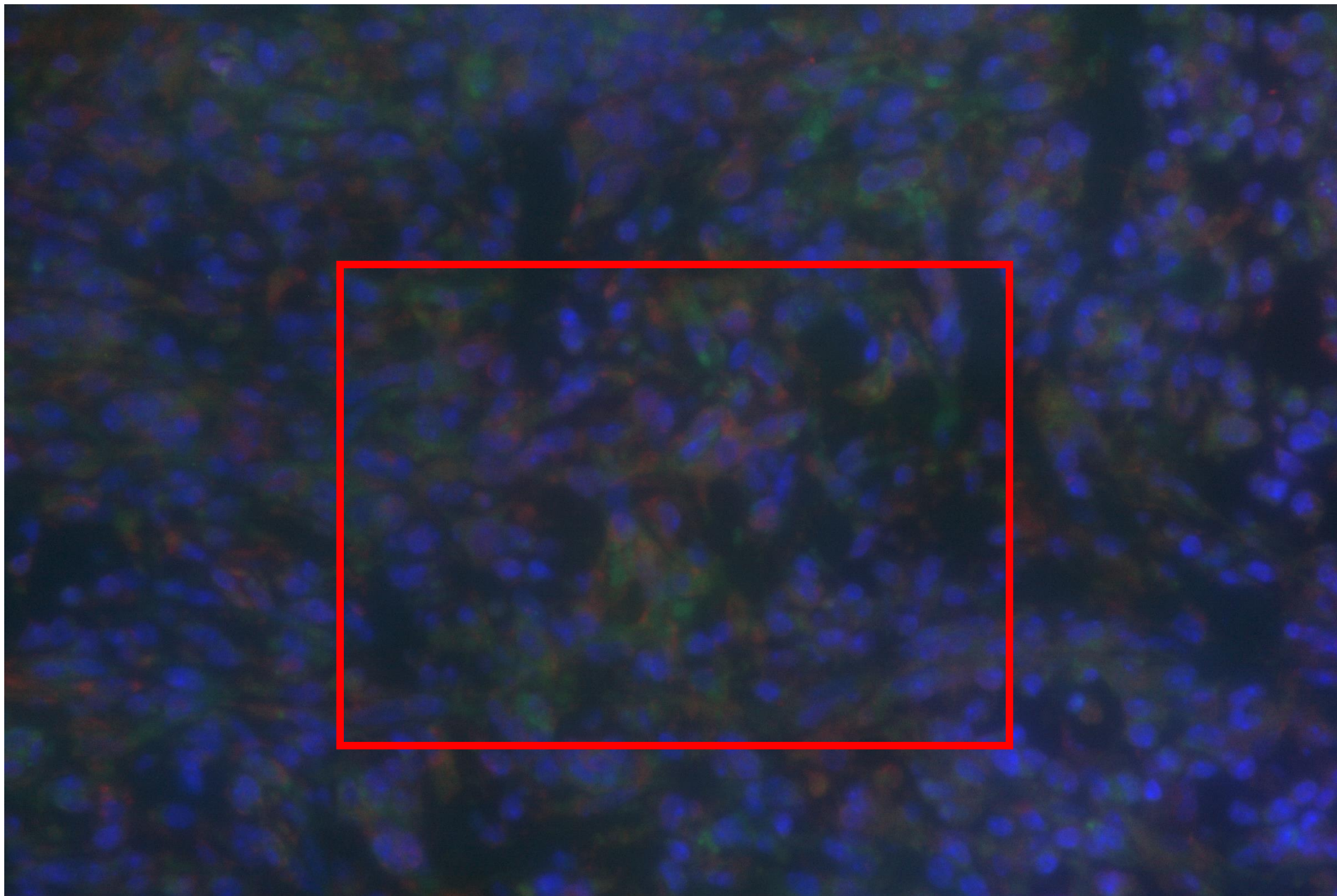

Ad5/11  
CD3 TAT  
Trimer

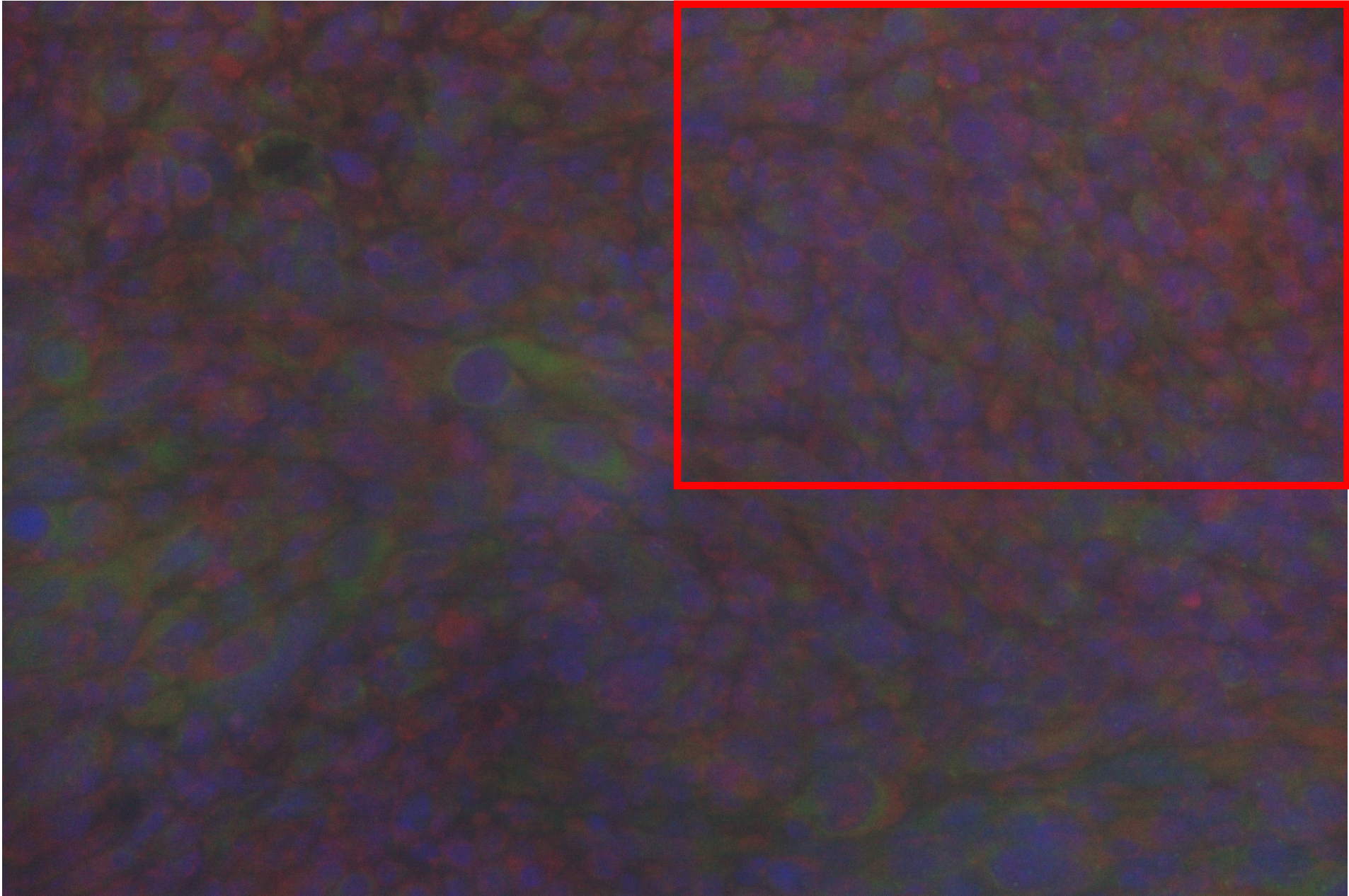

Supplement: Supplementary file 6 — Source data Fig. 4 [file 44321_2024_187_MOESM6_ESM.zip › Figure 4/4E/Fig4E_area of magnification.pdf]

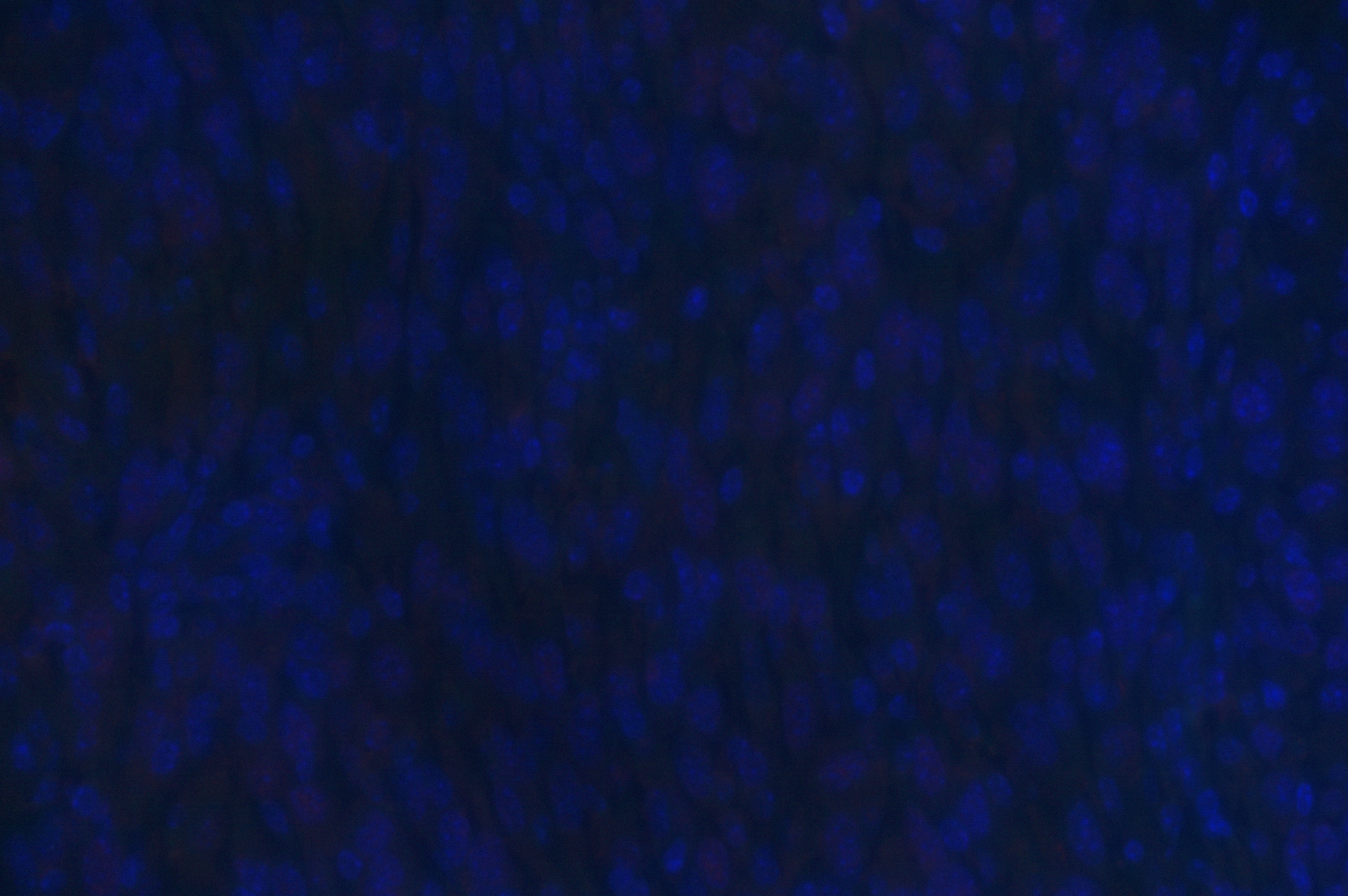

Supplement: Supplementary file 6 — Source data Fig. 4 [file 44321_2024_187_MOESM6_ESM.zip › Figure 4/4E/Mock 40x.tif]

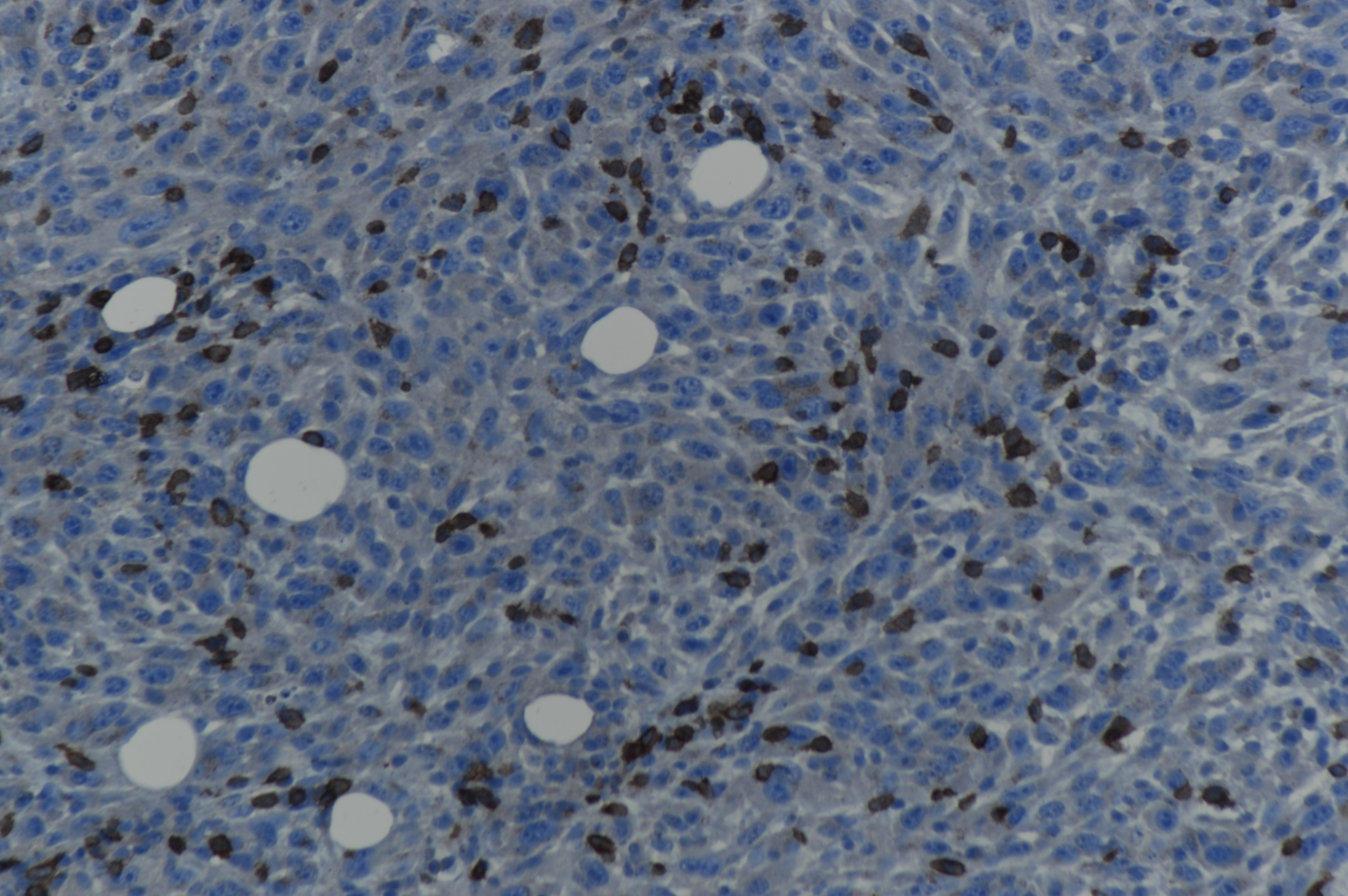

Supplement: Supplementary file 7 — Source data Fig. 5 [file 44321_2024_187_MOESM7_ESM.zip › Figure 5/5C/CD3 Ad5_11.jpg]

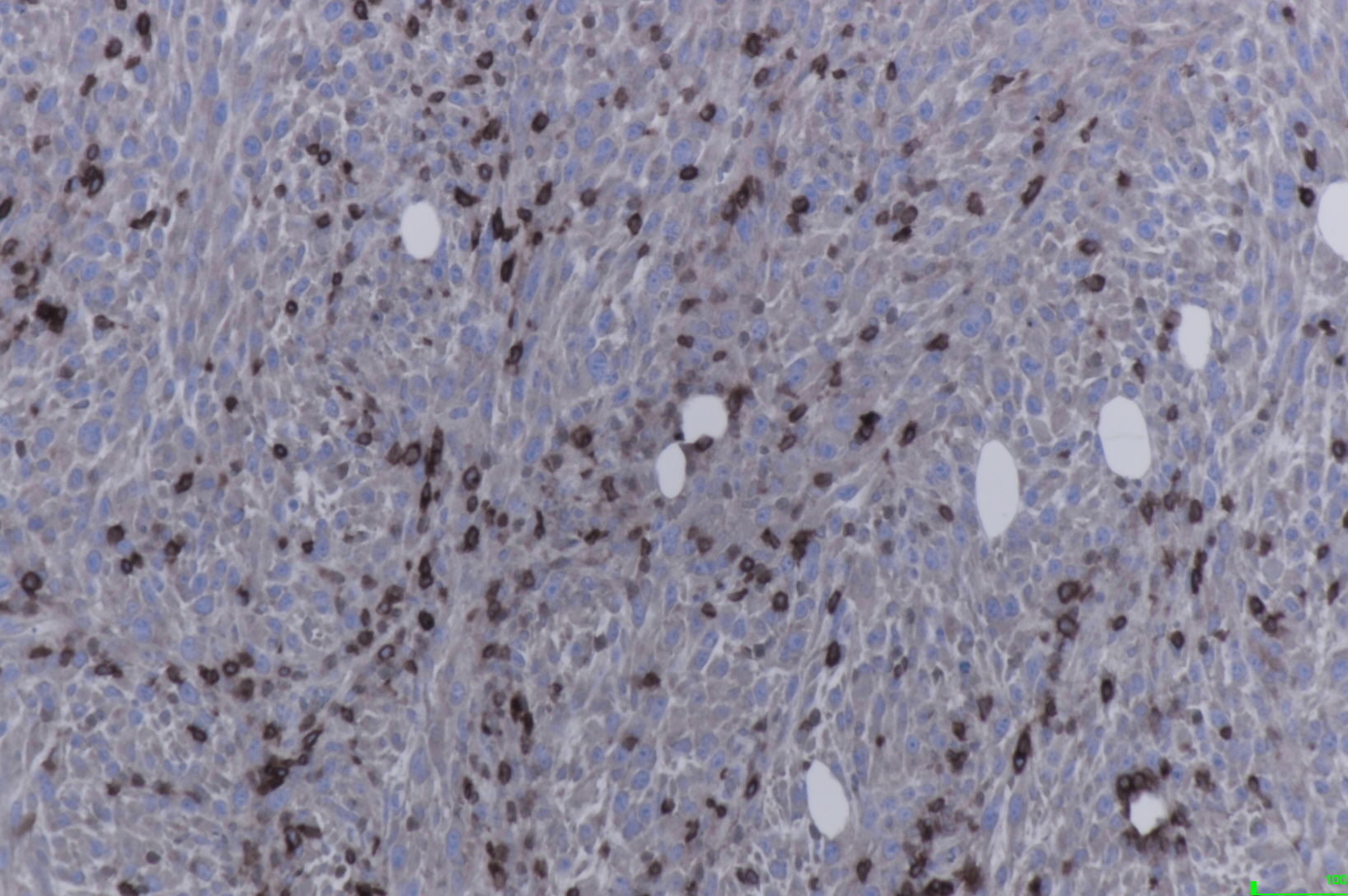

Supplement: Supplementary file 7 — Source data Fig. 5 [file 44321_2024_187_MOESM7_ESM.zip › Figure 5/5C/CD3 Ad5_11_CD3_TAT.jpg]

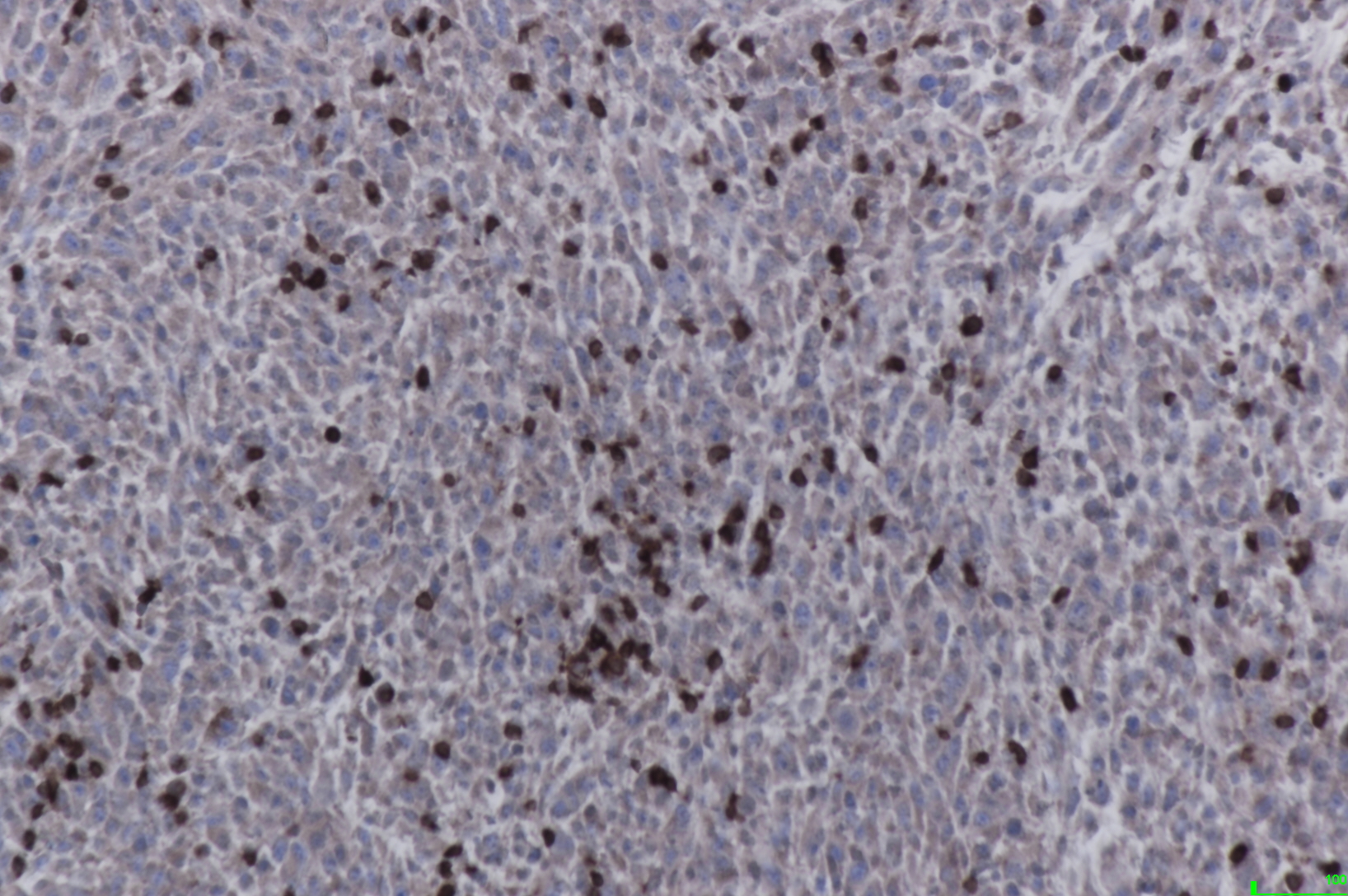

Supplement: Supplementary file 7 — Source data Fig. 5 [file 44321_2024_187_MOESM7_ESM.zip › Figure 5/5C/CD3 Ad5_11_CD3_TAT_Trimer.jpg]

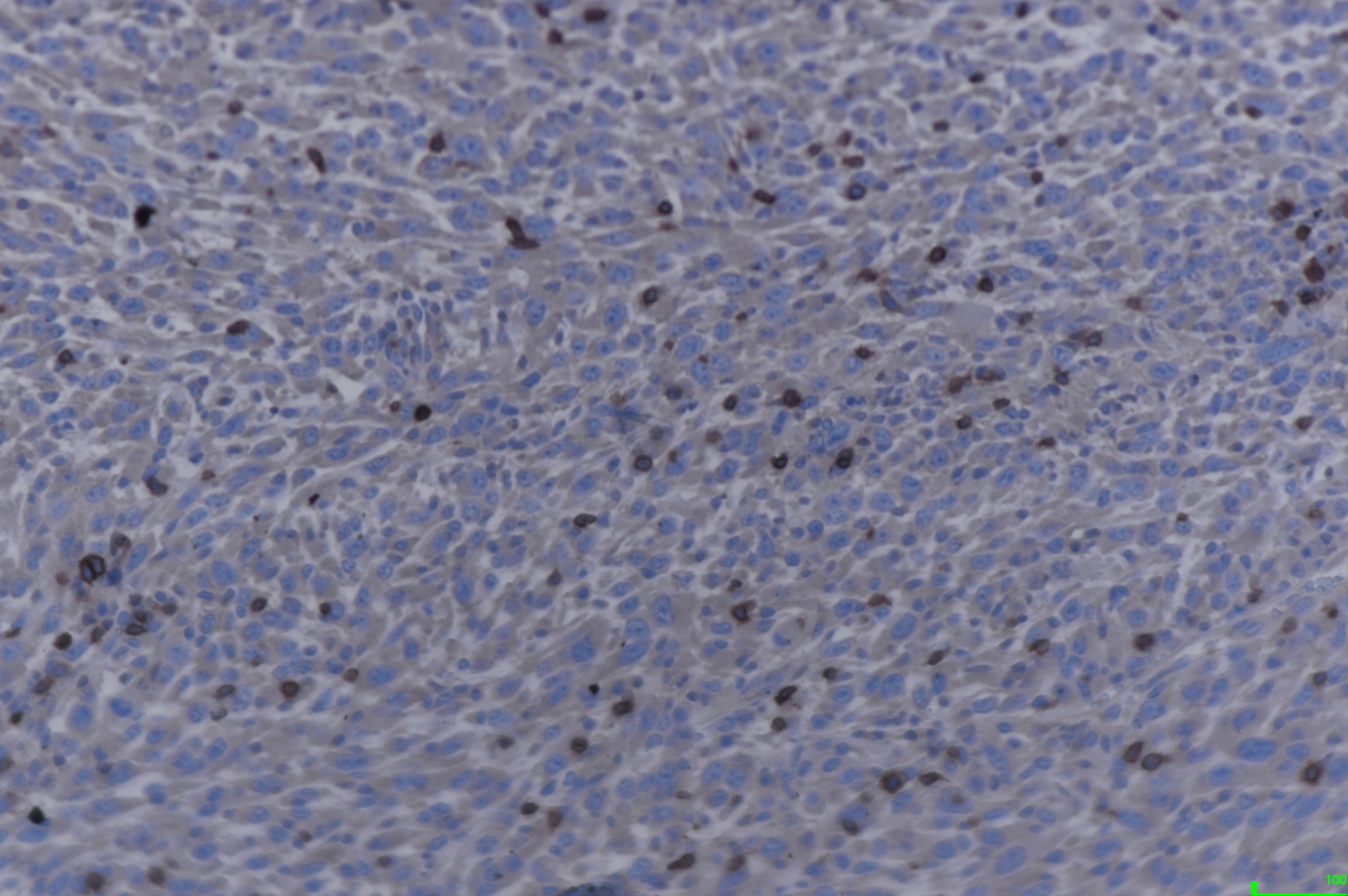

Supplement: Supplementary file 7 — Source data Fig. 5 [file 44321_2024_187_MOESM7_ESM.zip › Figure 5/5C/CD3 Control.jpg]

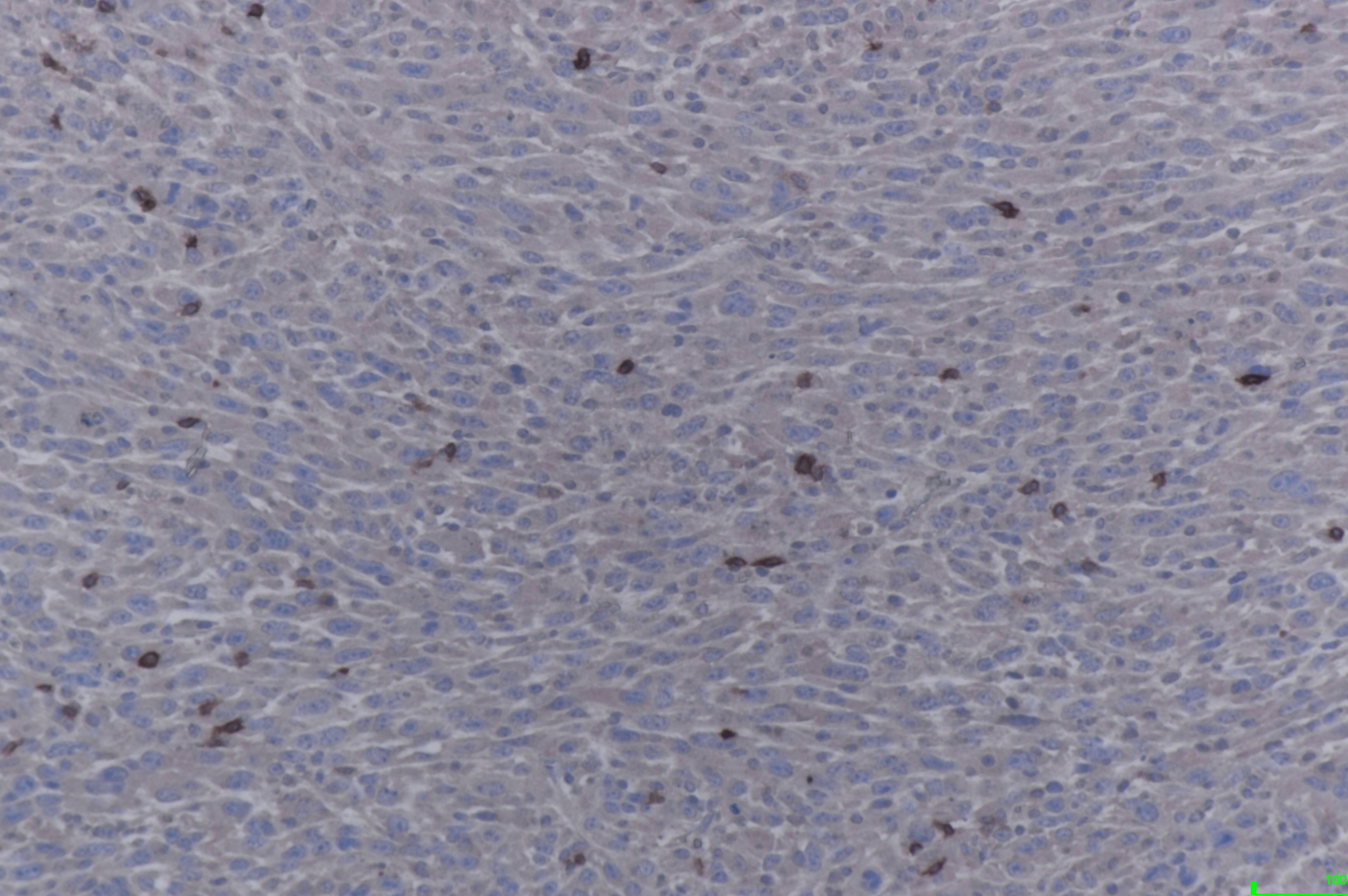

Supplement: Supplementary file 7 — Source data Fig. 5 [file 44321_2024_187_MOESM7_ESM.zip › Figure 5/5C/CD4 Ad5_11.jpg]

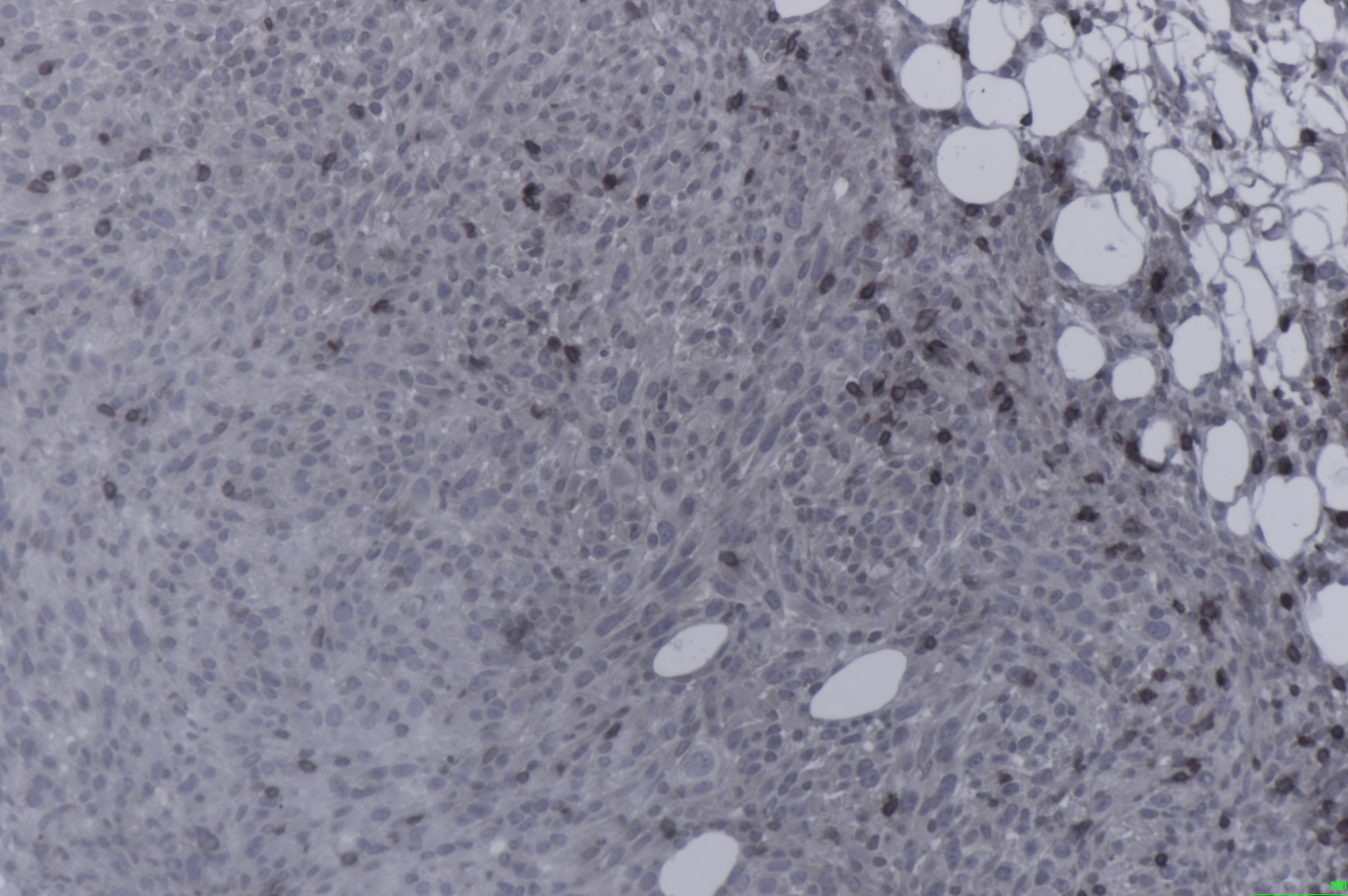

Supplement: Supplementary file 7 — Source data Fig. 5 [file 44321_2024_187_MOESM7_ESM.zip › Figure 5/5C/CD4 Ad5_11_CD3_TAT.jpg]

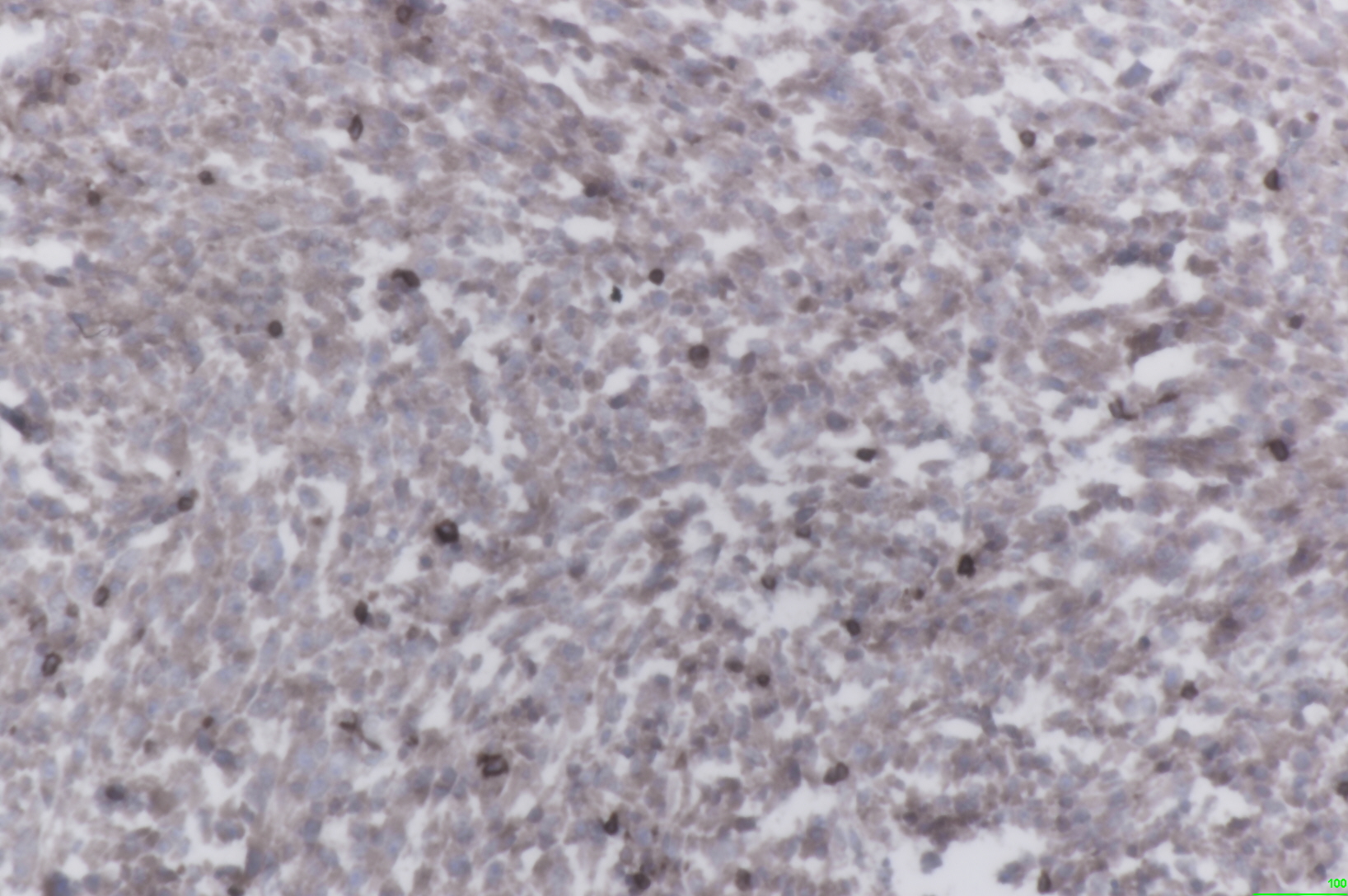

Supplement: Supplementary file 7 — Source data Fig. 5 [file 44321_2024_187_MOESM7_ESM.zip › Figure 5/5C/CD4 Ad5_11_CD3_TAT_Trimer.jpg]

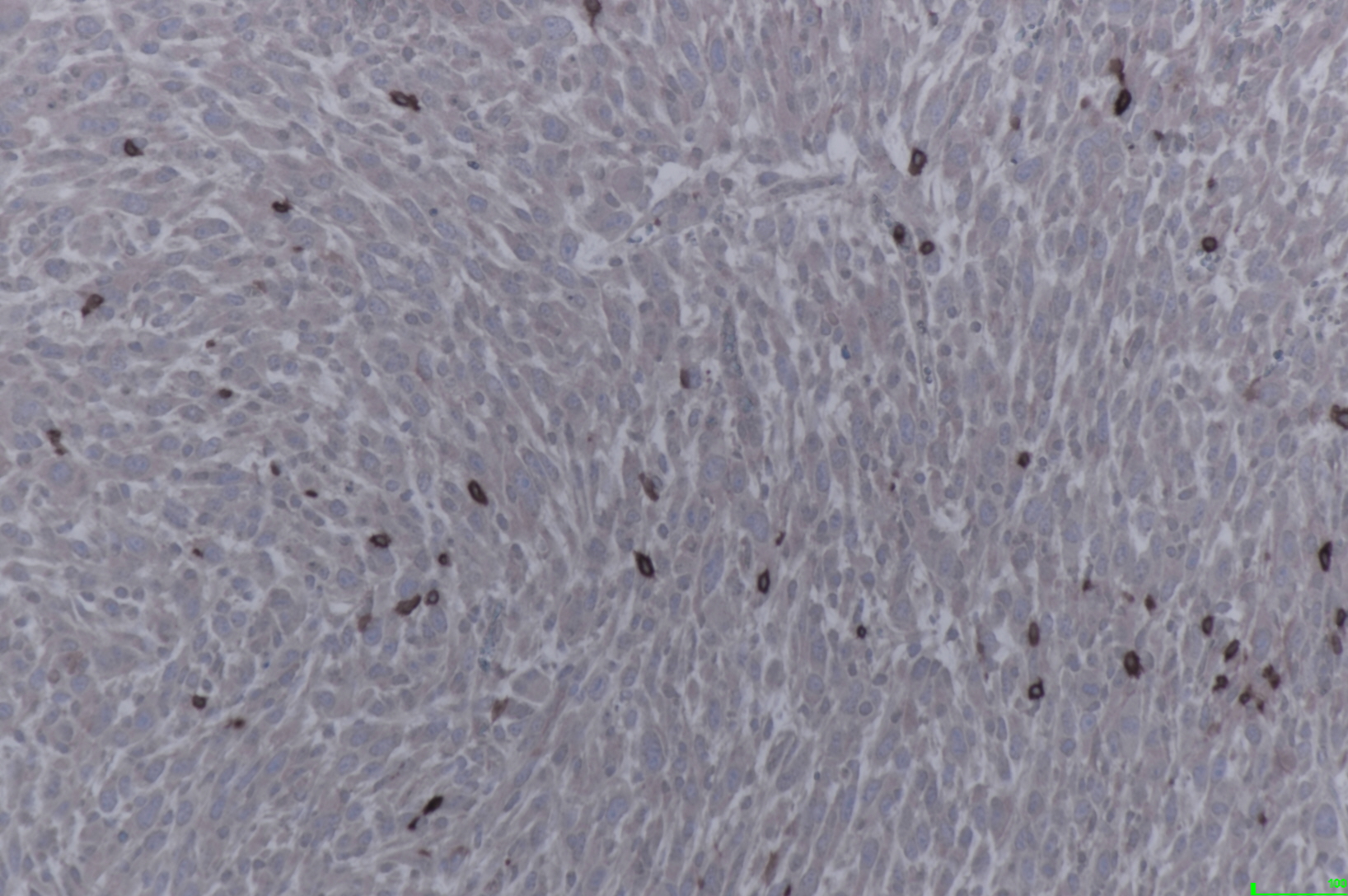

Supplement: Supplementary file 7 — Source data Fig. 5 [file 44321_2024_187_MOESM7_ESM.zip › Figure 5/5C/CD4 Control.jpg]

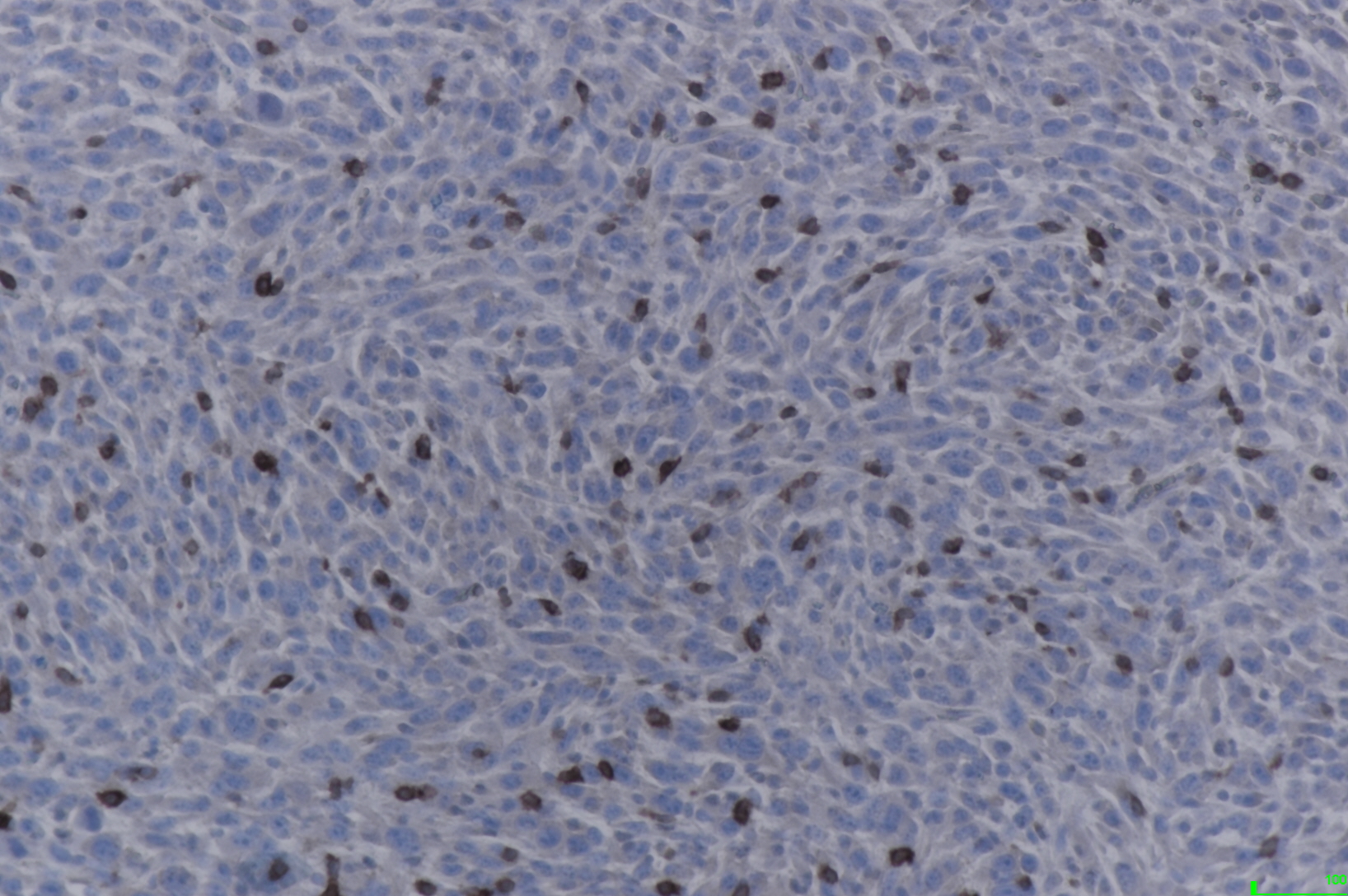

Supplement: Supplementary file 7 — Source data Fig. 5 [file 44321_2024_187_MOESM7_ESM.zip › Figure 5/5C/CD8 Ad5_11.jpg]

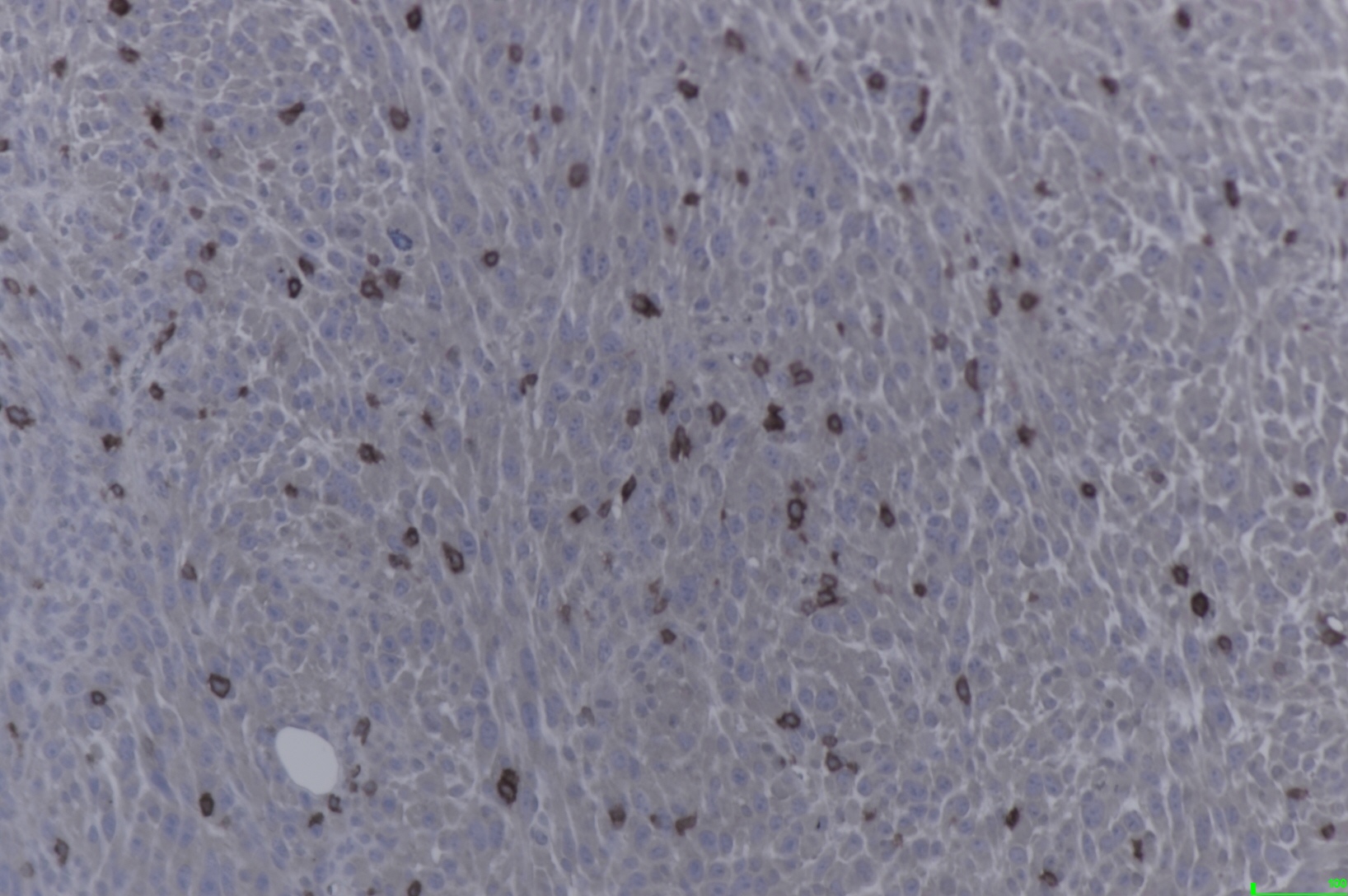

Supplement: Supplementary file 7 — Source data Fig. 5 [file 44321_2024_187_MOESM7_ESM.zip › Figure 5/5C/CD8 Ad5_11_CD3_TAT.jpg]

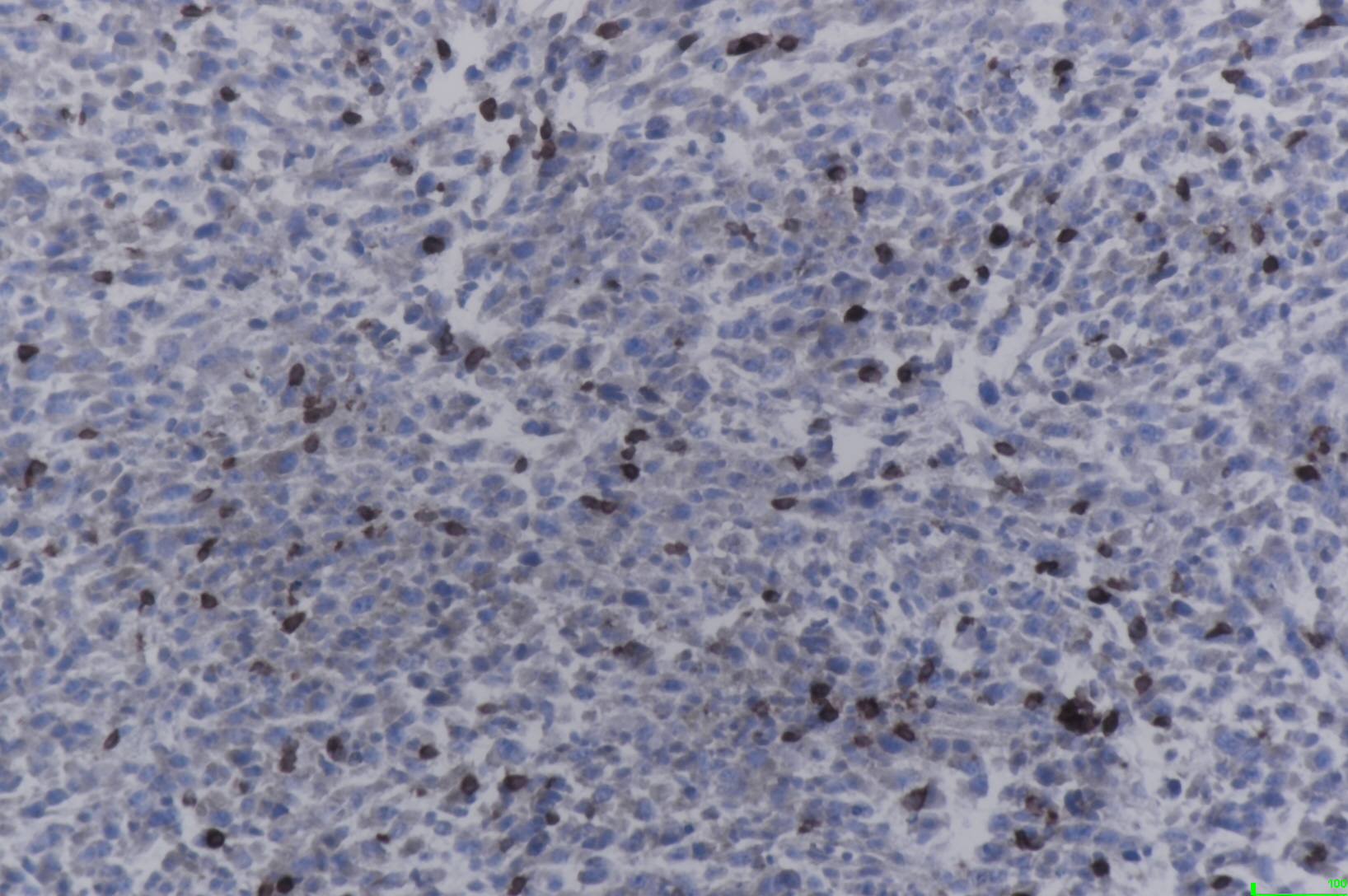

Supplement: Supplementary file 7 — Source data Fig. 5 [file 44321_2024_187_MOESM7_ESM.zip › Figure 5/5C/CD8 Ad5_11_CD3_TAT_Trimer.jpg]

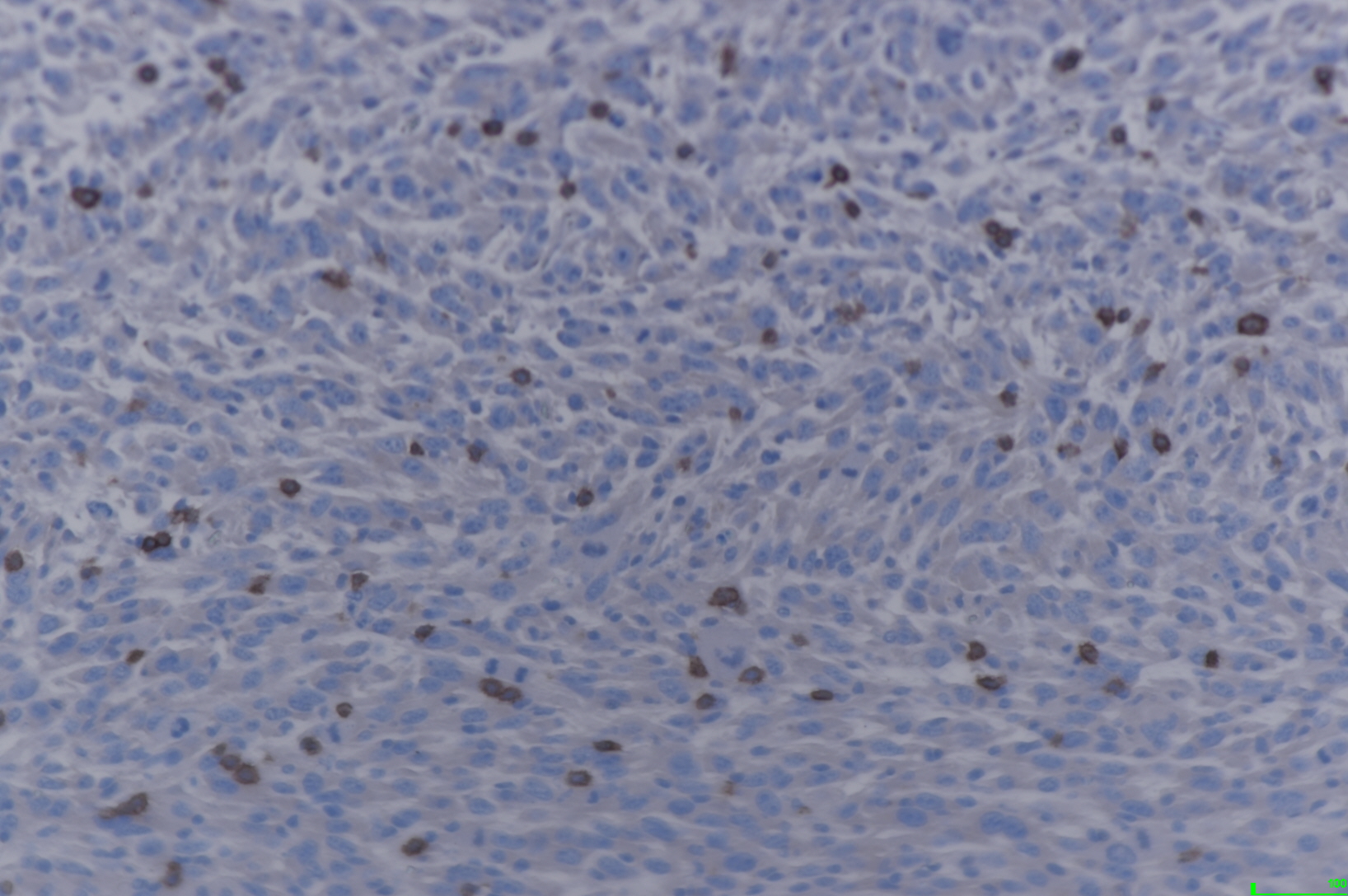

Supplement: Supplementary file 7 — Source data Fig. 5 [file 44321_2024_187_MOESM7_ESM.zip › Figure 5/5C/CD8 Control.jpg]

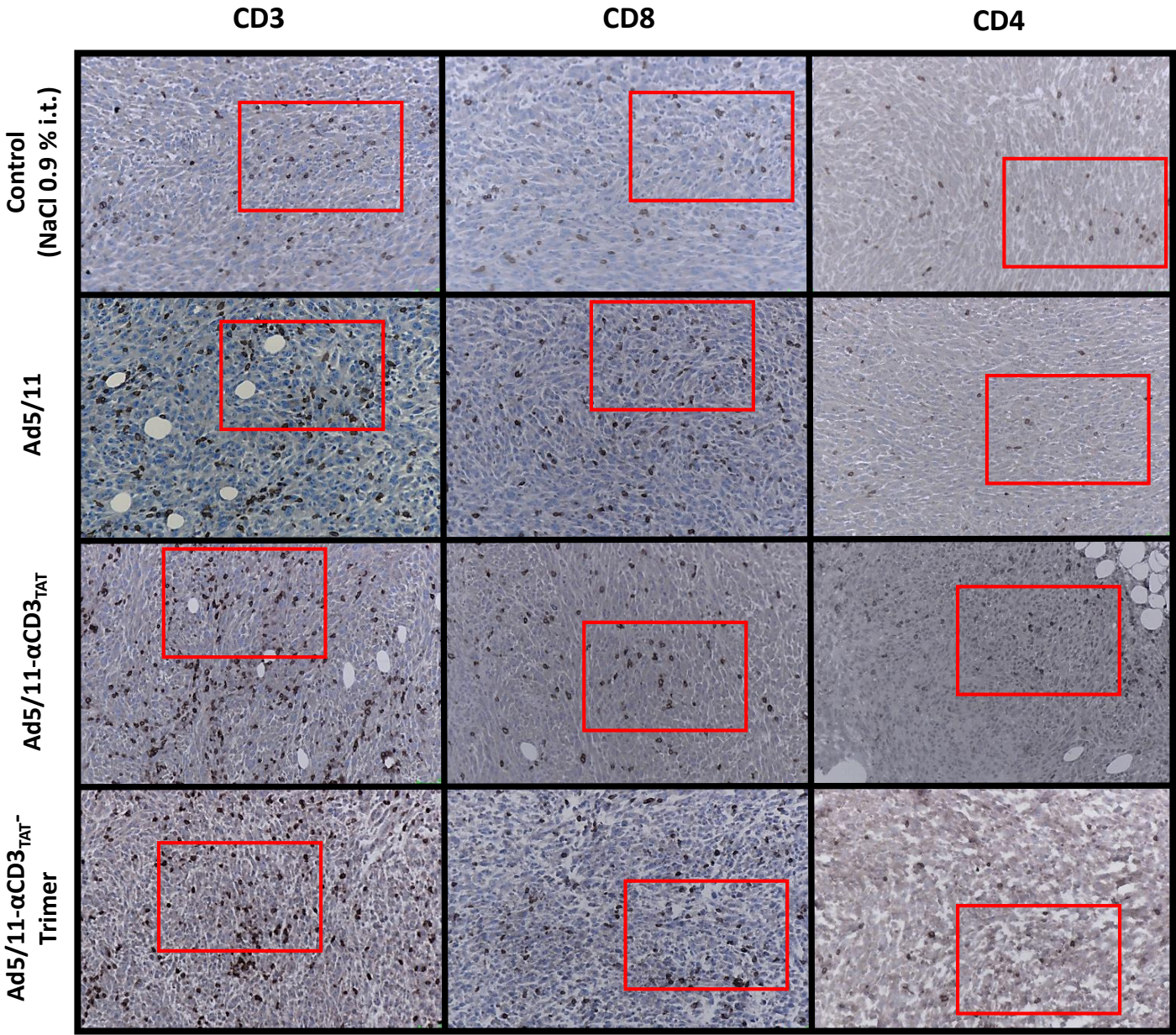

Supplement: Supplementary file 7 — Source data Fig. 5 [file 44321_2024_187_MOESM7_ESM.zip › Figure 5/5C/Fig5C_area of magnification.pdf]

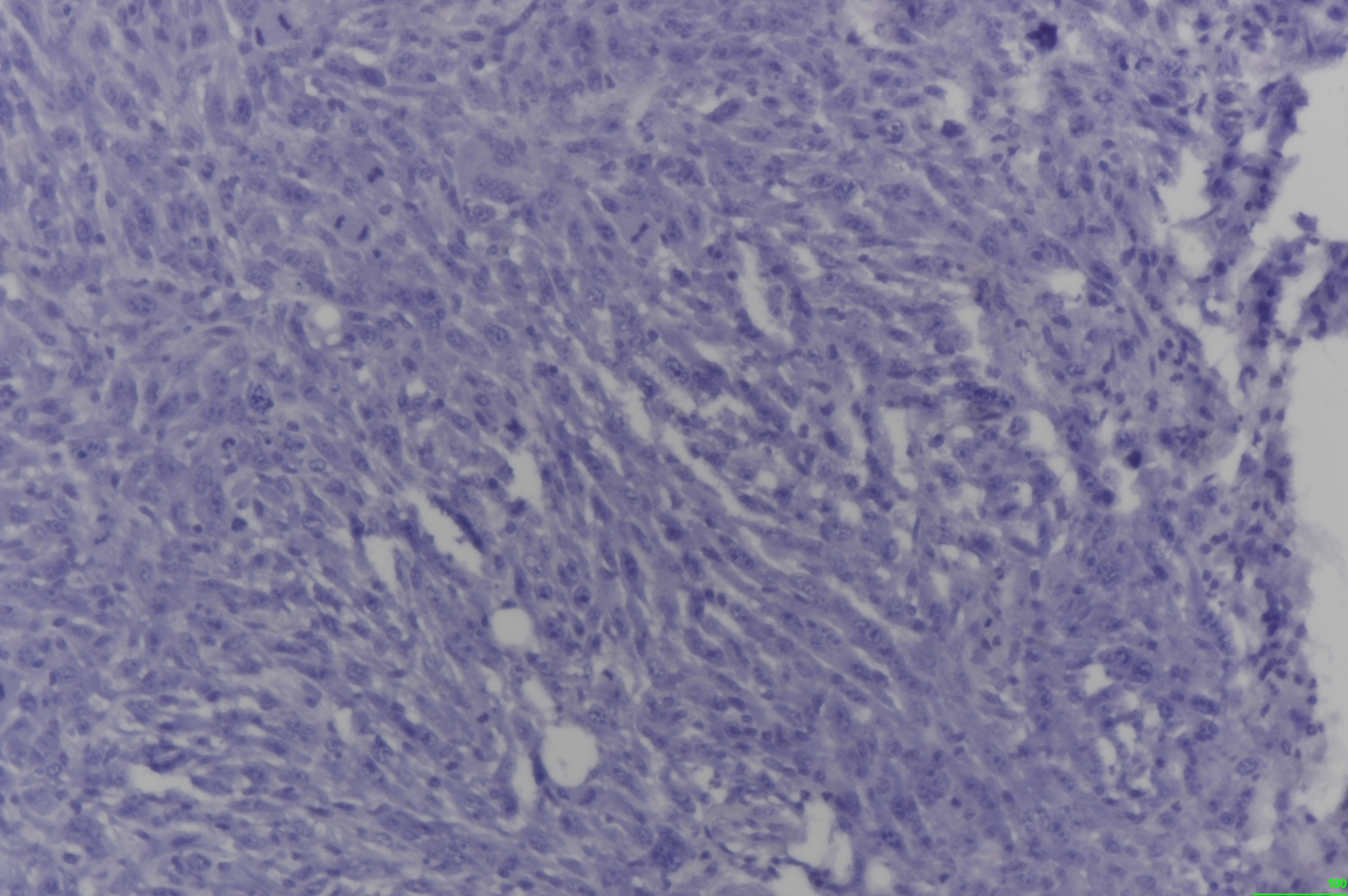

Supplement: Supplementary file 7 — Source data Fig. 5 [file 44321_2024_187_MOESM7_ESM.zip › Figure 5/5C/HE Ad5_11.jpg]

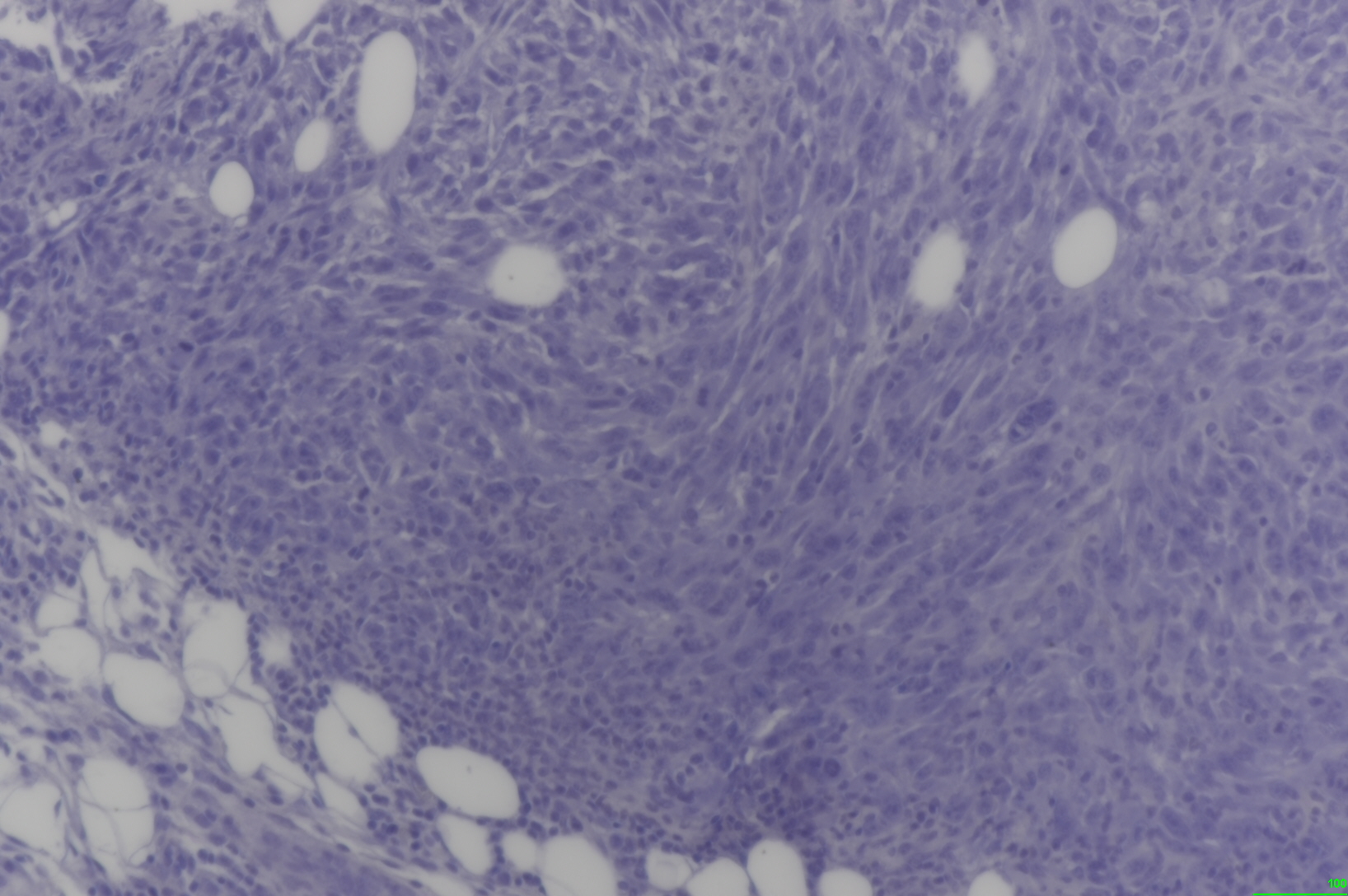

Supplement: Supplementary file 7 — Source data Fig. 5 [file 44321_2024_187_MOESM7_ESM.zip › Figure 5/5C/HE Ad5_11_CD3_TAT.jpg]

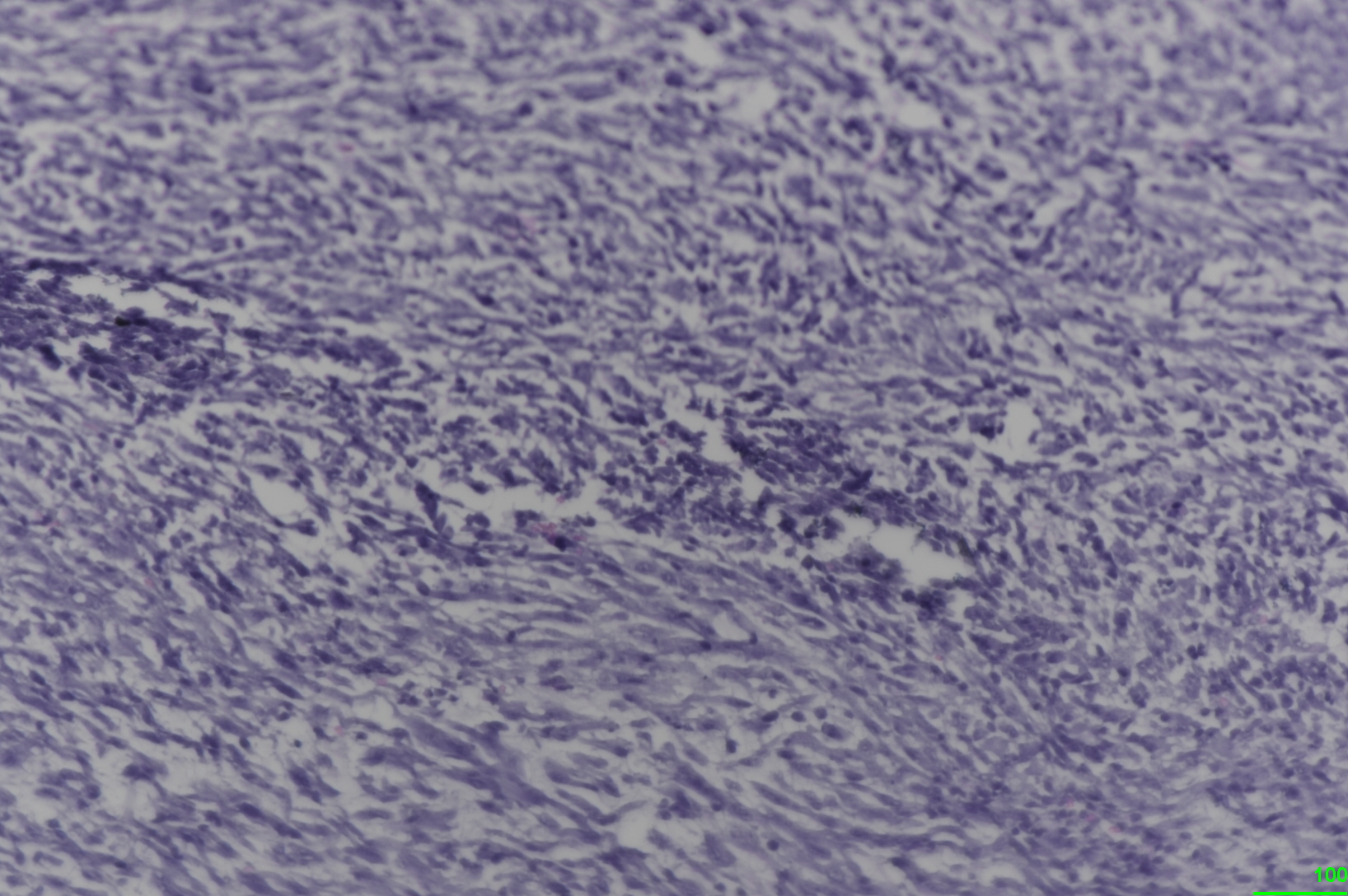

Supplement: Supplementary file 7 — Source data Fig. 5 [file 44321_2024_187_MOESM7_ESM.zip › Figure 5/5C/HE Ad5_11_CD3_TAT_Trimer.jpg]

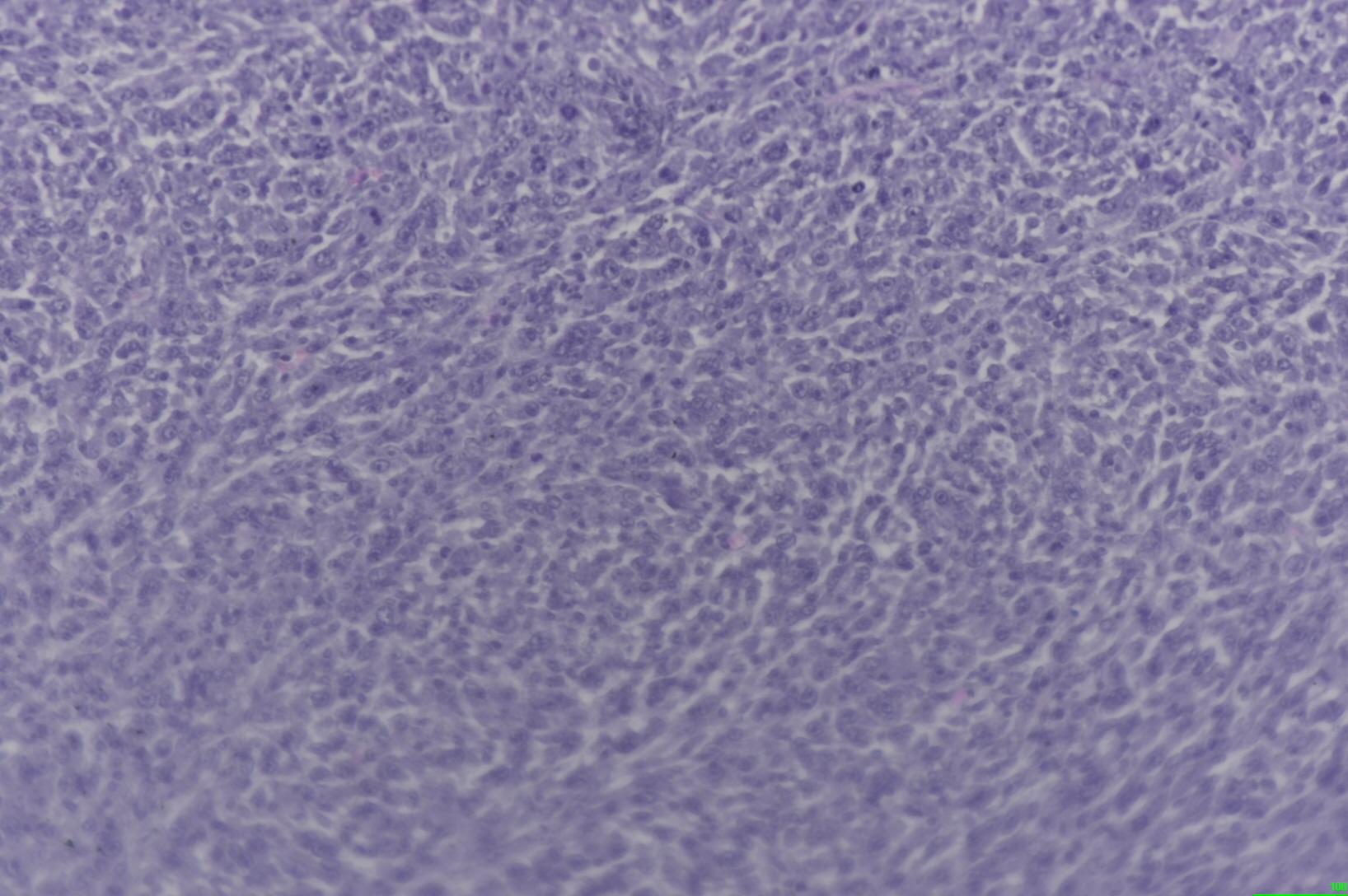

Supplement: Supplementary file 7 — Source data Fig. 5 [file 44321_2024_187_MOESM7_ESM.zip › Figure 5/5C/HE Control.jpg]

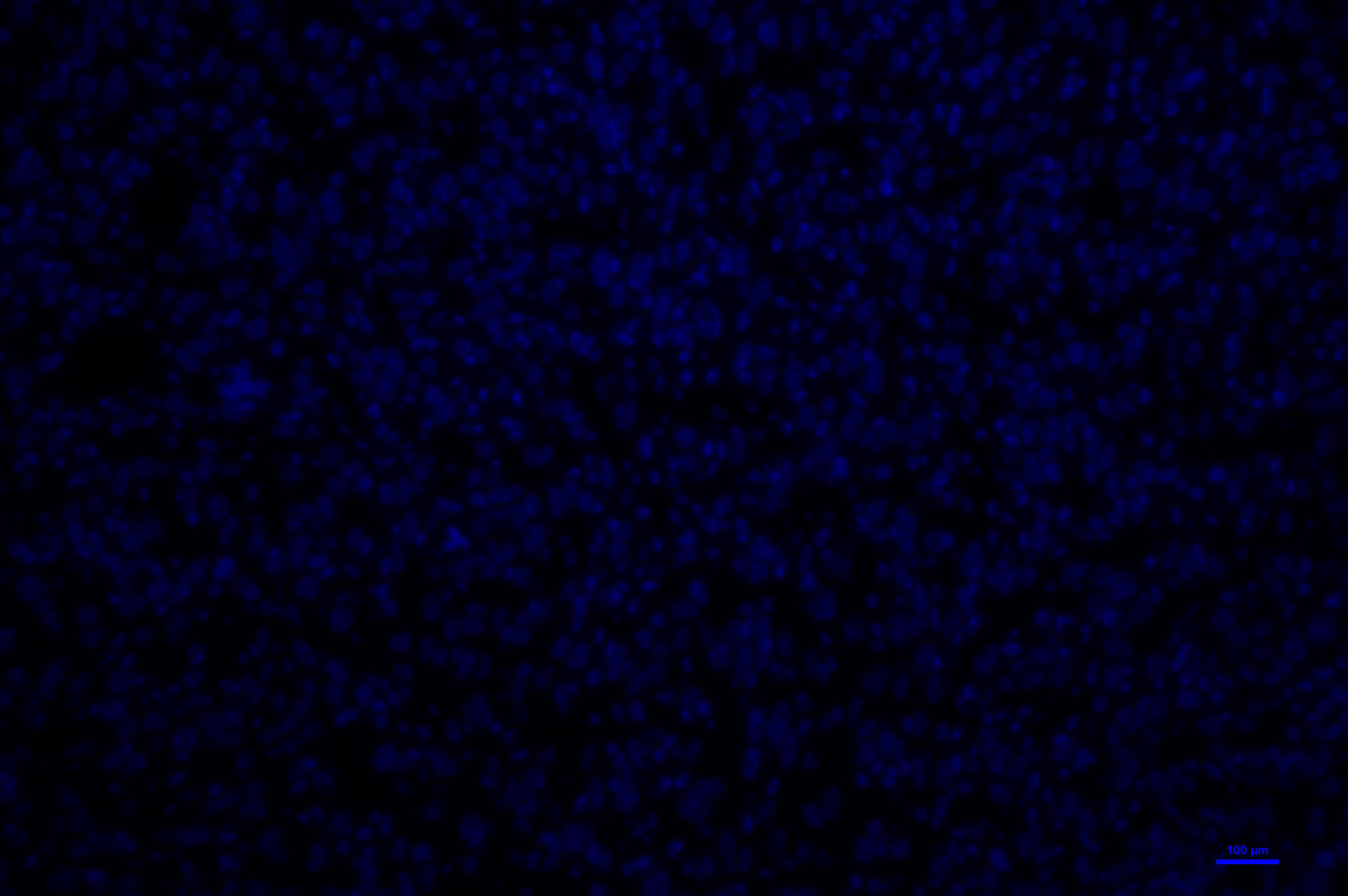

Supplement: Supplementary file 8 — Source data Fig. 6 [file 44321_2024_187_MOESM8_ESM.zip › Figure 6/6D/Ad5_11 20x_DAPi_2.jpg]

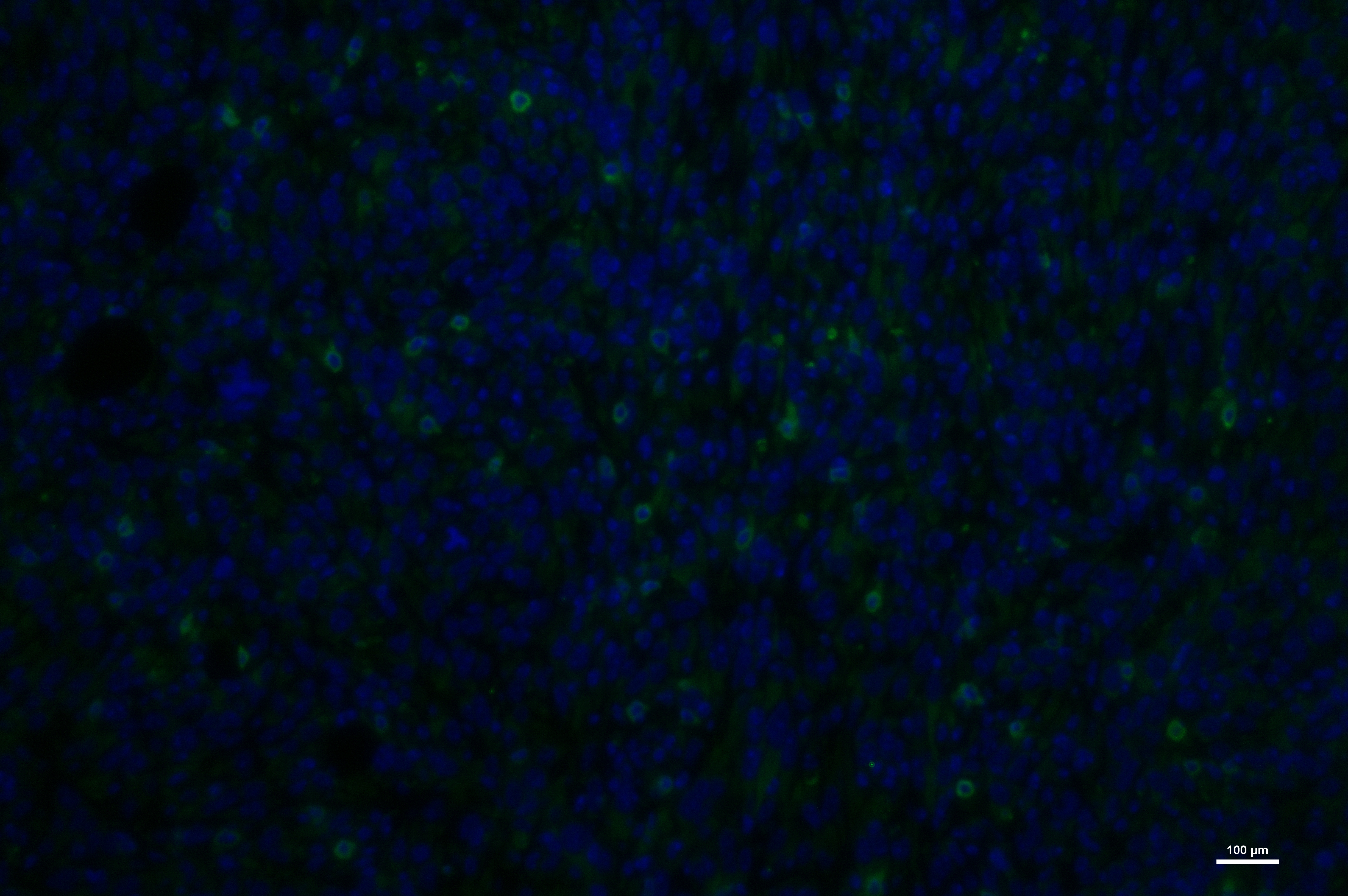

Supplement: Supplementary file 8 — Source data Fig. 6 [file 44321_2024_187_MOESM8_ESM.zip › Figure 6/6D/Ad5_11 20x_Multi_2.jpg]

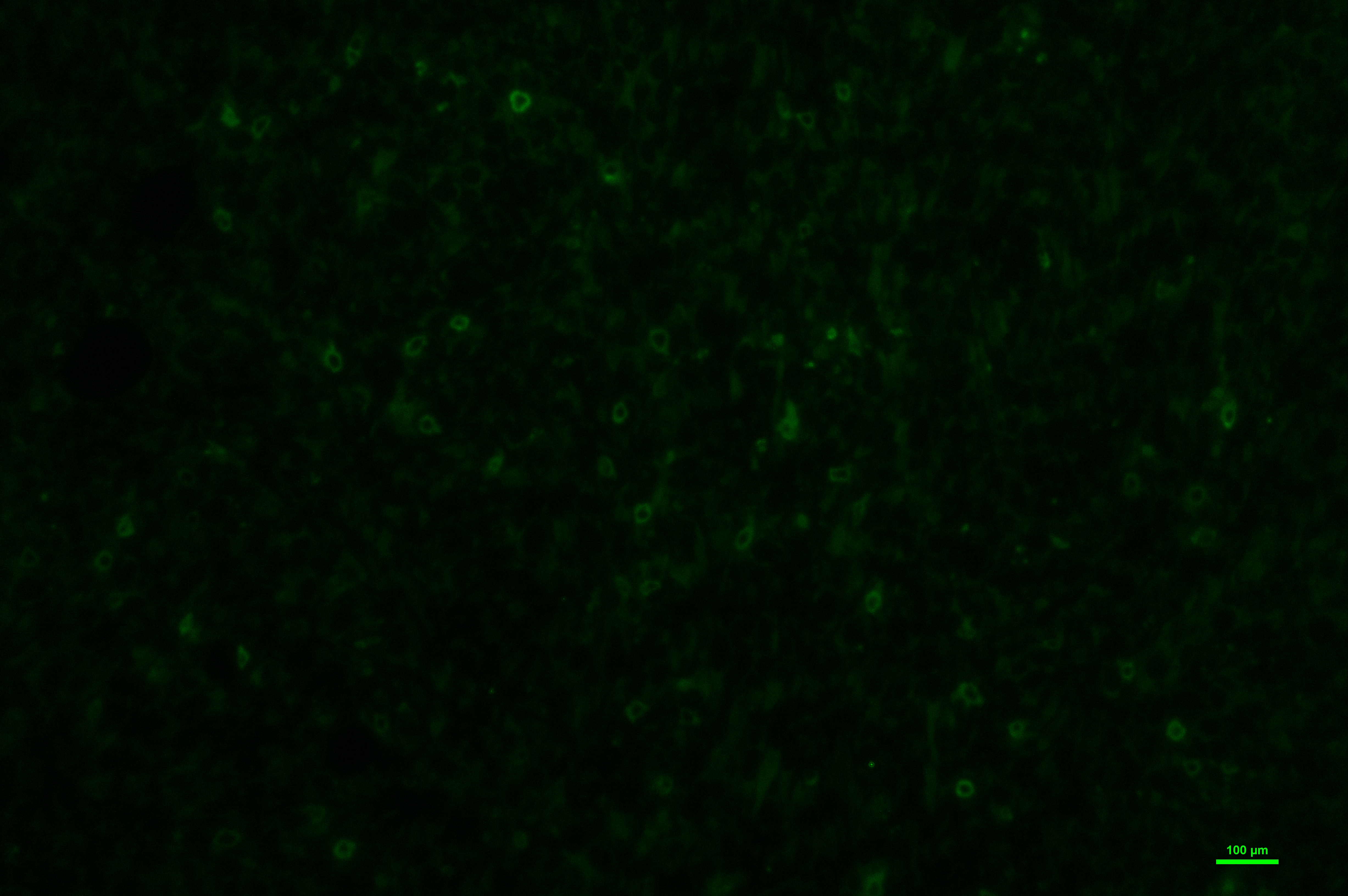

Supplement: Supplementary file 8 — Source data Fig. 6 [file 44321_2024_187_MOESM8_ESM.zip › Figure 6/6D/Ad5_11 20x_PD1_2.jpg]

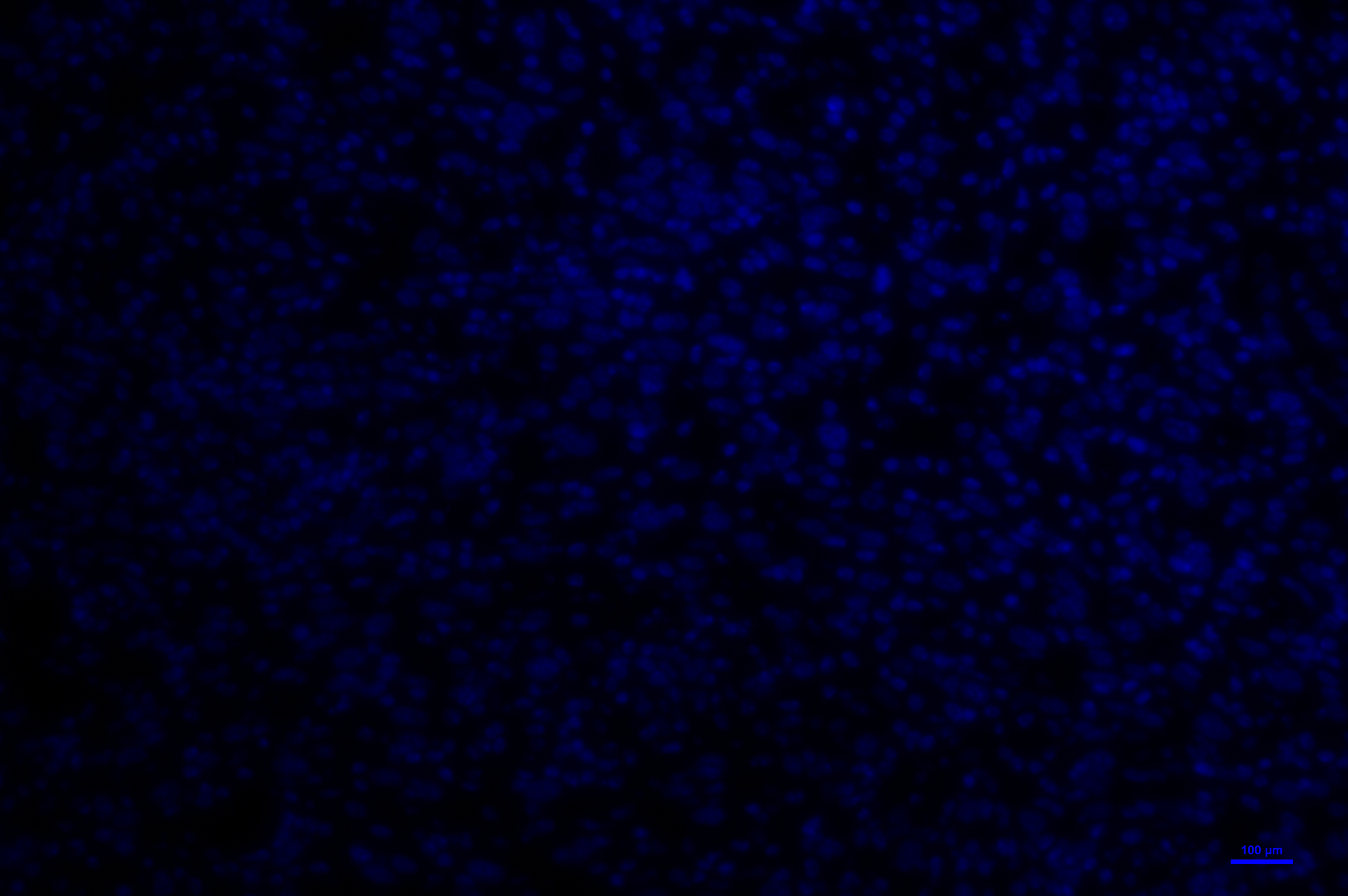

Supplement: Supplementary file 8 — Source data Fig. 6 [file 44321_2024_187_MOESM8_ESM.zip › Figure 6/6D/Ad5_11_CD3_TAT 20x_DAPI.jpg]

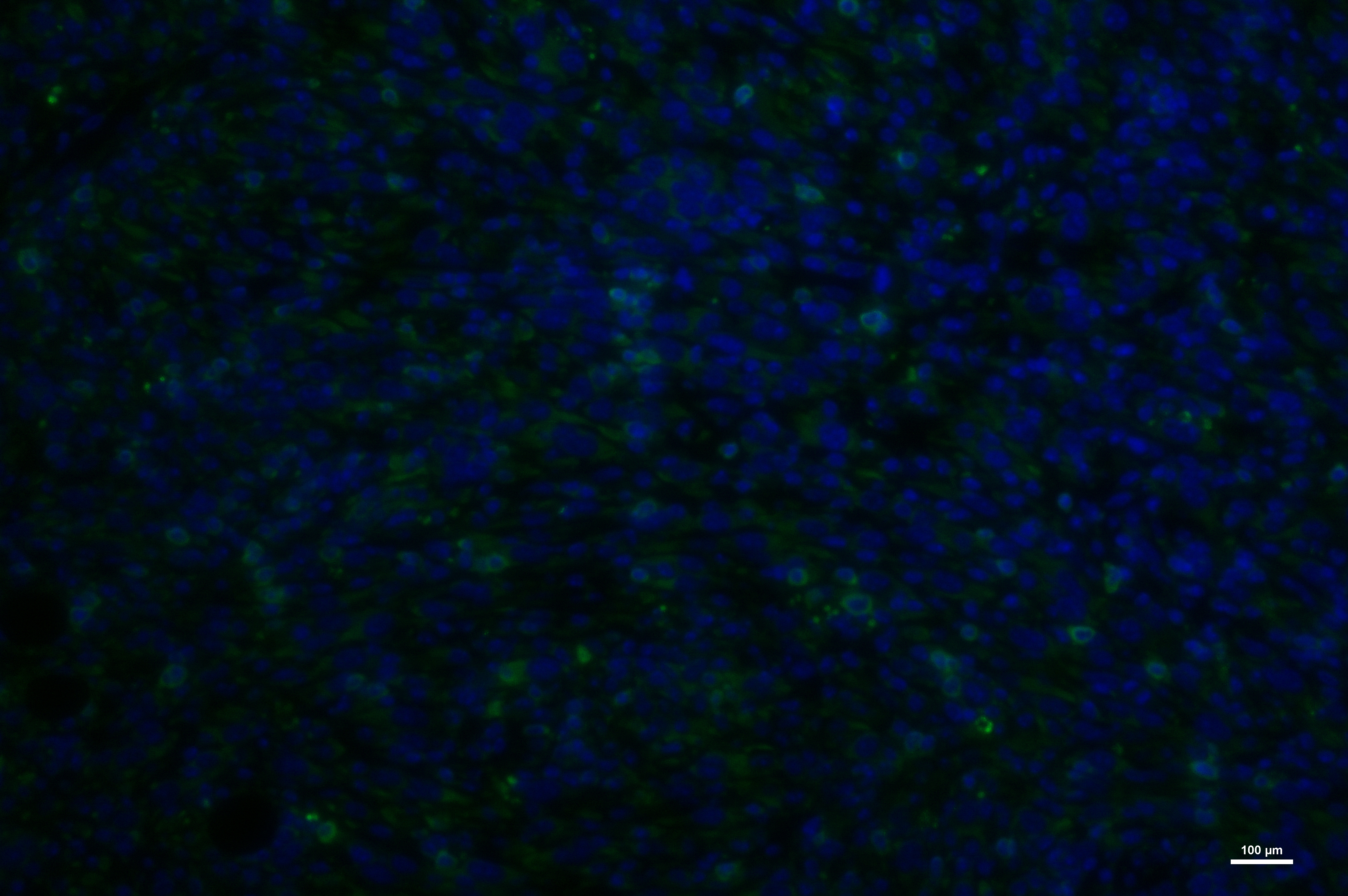

Supplement: Supplementary file 8 — Source data Fig. 6 [file 44321_2024_187_MOESM8_ESM.zip › Figure 6/6D/Ad5_11_CD3_TAT 20x_Multich.jpg]

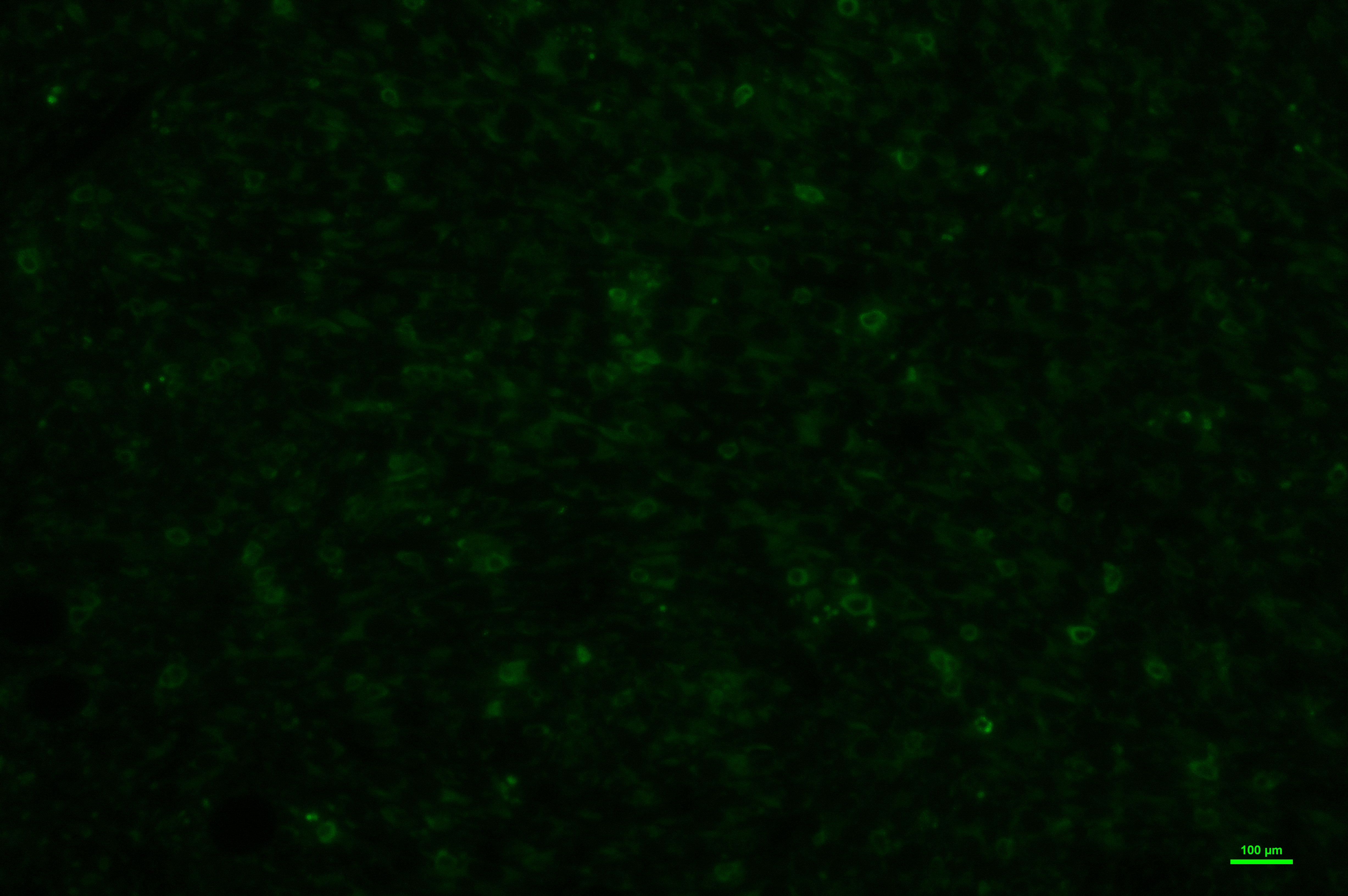

Supplement: Supplementary file 8 — Source data Fig. 6 [file 44321_2024_187_MOESM8_ESM.zip › Figure 6/6D/Ad5_11_CD3_TAT 20x_PD1.jpg]

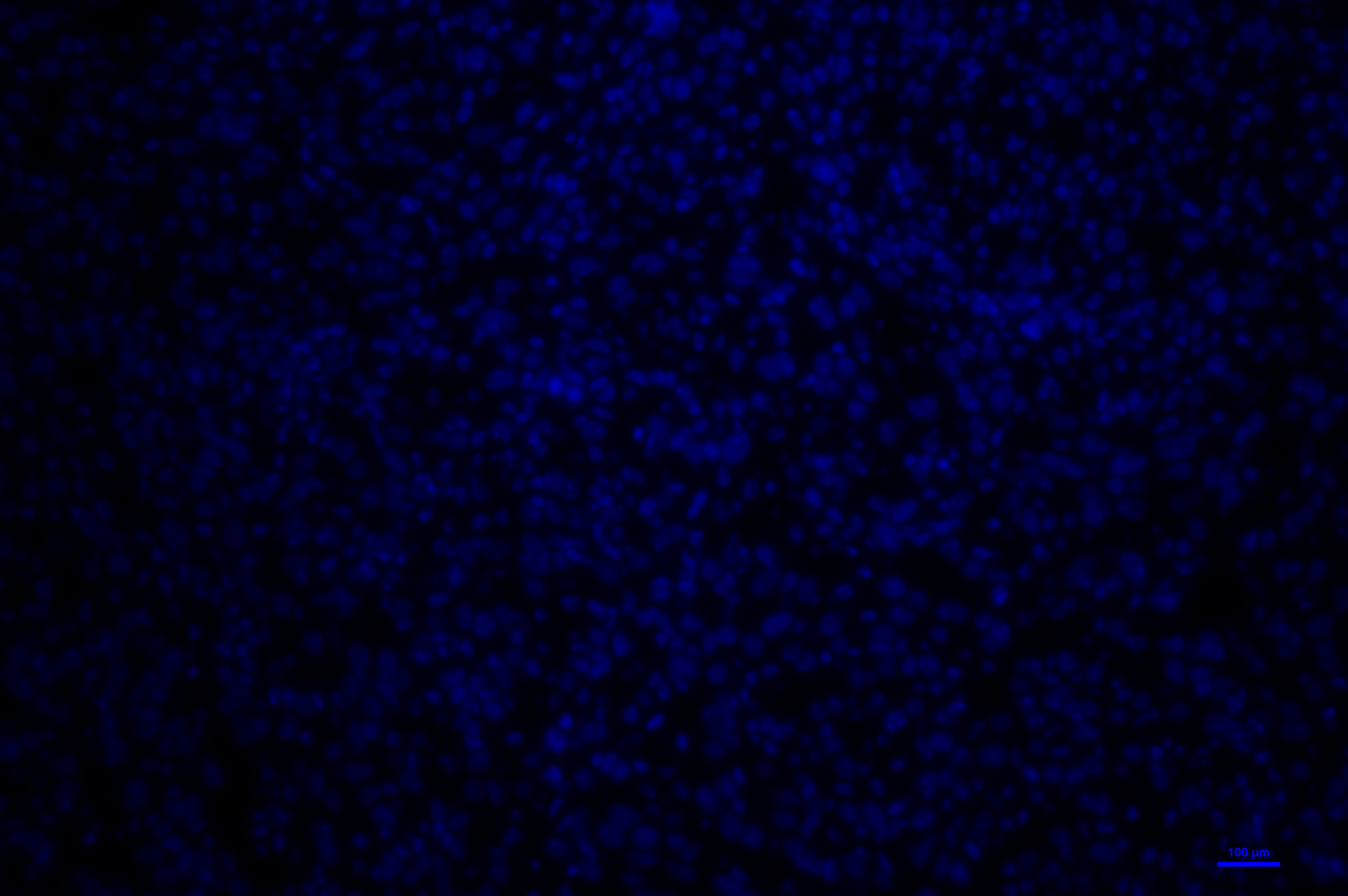

Supplement: Supplementary file 8 — Source data Fig. 6 [file 44321_2024_187_MOESM8_ESM.zip › Figure 6/6D/Ad5_11_CD3_TAT_Trimer 20x_DAPI.jpg]

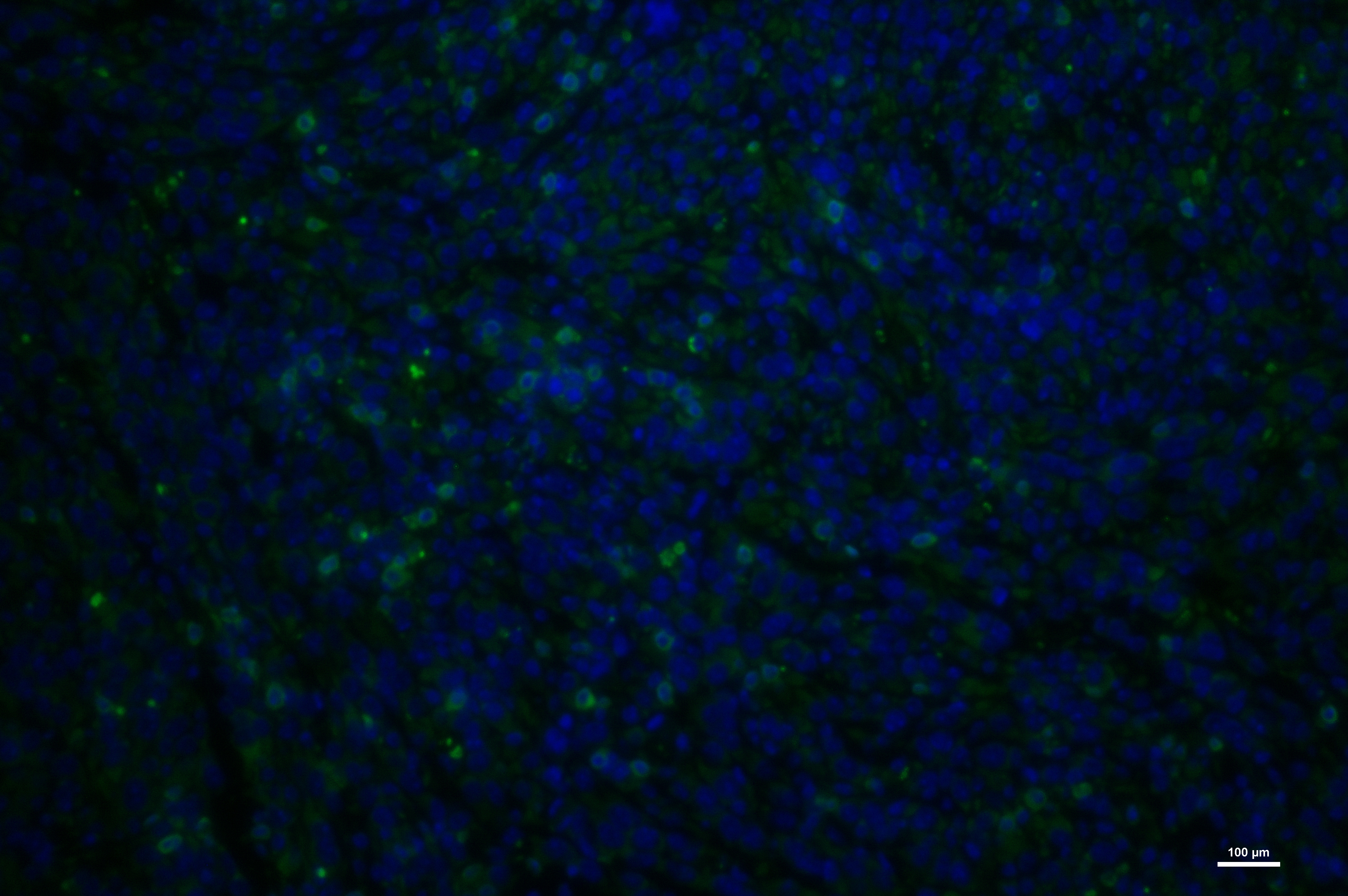

Supplement: Supplementary file 8 — Source data Fig. 6 [file 44321_2024_187_MOESM8_ESM.zip › Figure 6/6D/Ad5_11_CD3_TAT_Trimer 20x_Multich.jpg]

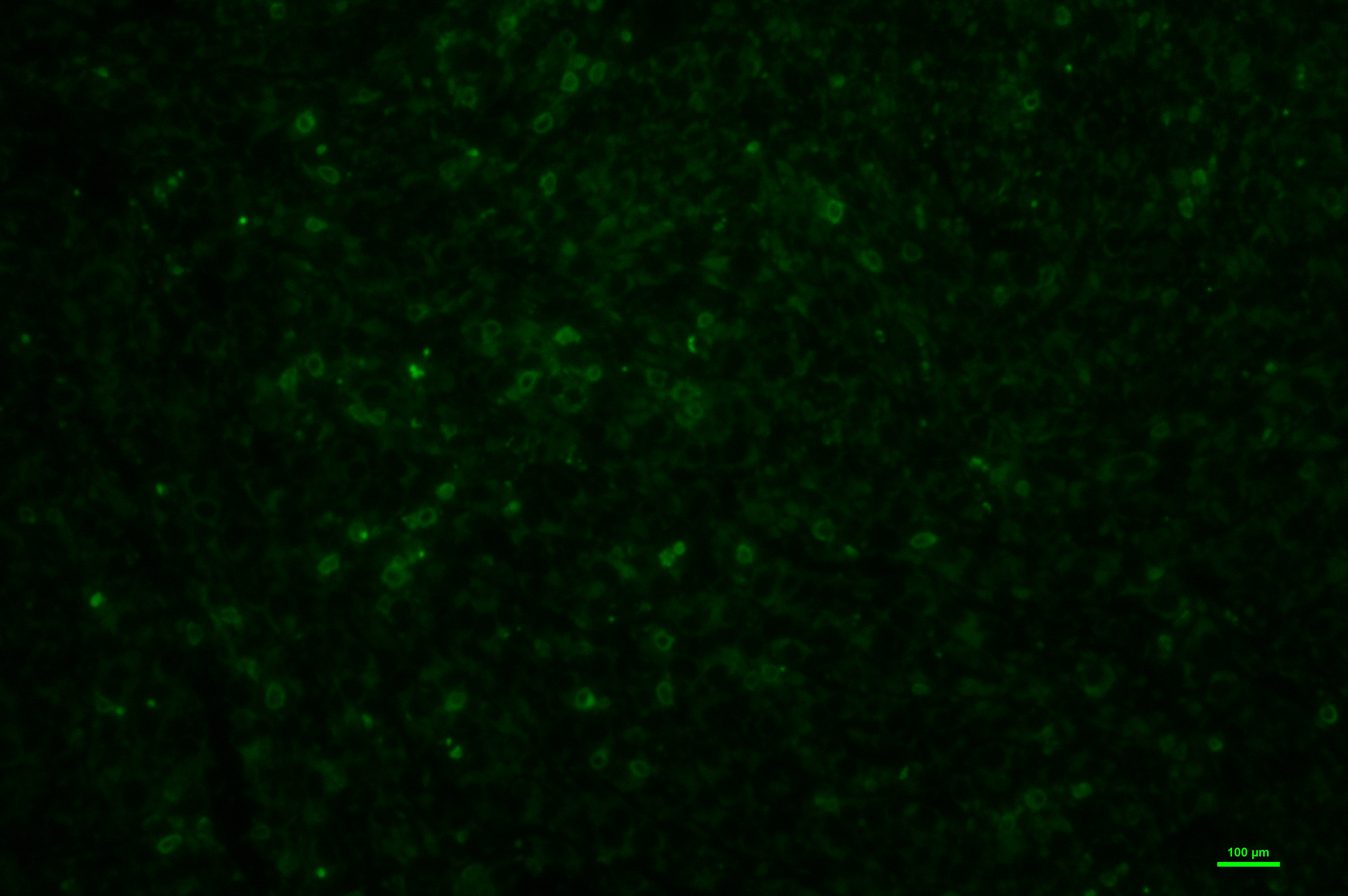

Supplement: Supplementary file 8 — Source data Fig. 6 [file 44321_2024_187_MOESM8_ESM.zip › Figure 6/6D/Ad5_11_CD3_TAT_Trimer 20x_PD1.jpg]

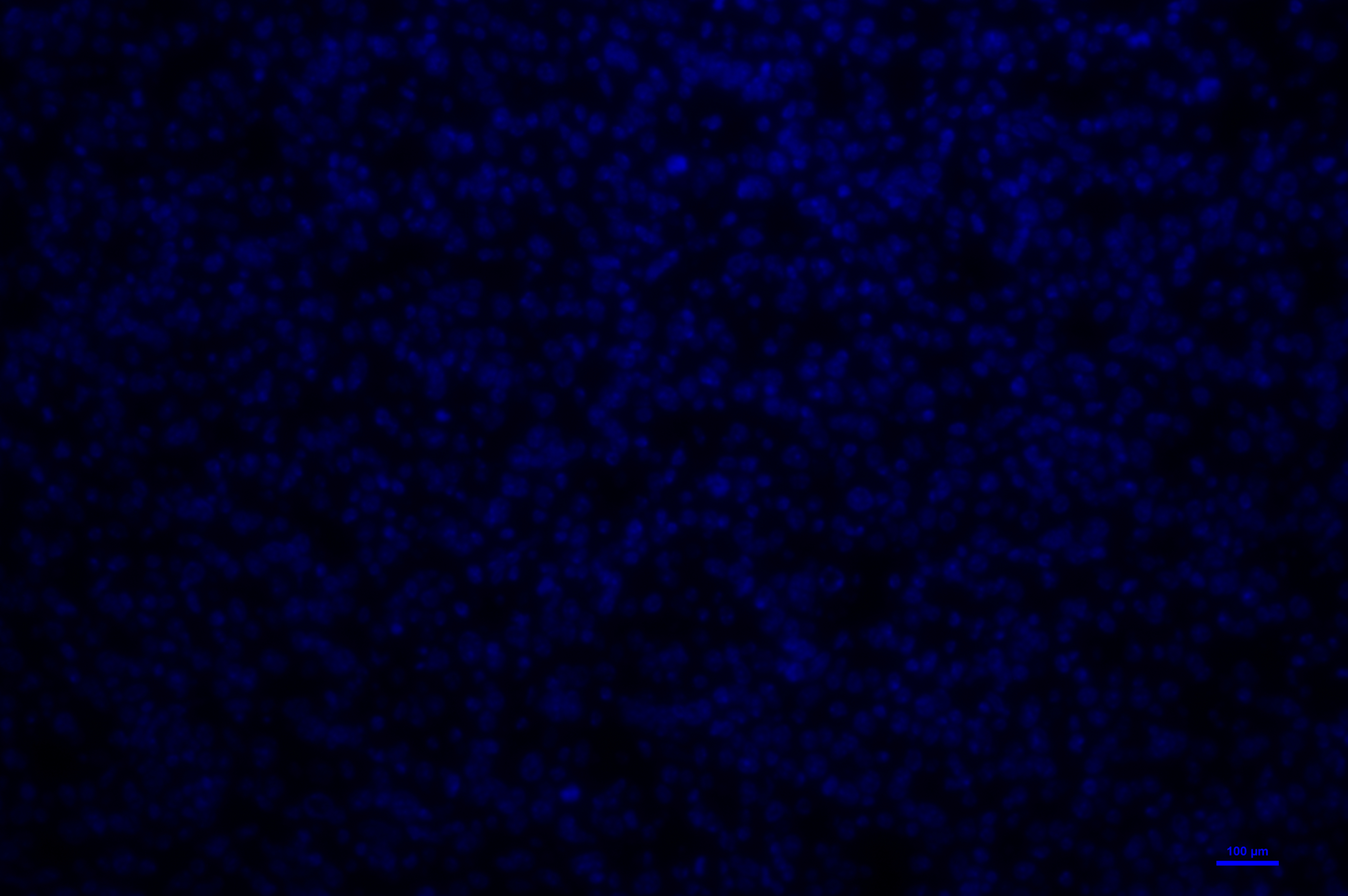

Supplement: Supplementary file 8 — Source data Fig. 6 [file 44321_2024_187_MOESM8_ESM.zip › Figure 6/6D/control 20x_DAPi.jpg]

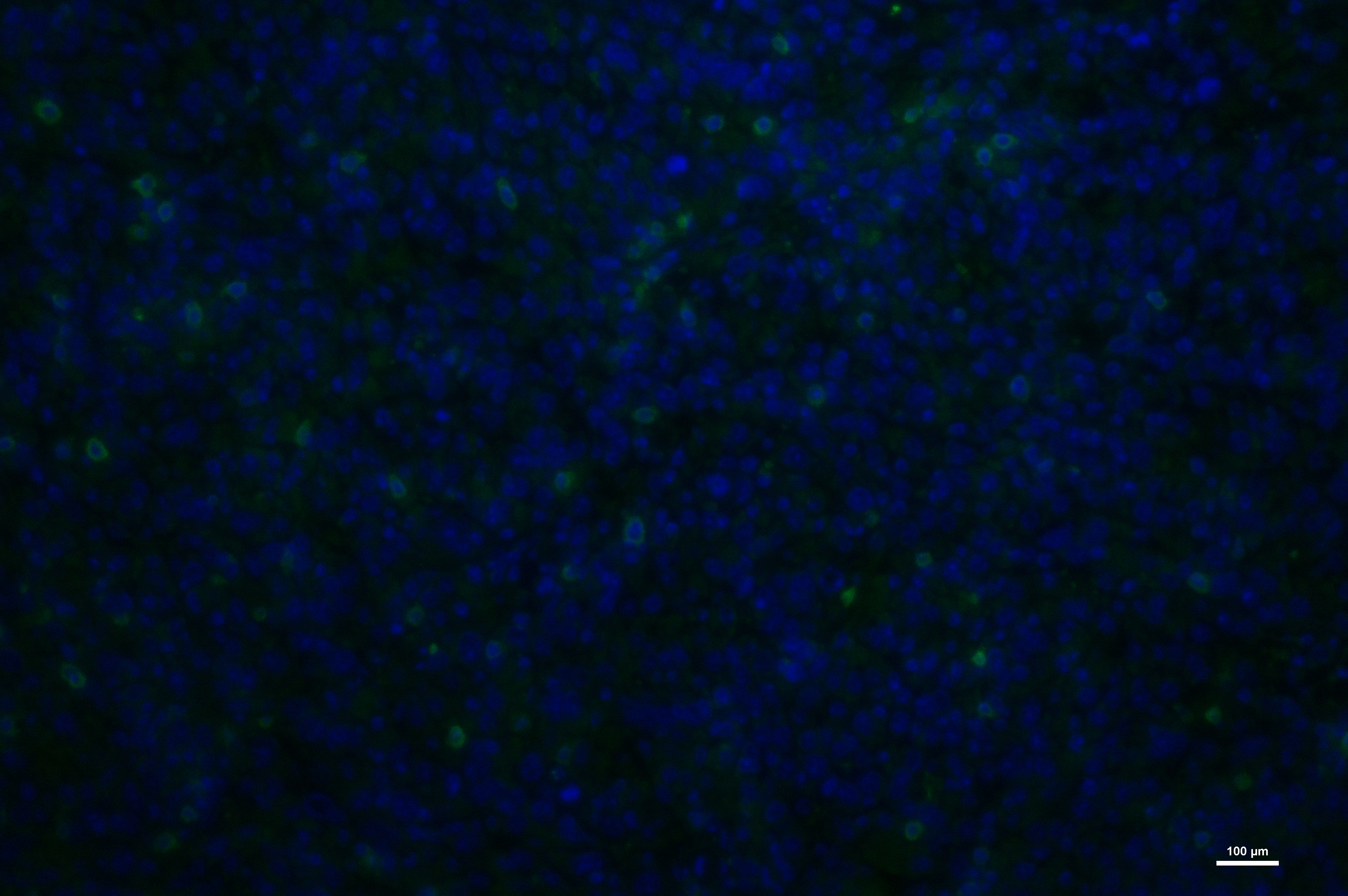

Supplement: Supplementary file 8 — Source data Fig. 6 [file 44321_2024_187_MOESM8_ESM.zip › Figure 6/6D/control 20x_Multi.jpg]

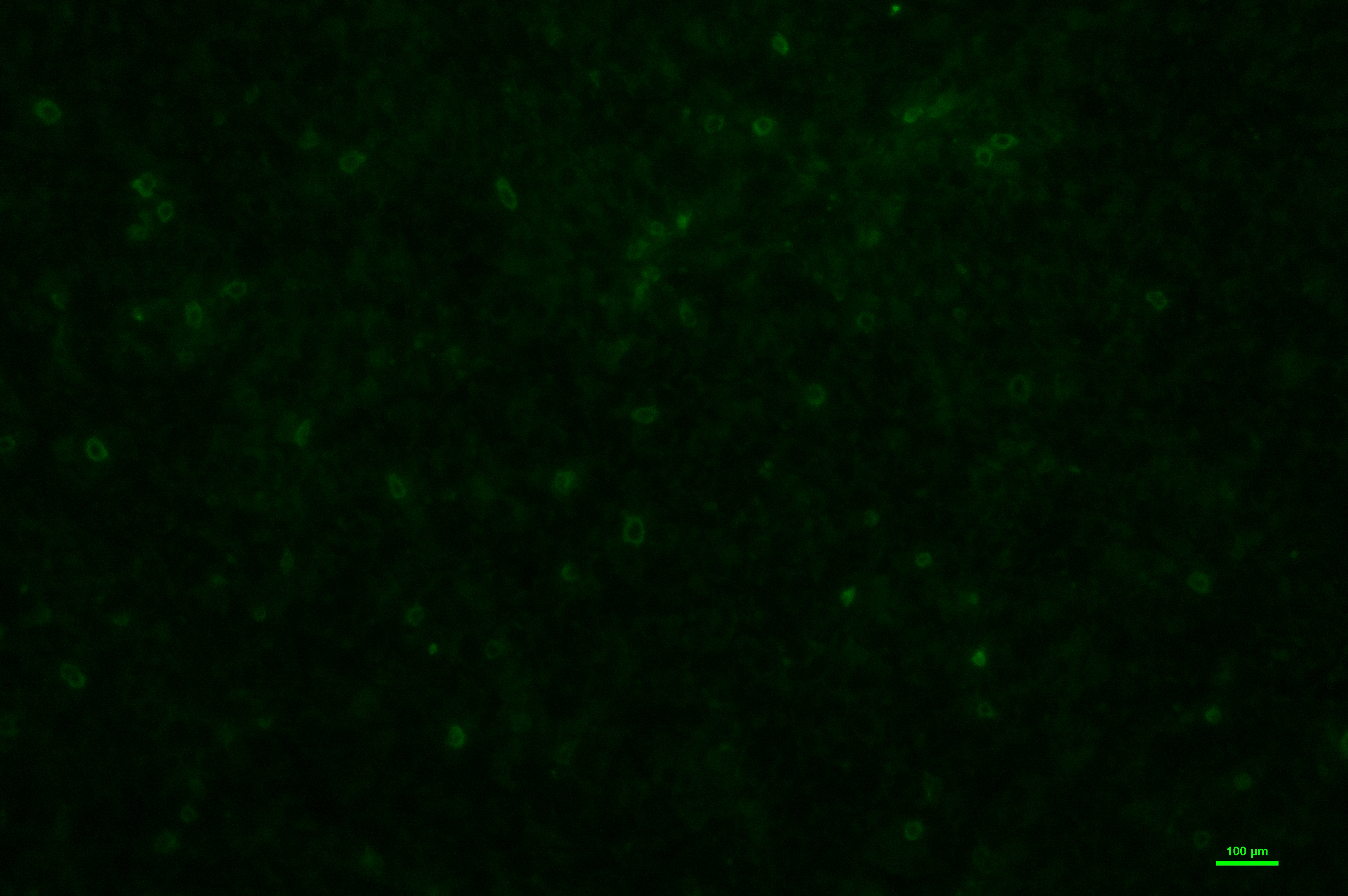

Supplement: Supplementary file 8 — Source data Fig. 6 [file 44321_2024_187_MOESM8_ESM.zip › Figure 6/6D/control 20x_PD1.jpg]

**Control**  
**(NaCl 0.9 % i.t.)**

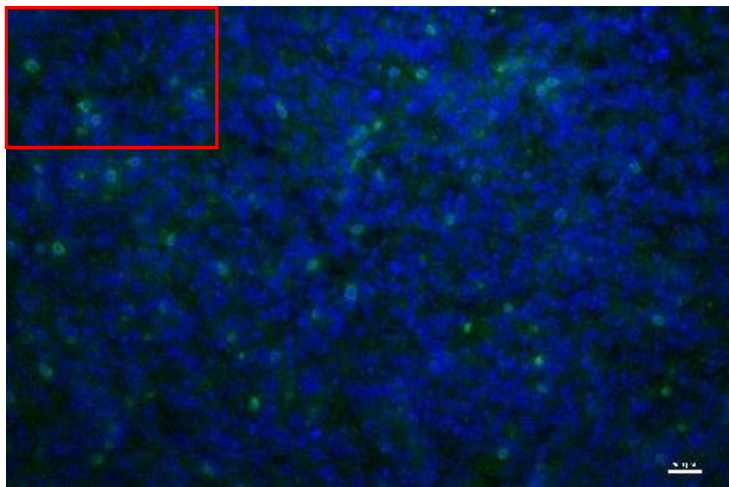

**Ad5/11**

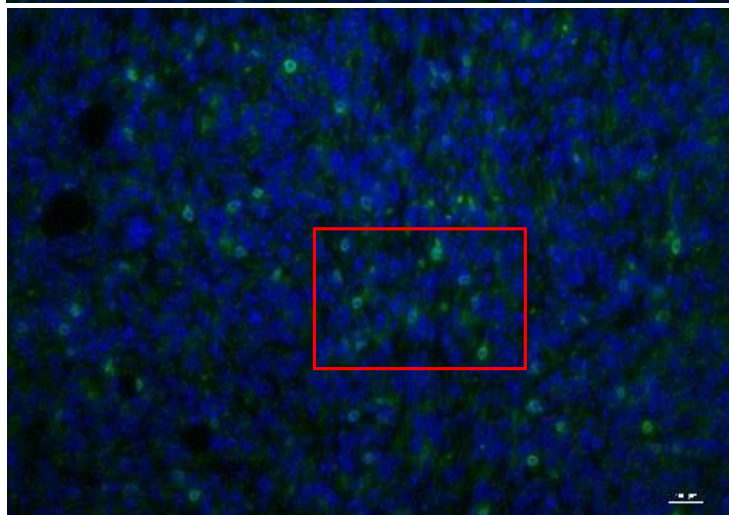

**Ad5/11- $\alpha$ CD3<sub>TAT</sub>**

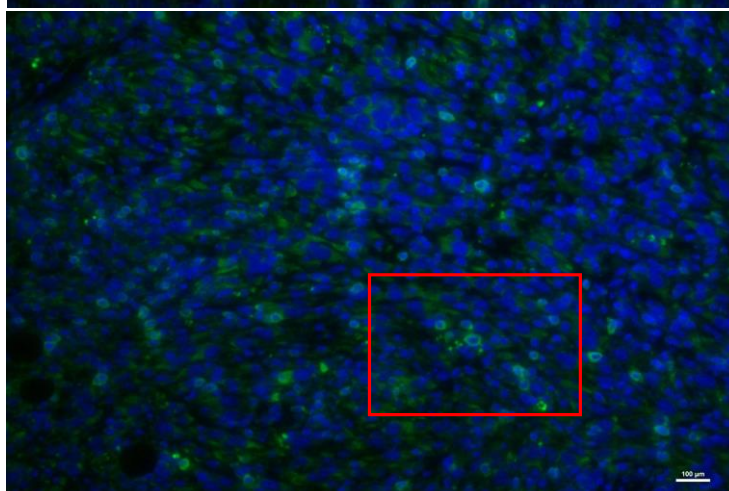

**Ad5/11- $\alpha$ CD3<sub>TAT</sub>-  
Trimer**

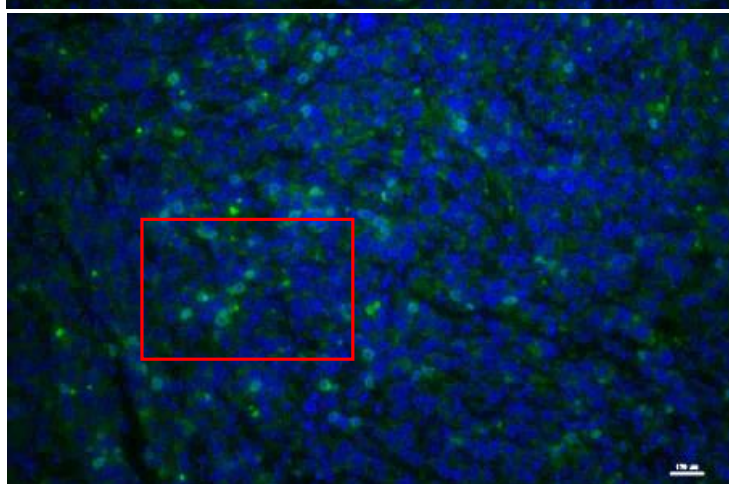

Supplement: Supplementary file 8 — Source data Fig. 6 [file 44321_2024_187_MOESM8_ESM.zip › Figure 6/6D/Fig6D_area of magnification.pdf]

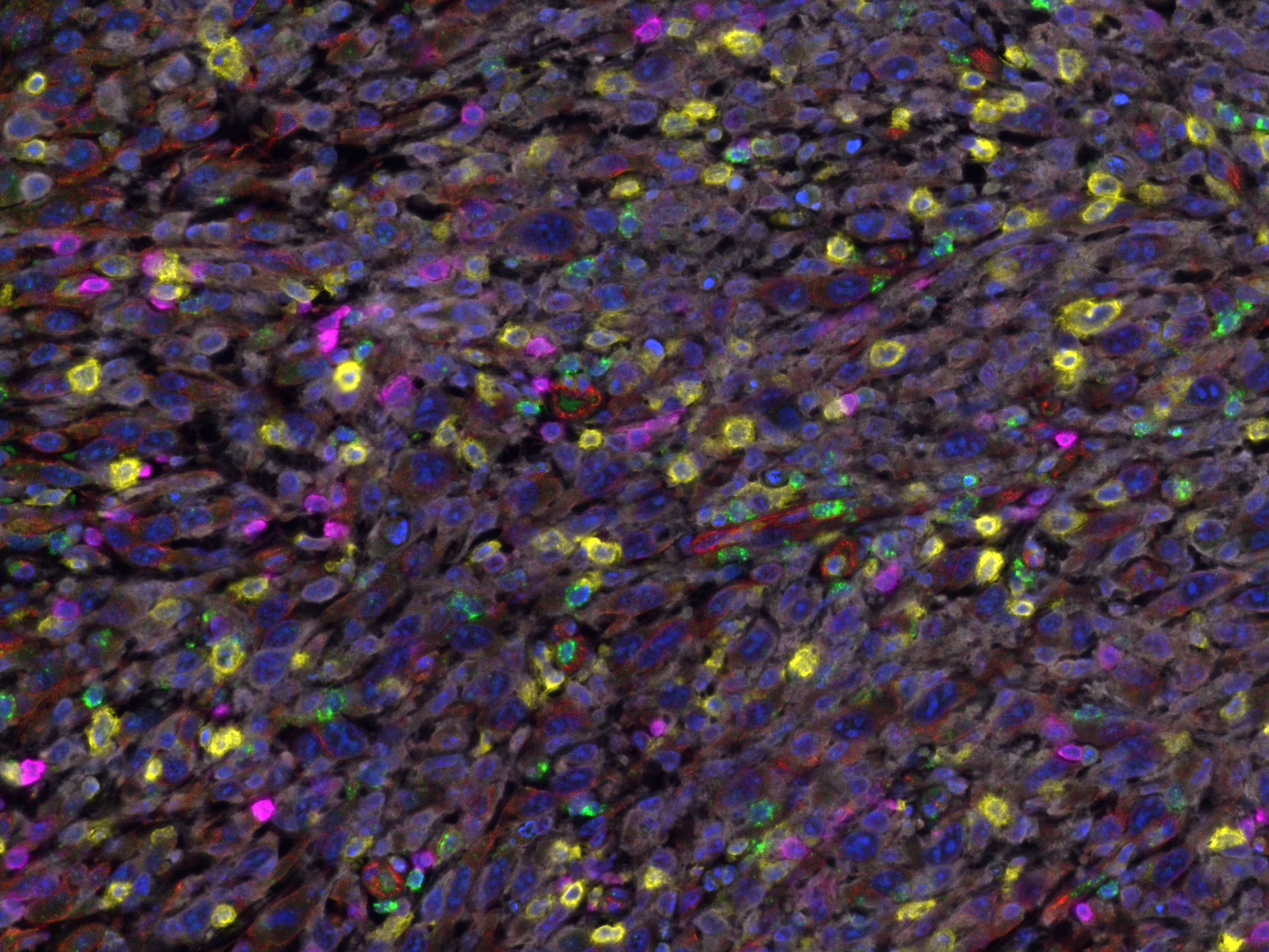

Supplement: Supplementary file 10 — Source data Fig. 8 [file 44321_2024_187_MOESM10_ESM.zip › Figure 8/8A/aPD1+Ad5-11-Trimer_K17709_[16651,46771]_composite_image.tif]

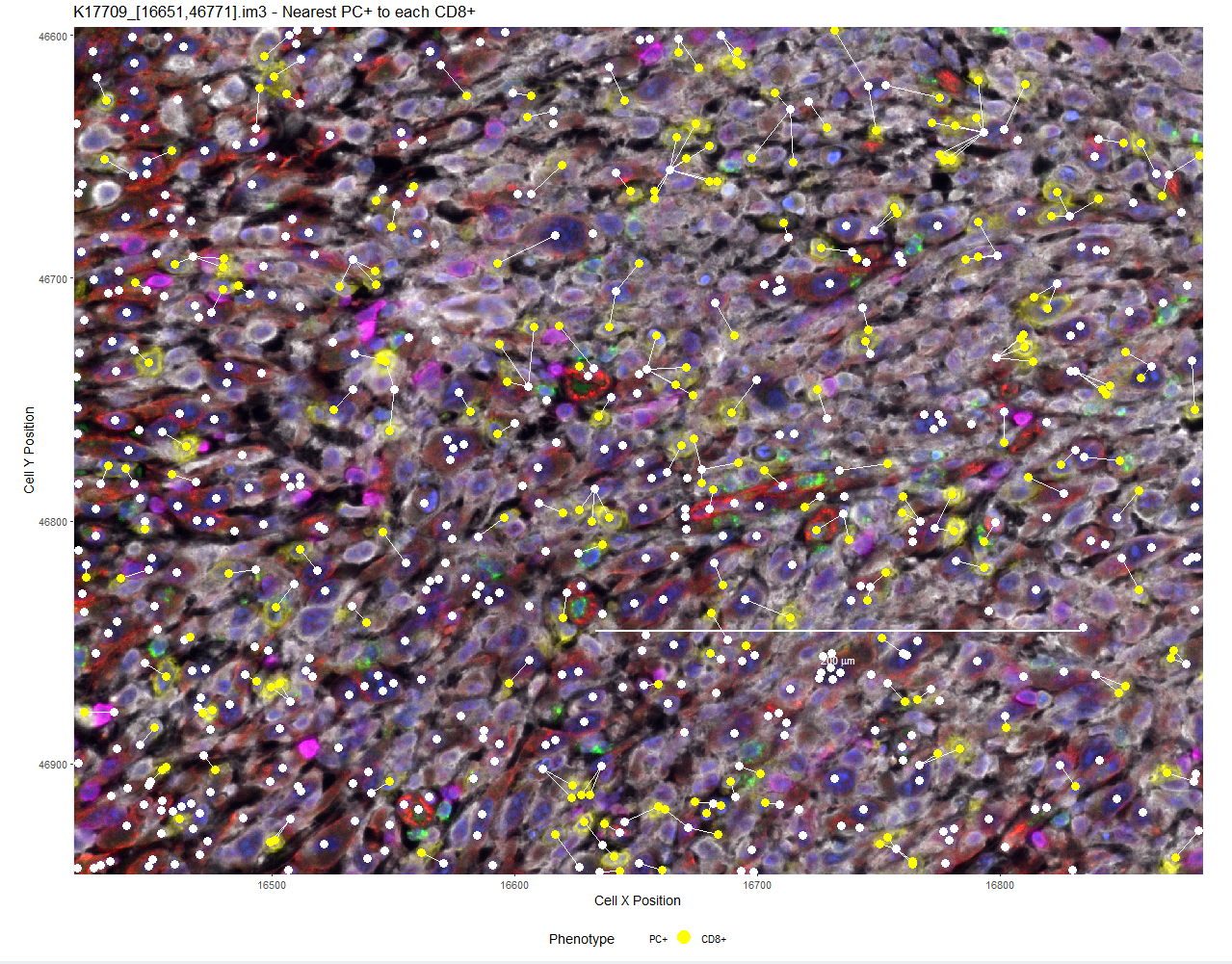

Supplement: Supplementary file 10 — Source data Fig. 8 [file 44321_2024_187_MOESM10_ESM.zip › Figure 8/8A/aPD1+Ad5-11-Trimer_K17709_[16651,46771]_PC+_near_CD8+.tif]

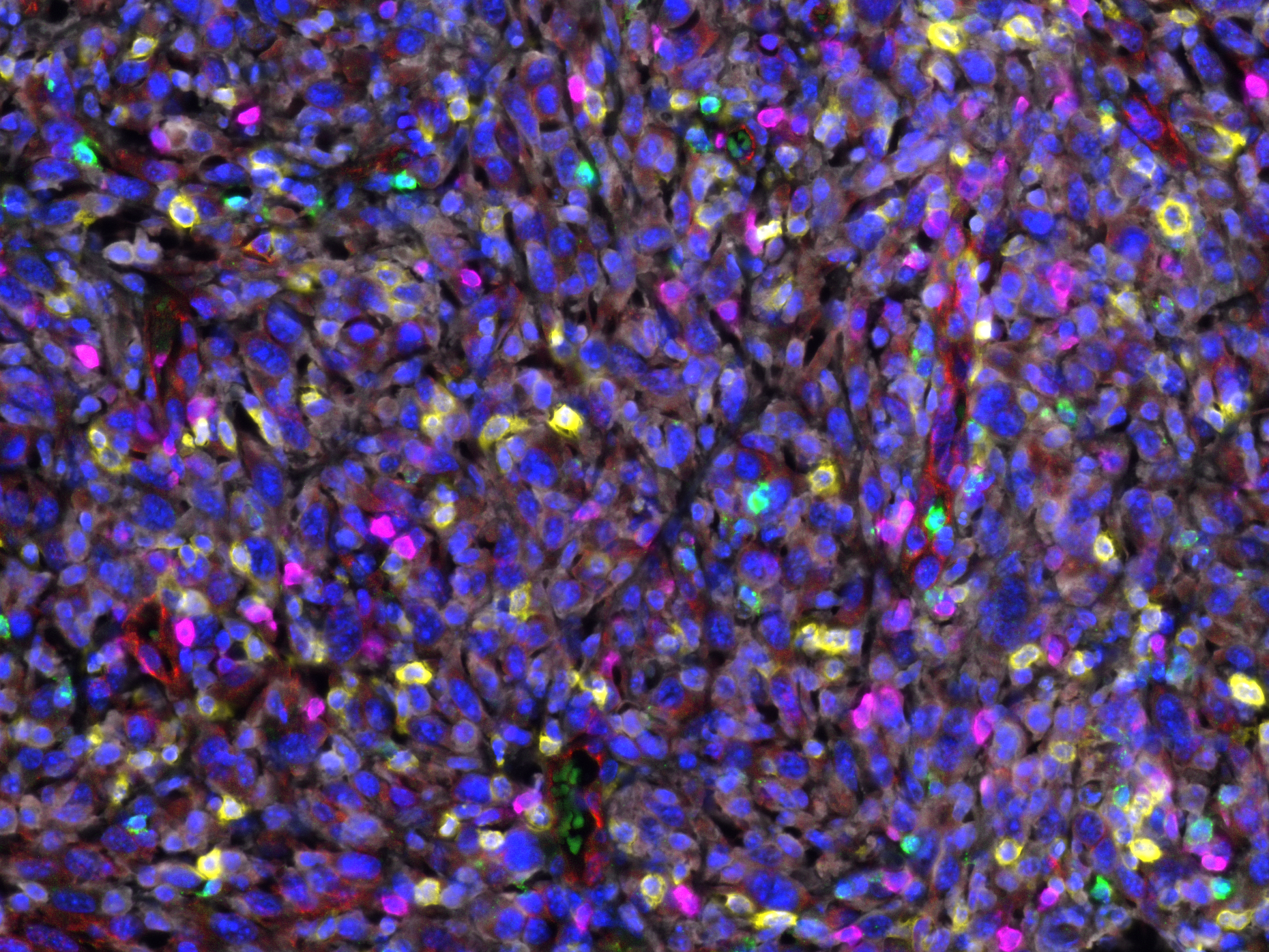

Supplement: Supplementary file 10 — Source data Fig. 8 [file 44321_2024_187_MOESM10_ESM.zip › Figure 8/8A/aPD1+Ad5-11_K17727_[15893,42754]_composite_image.tif]

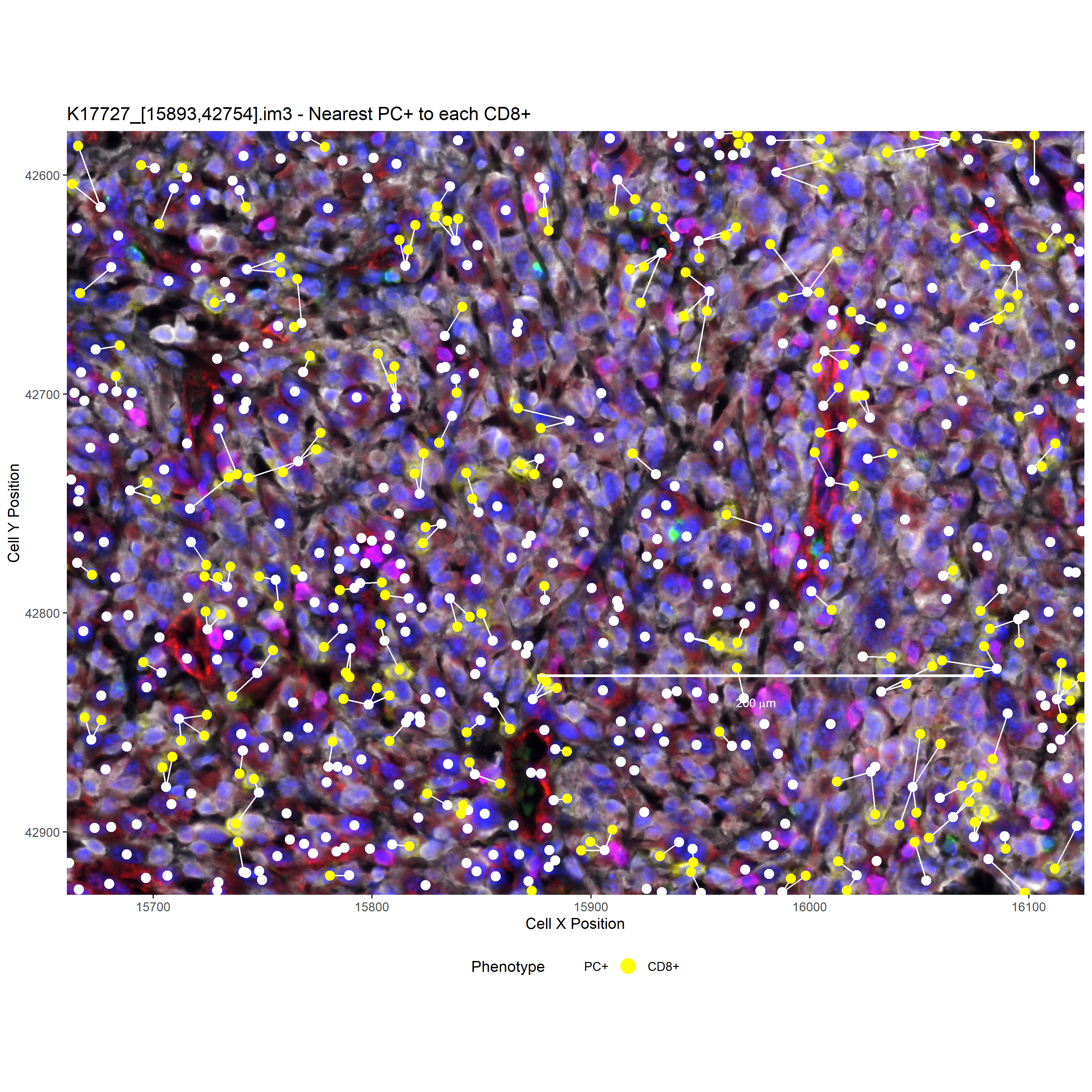

Supplement: Supplementary file 10 — Source data Fig. 8 [file 44321_2024_187_MOESM10_ESM.zip › Figure 8/8A/aPD1+Ad5-11_K17727_[15893,42754]_PC+_near_CD8+.png]

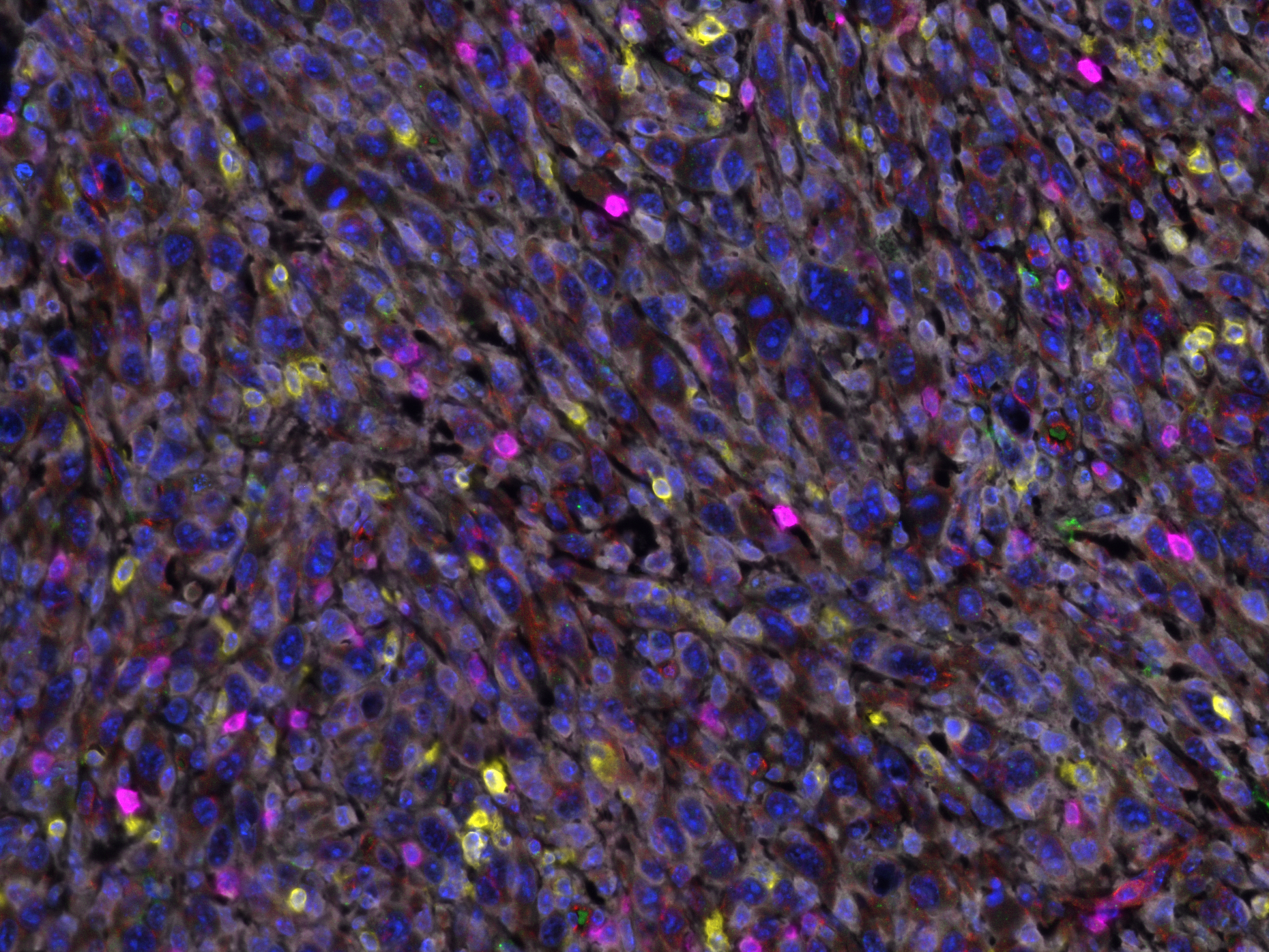

Supplement: Supplementary file 10 — Source data Fig. 8 [file 44321_2024_187_MOESM10_ESM.zip › Figure 8/8A/aPD1_Ctrl_K17704_[8280,53501]_composite_image.tif]

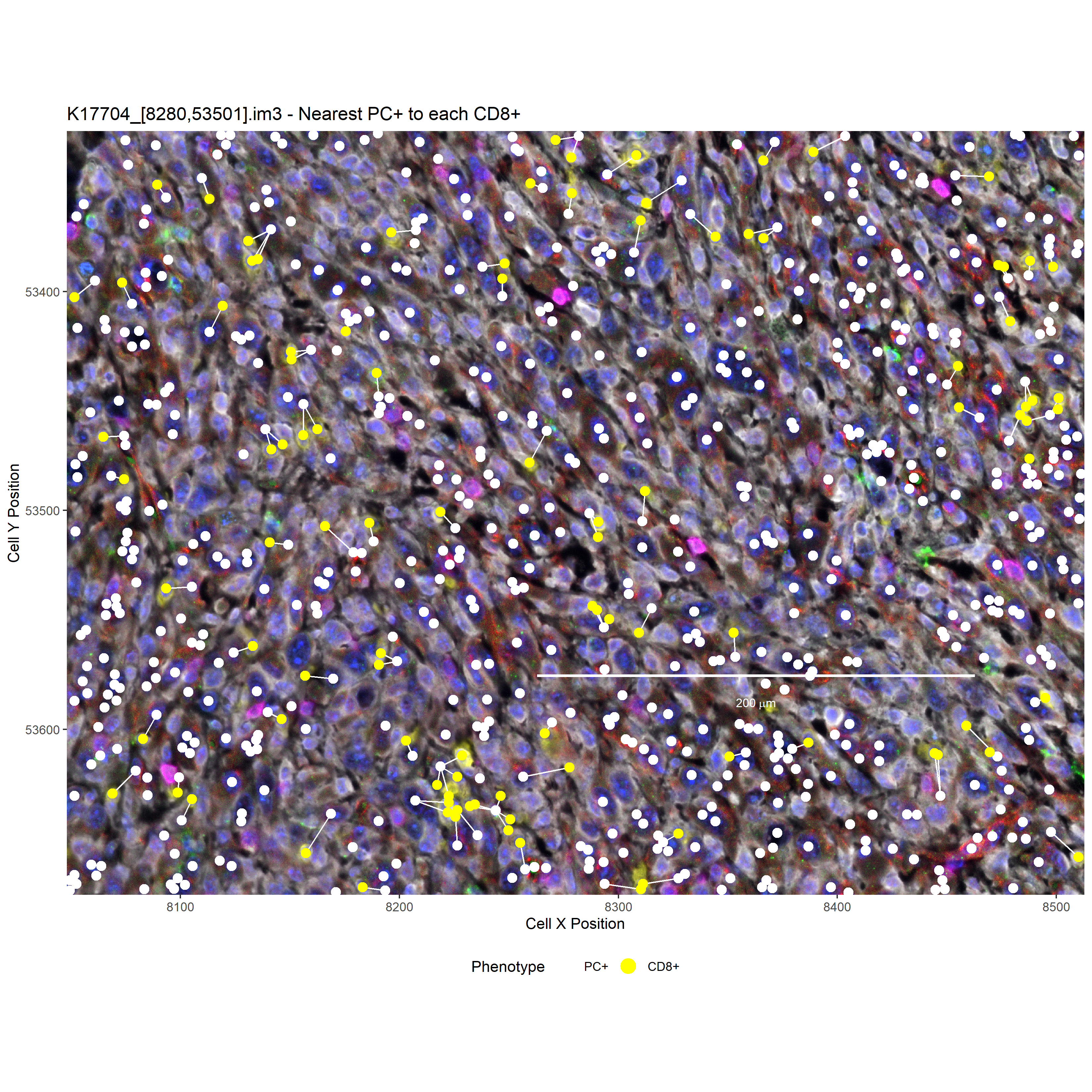

Supplement: Supplementary file 10 — Source data Fig. 8 [file 44321_2024_187_MOESM10_ESM.zip › Figure 8/8A/aPD1_Ctrl_K17704_[8280,53501]_PC+_near_CD8+.png]

$\alpha$ PD1 (Ctrl.)

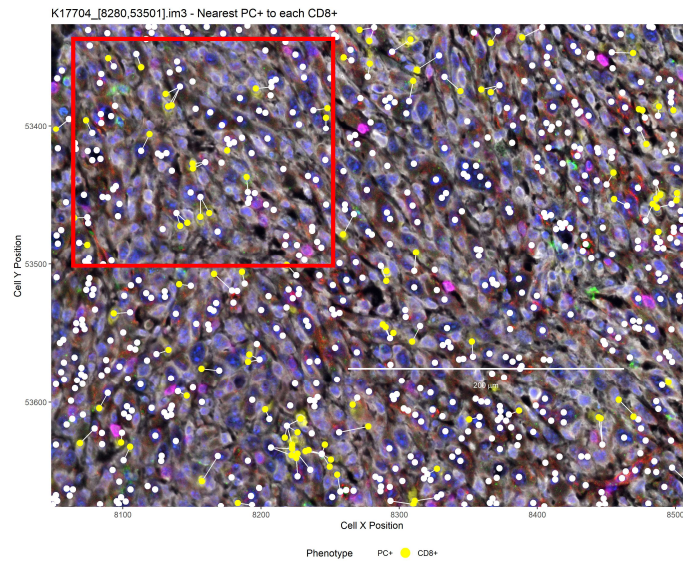

+  $\alpha$ PD1  
Ad5/11 +

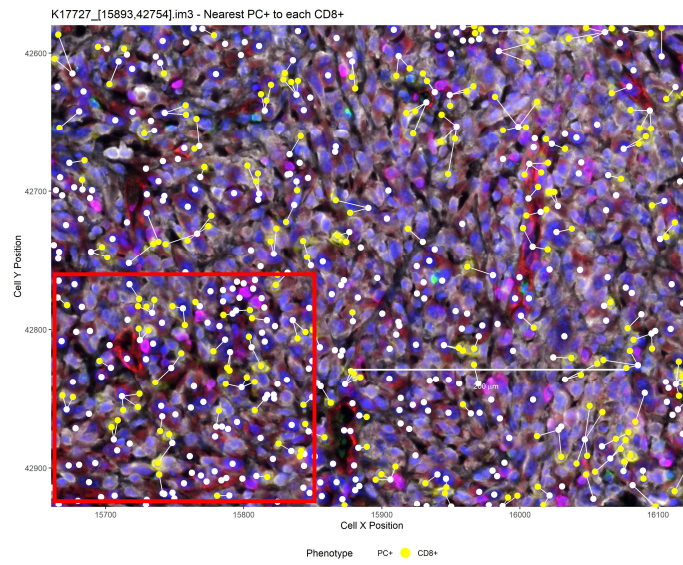

+  $\alpha$ PD1  
Ad5/11- $\alpha$ CD3<sub>TAT</sub>-Trimer

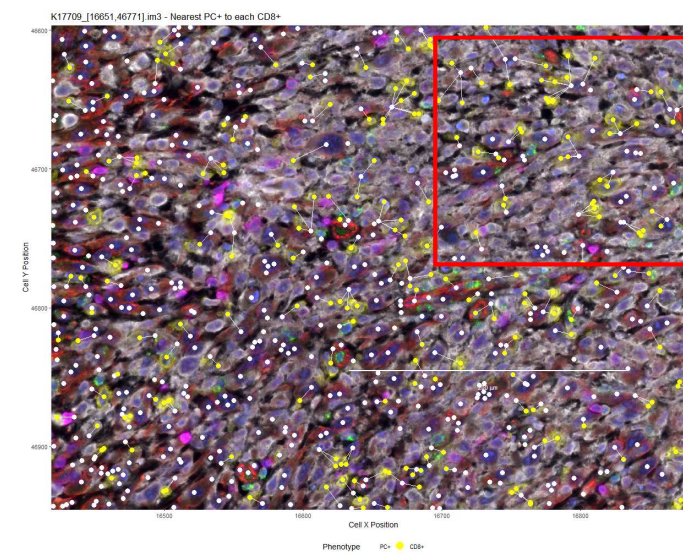

Supplement: Supplementary file 10 — Source data Fig. 8 [file 44321_2024_187_MOESM10_ESM.zip › Figure 8/8A/Fig8A_area of magnification.pdf]

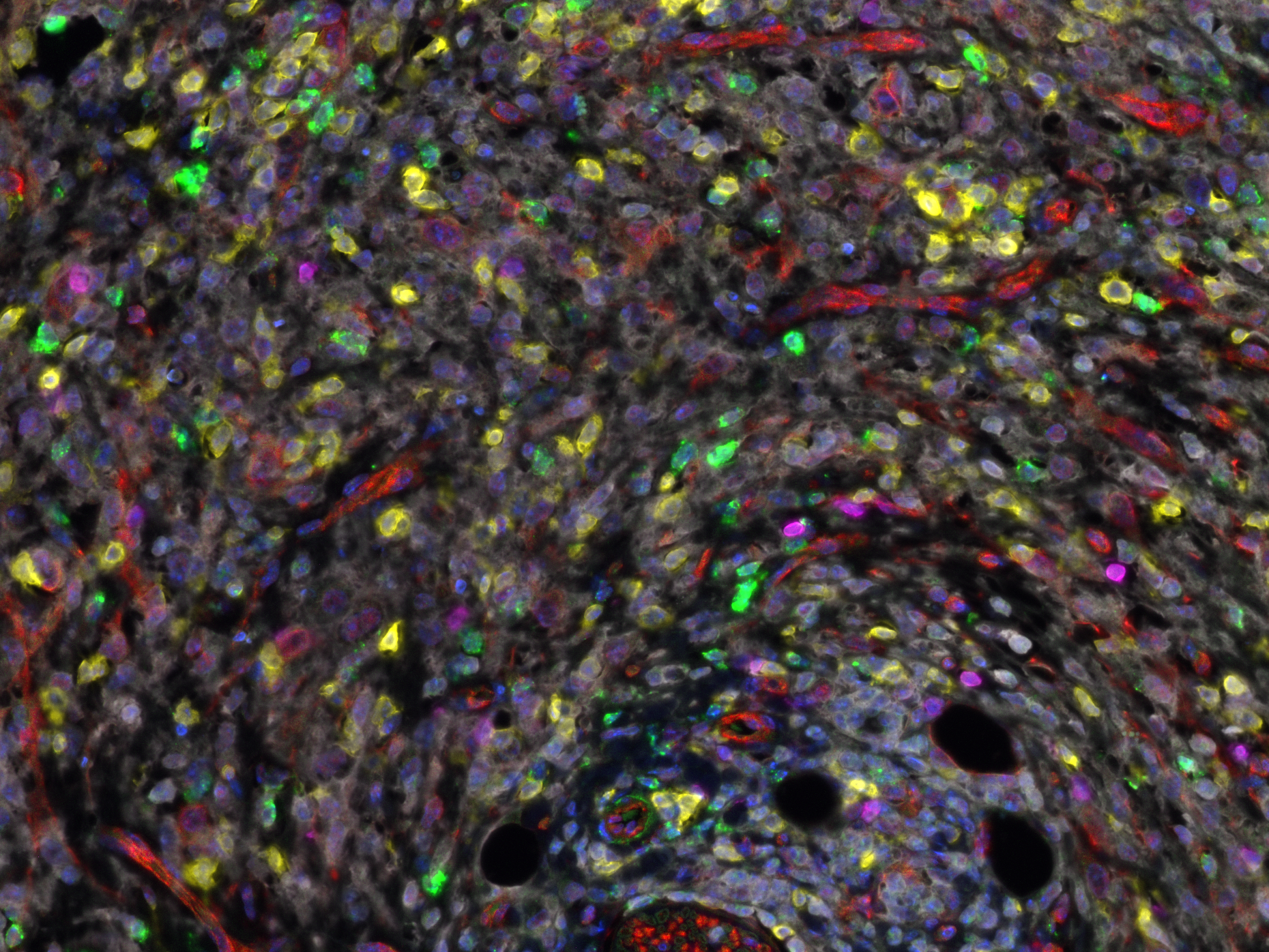

Supplement: Supplementary file 10 — Source data Fig. 8 [file 44321_2024_187_MOESM10_ESM.zip › Figure 8/8E/aPD1+Ad5-11-Trimer_K17711_[10954,55278]_composite_image.tif]

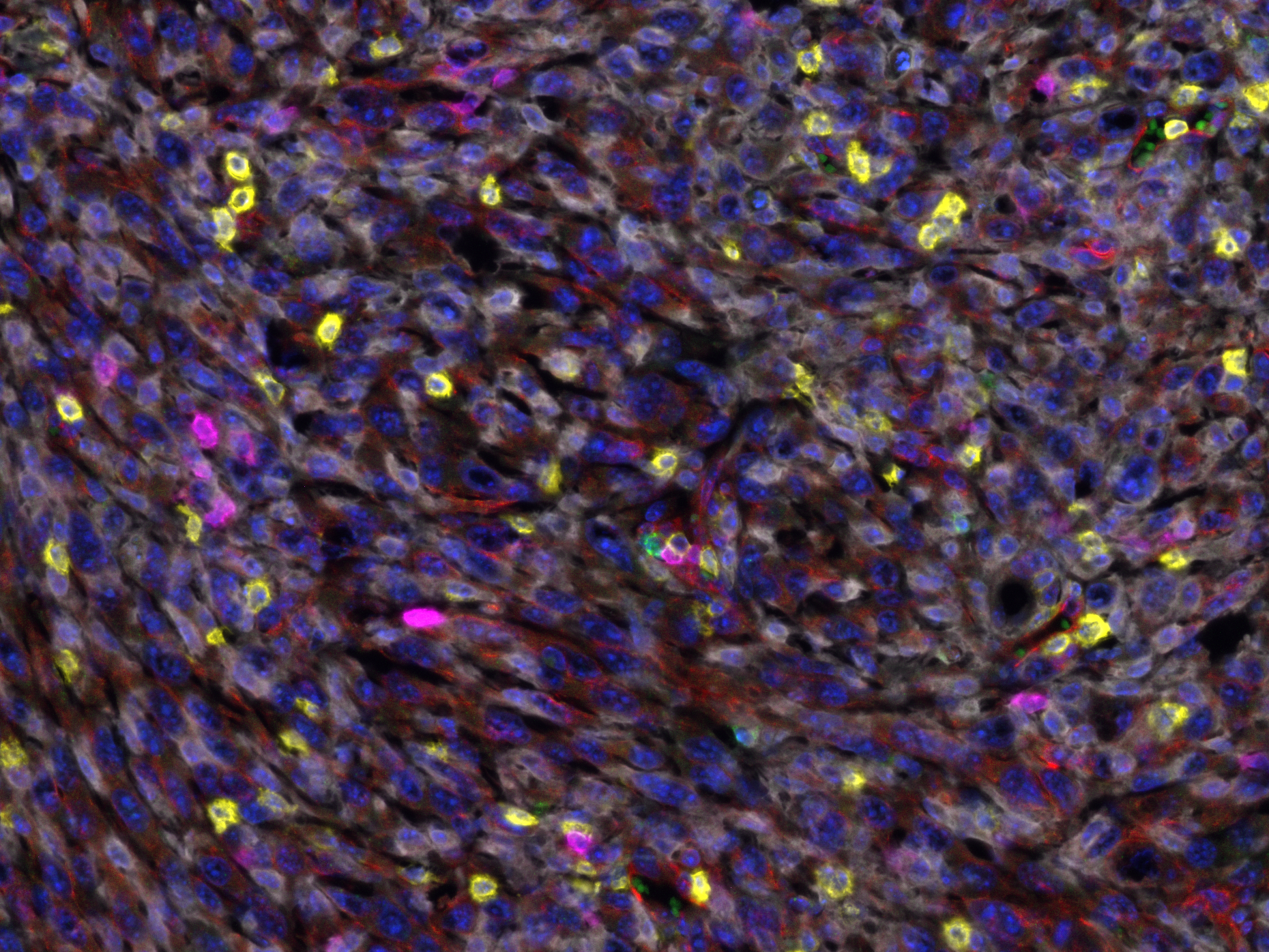

Supplement: Supplementary file 10 — Source data Fig. 8 [file 44321_2024_187_MOESM10_ESM.zip › Figure 8/8E/aPD1+Ad5-11_K17751_[6683,51735]_composite_image.tif]
