# Supplementary material for: Oncolytic viruses expressing MATEs facilitate target-independent T-cell activation in tumors
Source: EMBO Mol Med. 2025 Jan 9;17(2):265–300. doi: 10.1038/s44321-024-00187-y (PMC11821991; doi:10.1038/s44321-024-00187-y)
Supplement: Supplementary file 11 — Expanded View Figures [file 44321_2024_187_MOESM11_ESM.pdf]

Expanded View Figures

Figure EV1. Membrane-associated T-cell engager (MATE) proteins activate different murine CD8^{+} T cells subsets and confer cytotoxicity against tumor cells in vitro. ▶

(**A**) SDS-PAGE and Western-blot analysis of Ni-NTA-purified MATEs using an α-myc antibody is shown in the left panel, a non-denaturing Blue-native PAGE and subsequent western blot analysis is shown on the right hand side. Standards have been stained with Coomassie. (**B**) Murine splenocytes were incubated with 500 ng of purified MATEs and binding to CD8^{+} T-cell subsets, as defined by CD44 and CD62L markers, was analyzed by flow cytometry. (**C**) Murine splenocytes were incubated with increasing amounts of purified MATEs and T-cell activation was measured (number of replicates $n = 3$; mean ± SD; p values by one-way ANOVA with Tukey's post hoc analysis). (**D**) The polarization of CD4^{+} T cells to regulatory T cells (Tregs) in the course of MATE-dependent activation was investigated. The amount of Tregs was analyzed by the intranuclear staining of FoxP3 in CD4^{+} CD25^{+} T cells after 48 h of incubation with 1 µg of purified MATEs. (number of replicates $n = 3$; mean ± SD; p values by one-way ANOVA with Tukey's post hoc analysis). (**E**) Expression analysis of transgenic MC38 cells stably expressing a transmembrane T-cell engager (αCD3$_{TM}$) using flow cytometry. (**F**) Western blot analysis of supernatants obtained from MC38 cells stably expressing MATEs. (**G**) T-cell activation of murine CD4^{+} and CD8^{+} T cells 24 h after coculture with transmembrane T-cell engager-expressing, MATE-expressing or non-expressing MC38 tumor cells (1:10 target:splenocyte) (number of replicates $n = 3$; mean ± SD; p values by one-way ANOVA with Tukey's post hoc analysis). (**H**) Proliferation analysis of CD4^{+} and CD8^{+} T cells after 48 h coculture (1:10 target:splenocyte) with transgenic MC38 cells expressing either MATEs or the transmembrane T-cell engager (number of replicates $n = 3$; mean ± SD; p values by one-way ANOVA with Tukey's post hoc analysis).

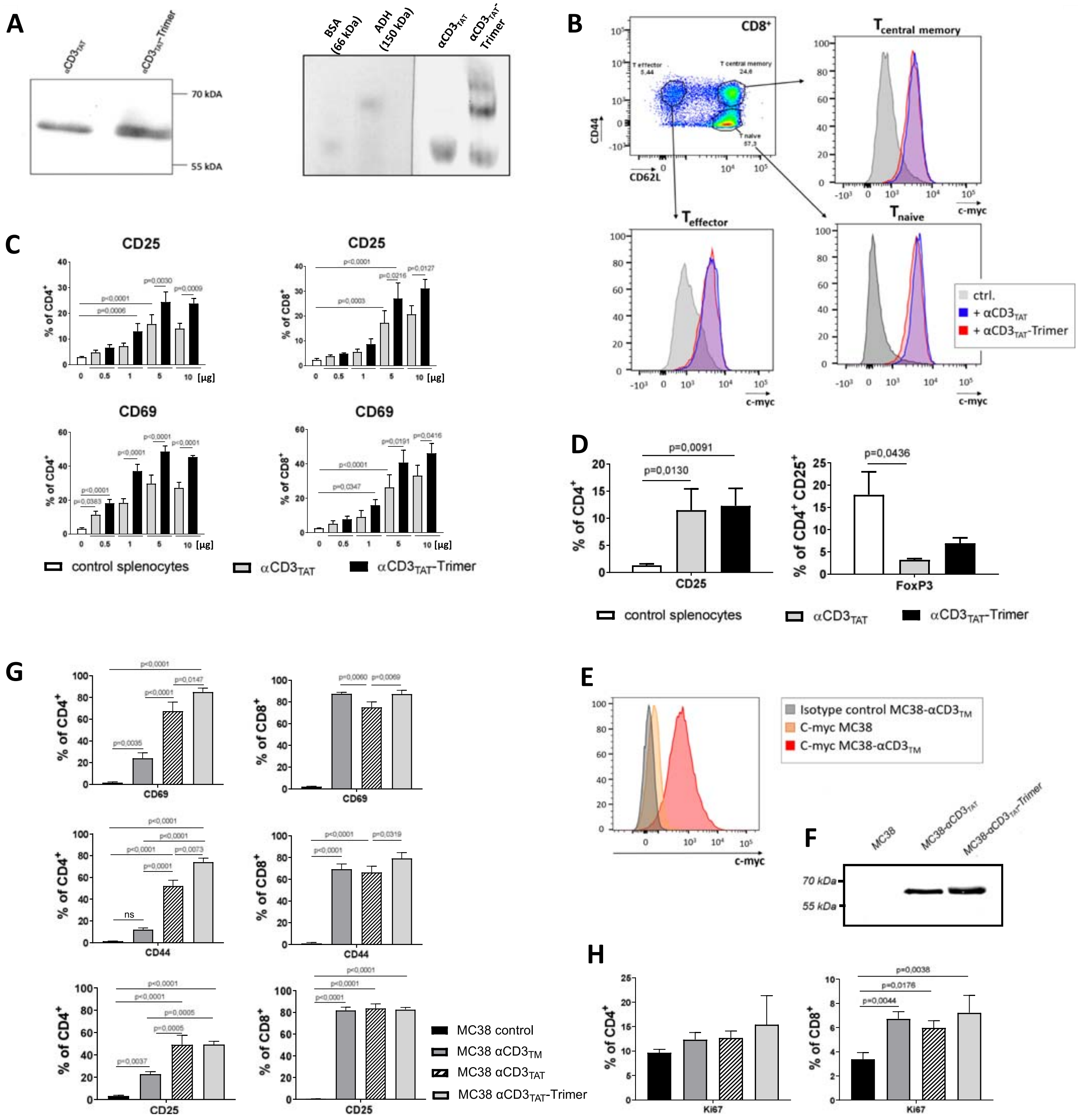
A
αCD3TAT
αCD3TAT-Trimer
70 kDA
55 kDA
BSA (66 kDa)
ADH (150 kDa)
αCD3TAT
αCD3TAT-Trimer
B
CD8+
T effector 5,44
T central memory 24,6
T naive 57,3
CD44
CD62L
Tcentral memory
Teffector
Tnaive
c-myc
ctrl.
+ αCD3TAT
+ αCD3TAT-Trimer
C
CD25
CD69
% of CD4+
% of CD8+
[µg]
control splenocytes
αCD3TAT
αCD3TAT-Trimer
D
p=0,0091
p=0,0130
p=0,0436
% of CD4+
% of CD4+ CD25+
CD25
FoxP3
control splenocytes
αCD3TAT
αCD3TAT-Trimer
E
Isotype control MC38-αCD3TM
C-myc MC38
C-myc MC38-αCD3TM
c-myc
F
MC38
MC38-αCD3TAT
MC38-αCD3TAT-Trimer
70 kDa
55 kDa
G
% of CD4+
% of CD8+
CD69
CD44
CD25
ns
MC38 control
MC38 αCD3TM
MC38 αCD3TAT
MC38 αCD3TAT-Trimer
H
% of CD4+
% of CD8+
Ki67
p=0,0038
p=0,0176
p=0,0044

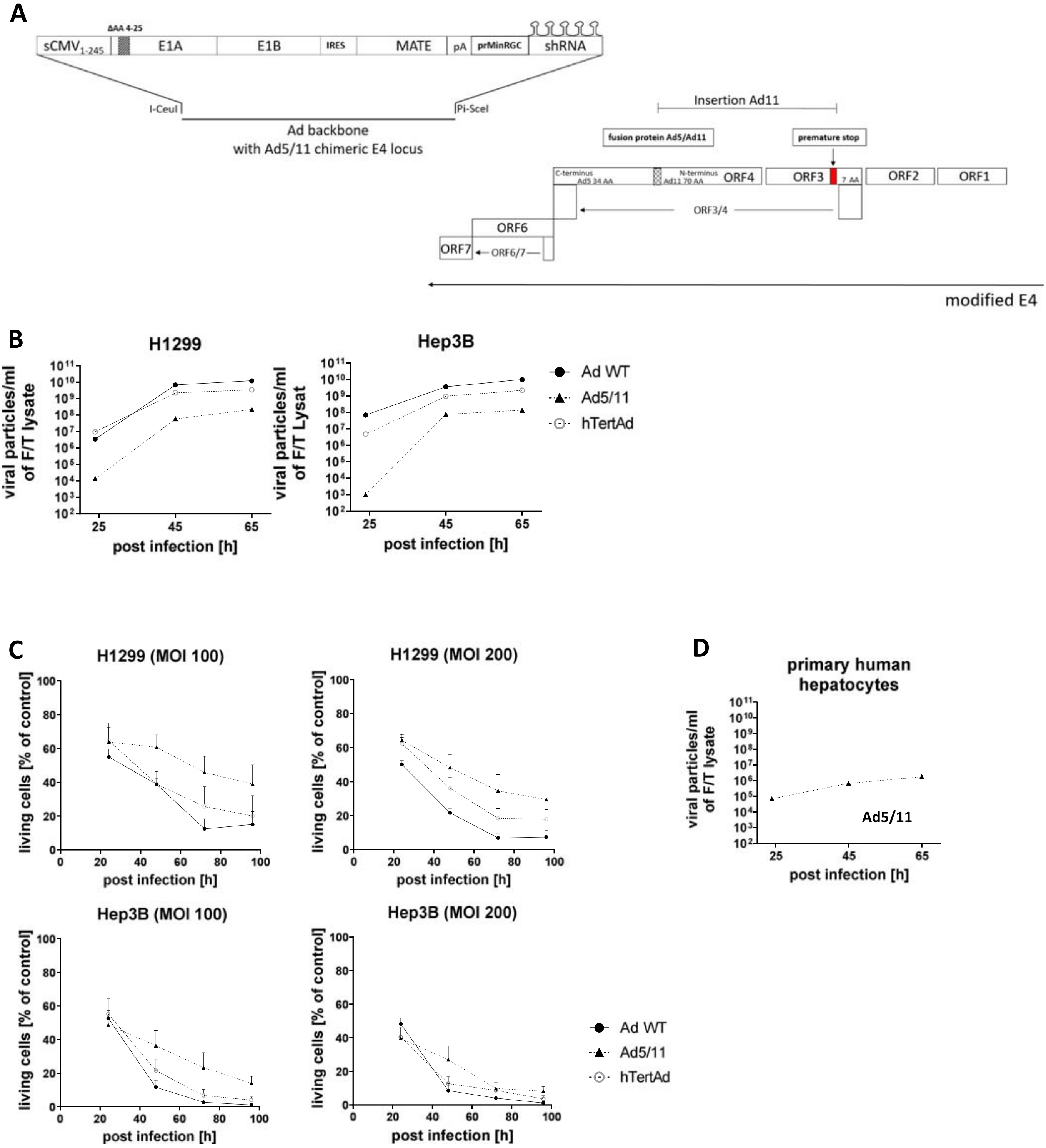

Figure EV2. The oncolytic adenovirus Ad5/11 facilitates replication in and lysis of various human tumor cell lines but is inhibited in normal hepatocytes.

(**A**) Schematic illustration of MATE-expressing Ad5/11 highlighting the regulatory E1 region including the locus for MATE expression (left) and the insertion of adenovirus serotype 11 derived sequences in the E4orf3/orf4 locus (right). (**B**) Replication kinetics of Ad5/11 compared with the Ad5-based oncolytic adenovirus hTertAd and adenovirus wild type (Ad WT) following infection with MOI of 2 in human tumor cell lines Hep3B and H1299. Cells were harvested at time points indicated and infectious viral particles were determined by using Rapid Titer Assay. (**C**) Human tumor cell lines Hep3B and H1299 were infected with Ad5/11, the oncolytic adenovirus hTertAd and adenovirus wild type (Ad WT) at the MOI indicated in the panels. 48 h after infection cell lysis was determined by MTT assays (number of replicates $n = 3$; mean ± SD). (**D**) Replication kinetics of Ad5/11 after infection of primary human hepatocytes with an MOI of 2. Infectious viral particles were determined using the Rapid Titer Assay.

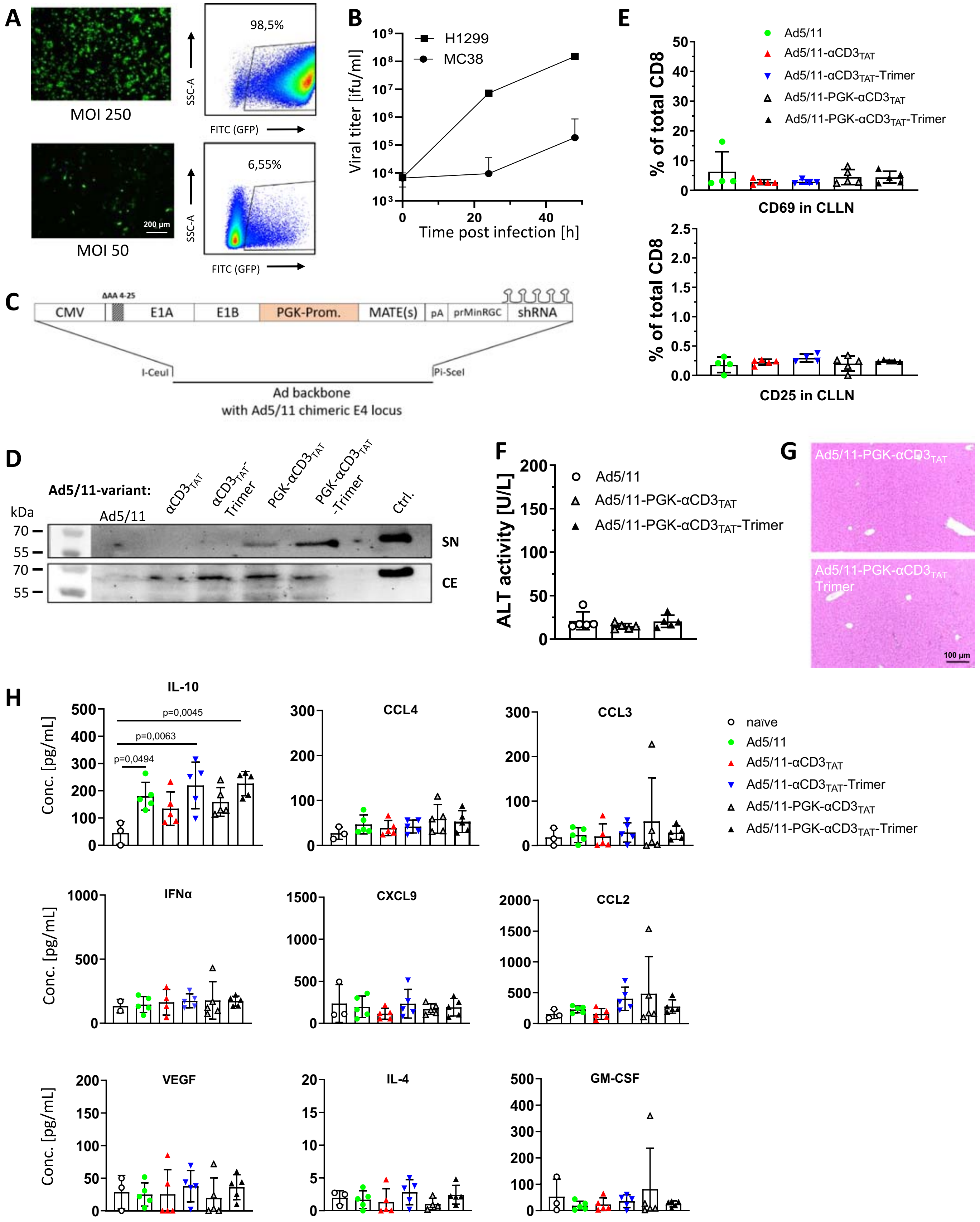
A
MOI 250
98,5%
SSC-A
FITC (GFP)
MOI 50
6,55%
200 µm
B
H1299
MC38
Viral titer [ifu/ml]
Time post infection [h]
C
CMV
ΔAA 4-25
E1A
E1B
PGK-Prom.
MATE(s)
pA
prMinRGC
shRNA
I-CeuI
PI-SceI
Ad backbone
with Ad5/11 chimeric E4 locus
D
Ad5/11-variant:
kDa
Ad5/11
αCD3TAT
αCD3TAT-Trimer
PGK-αCD3TAT
PGK-αCD3TAT-Trimer
Ctrl.
SN
CE
E
Ad5/11
Ad5/11-αCD3TAT
Ad5/11-αCD3TAT-Trimer
Ad5/11-PGK-αCD3TAT
Ad5/11-PGK-αCD3TAT-Trimer
% of total CD8
CD69 in CLLN
CD25 in CLLN
F
ALT activity [U/L]
Ad5/11
Ad5/11-PGK-αCD3TAT
Ad5/11-PGK-αCD3TAT-Trimer
G
Ad5/11-PGK-αCD3TAT
Ad5/11-PGK-αCD3TAT Trimer
100 µm
H
IL-10
p=0,0045
p=0,0063
p=0,0494
CCL4
CCL3
IFNα
CXCL9
CCL2
VEGF
IL-4
GM-CSF
Conc. [pg/mL]
naive
Ad5/11
Ad5/11-αCD3TAT
Ad5/11-αCD3TAT-Trimer
Ad5/11-PGK-αCD3TAT
Ad5/11-PGK-αCD3TAT-Trimer

 © The Author(s)

Figure EV3. Intratumoral virotherapy with enhanced MATE-expression does not lead to systemic toxicity.

(**A**) To assess susceptibility of MC38 cells for MATE-expressing adenoviruses, cells were treated in vitro with a replication-deficient adenoviral serotype 5 based vector expressing a GFP-reporter gene using a MOI as indicated. 48 h following infection, GFP-expression was monitored by fluorescence microscopy and quantified by flow cytometry. Representative images are shown (magnification 100×; scale bar indicates 200 µm). (**B**) Human H1299 cells and murine MC38 cells were infected with MOI 50 of Ad5/11. After 4 h excess virus was removed and generated virus particles were counted by Rapid Titer assay at the time points indicated ($n = 3$, mean ± SD). (**C**) Illustration showing the genetic setup of Ad5/11 variants harboring MATEs in a separate expression cassette under transcriptional control of a phosphoglycerate kinase (PGK) promoter for enhanced MATE expression levels. (**D**) MC38 cells were infected with Ad5/11 or MATE-expressing Ad5/11 variants and supernatants were collected after 48 h. Cell extracts were prepared using RIPA-buffer. MATEs in the supernatant were further enriched by Ni-NTA purification. MATEs were analyzed by western blot analyses using an α-myc antibody. (**E**) S.c. MC38 tumors in C57BL/6 mice were treated with MATE-expressing Ad5/11 variants as shown in the figure and as described in Fig. 4. T-cell activation was determined in the contralateral lymph nodes (CLLN) after 48 h ($n = 4$ mice for Ad5/11 and Ad5/11-αCD3$_{TAT}$-Trimer, $n = 5$ mice for all other groups; mean ± SD) and ALT-activity of the PGK-variants and control virus is shown to complement Fig. 4C (**F**) ($n = 5$ mice; mean ± SD). (**G**) Representative images of H/E stained FFPE liver tissue of mice treated with PGK-variants (magnification 100×; scale bar indicates 100 µm; representative images from each group are shown). (**H**) Additional measurements supplementing main Fig. 4 showing the analysis of serum cytokine levels of treated mice using the Legendplex mouse cytokine release syndrome panel ($n = 3$ mice for naïve controls, $n = 5$ mice for all other groups; mean ± SD; p values by one-way ANOVA with Tukey's post hoc analysis, in case of IFNα two outliers were not included in the panel and no statistical analysis was performed).

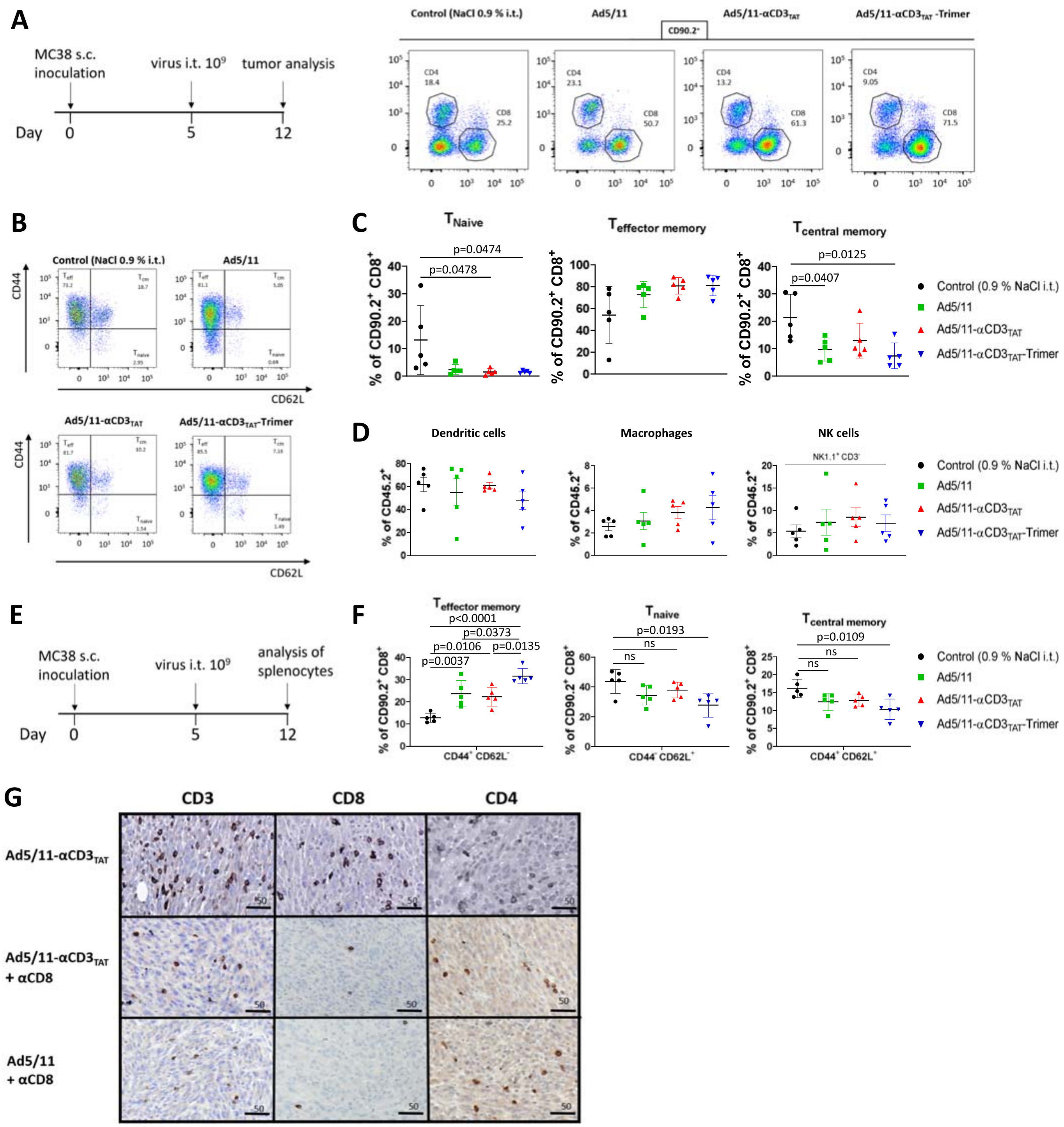

Figure EV4. Intratumoral virotherapy with MATE-expressing Ad5/11 supports intratumoral and systemic immunoactivation.

(**A–D**) S.c. MC38 tumors were established and treated with MATE-expressing Ad5/11 variants as described in the scheme. Mice were sacrificed at day seven after intratumoral virotherapy and leukocyte infiltration in tumor tissue was examined by flow cytometry. (**A**) Representative FACS plots showing $CD4^+$ and $CD8^+$ T-cell populations. (**B**) Representative FACS plots illustrating the subsequent characterization of $CD8^+$ T-cell subsets. (**C**) T-cell subsets were calculated as percentage of $CD90.2^+$ $CD8^+$ lymphocytes ($n = 5$ mice; mean ± SD; p values calculated by one-way ANOVA with Tukey's post hoc analysis). (**D**) Abundance of dendritic cells ($CD11c^+$, $F4/80^-$), macrophages ($F4/80^+$) and NK cells ($NK1.1^+$, $CD3^-$) within the $CD45.2^+$ leukocyte population ($n = 5$ mice; mean ± SD). After tumor treatment as illustrated in (**E**), splenocytes of treated mice were subjected to immune analyses. (**F**) Shows the characterization of $CD8^+$ T-cell subsets within the $CD90.2^+$ $CD8^+$ lymphocyte population ($n = 5$ mice; mean ± SD; p values by one-way ANOVA with Tukey's post hoc analysis). (**G**) MC38 tumor-bearing mice were treated as described in Fig. 6A. Immunohistochemical staining of T-cell populations within tumor tissue following systemic application of a $CD8^+$ depleting antibody was performed to confirm the success of CD8 T cell depletion (Magnification 200×, bars indicate 50 μm, Images showing CD3 and CD8 staining in the Ad5/11-αCD3$_{TAT}$ group have been reused from Fig. 5C).

 © The Author(s)

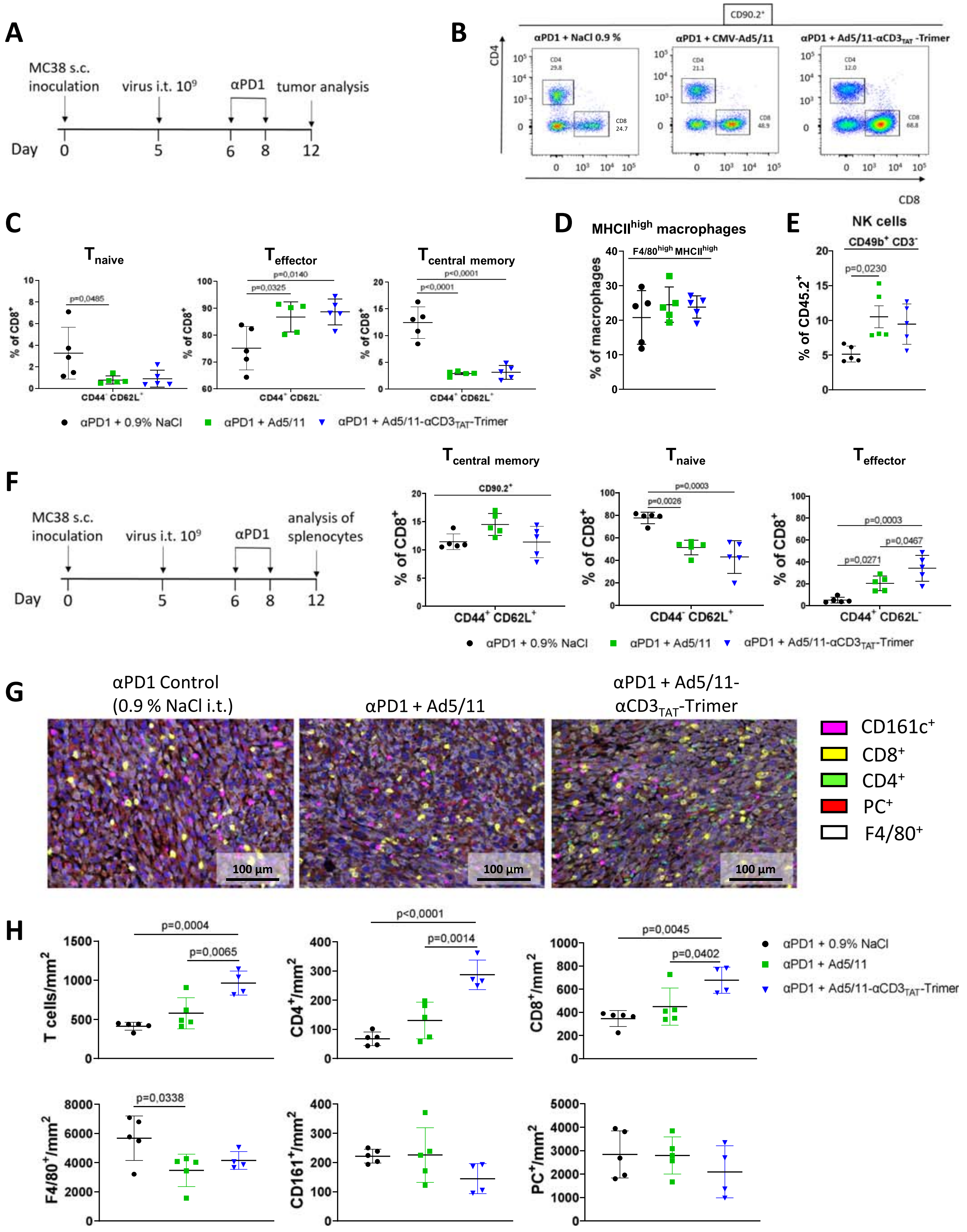
A
MC38 s.c. inoculation
virus i.t. 10^9
αPD1
tumor analysis
Day 0 5 6 8 12
B
CD90.2+
αPD1 + NaCl 0.9 %
αPD1 + CMV-Ad5/11
αPD1 + Ad5/11-αCD3TAT-Trimer
CD4
CD8
C
Tnaive
Teffector
Tcentral memory
% of CD8+
p=0,0485
p=0,0140
p=0,0325
p<0,0001
p<0,0001
CD44- CD62L+
CD44+ CD62L-
CD44+ CD62L+
D
MHCIIhigh macrophages
F4/80high MHCIIhigh
% of macrophages
E
NK cells
CD49b+ CD3-
p=0,0230
% of CD45.2+
αPD1 + 0.9% NaCl
αPD1 + Ad5/11
αPD1 + Ad5/11-αCD3TAT-Trimer
F
MC38 s.c. inoculation
virus i.t. 10^9
αPD1
analysis of splenocytes
Day 0 5 6 8 12
Tcentral memory
Tnaive
Teffector
CD90.2+
p=0,0003
p=0,0026
p=0,0003
p=0,0467
p=0,0271
% of CD8+
CD44+ CD62L+
CD44- CD62L+
CD44+ CD62L-
αPD1 + 0.9% NaCl
αPD1 + Ad5/11
αPD1 + Ad5/11-αCD3TAT-Trimer
G
αPD1 Control (0.9 % NaCl i.t.)
αPD1 + Ad5/11
αPD1 + Ad5/11-αCD3TAT-Trimer
100 µm
CD161c+
CD8+
CD4+
PC+
F4/80+
H
p=0,0004
p=0,0065
T cells/mm2
p<0,0001
p=0,0014
CD4+/mm2
p=0,0045
p=0,0402
CD8+/mm2
αPD1 + 0.9% NaCl
αPD1 + Ad5/11
αPD1 + Ad5/11-αCD3TAT-Trimer
p=0,0338
F4/80+/mm2
CD161+/mm2
PC+/mm2

Figure EV5. Combination of MATE-expressing virotherapy and PD1 checkpoint blockade modulates the tumor microenvironment and promotes intratumoral T-cell infiltration.

(**A**) S.c. MC38 tumors were established and treated with virotherapy followed by two i.p. applications of αPD1 antibodies as illustrated. Seven days after virotherapy, tumor tissue was investigated via flow cytometry to determine $CD4^+$ and $CD8^+$ T-cell infiltration (**B**), to characterize $CD8^+$ T-cell subsets (**C**) and to determine the frequency of further myeloid immune cells (**D**, **E**). (**C**) T-cell subsets are given as frequency within the $CD8^+$ T-cell population ($n = 5$ mice; mean ± SD; p values by one-way ANOVA with Tukey's post hoc analysis). (**D**) shows the amount of the M1-subtype ($MHCII^{high}$) within the macrophage population ($CD45.2^+$, $F4/80^+$), Frequency of NK cells ($CD49b^+$, $CD3^-$) was calculated within the $CD45.2^+$ leukocyte population (**E**) ($n = 5$ mice; mean ± SD; p values by one-way ANOVA with Tukey's post hoc analysis). (**F**) Spleens of treated mice were prepared to analyze $CD8^+$ T-cell subsets ($n = 5$ mice; mean ± SD; p values by one-way ANOVA with Tukey's post hoc analysis). (**G**) As described for main Fig. 8, MC38 tumor tissue was obtained from tumor-bearing mice 7 days after intratumoral virotherapy followed by two applications of αPD1 antibodies. Spatial immune cell phenotyping was performed by multiplex immunohistochemical staining of $CD8^+$ T cells (yellow), $CD4^+$ T cells (green), $CD161c^+$ NK cells (magenta), $F4/80^+$ macrophages (white), pan-$Cadherin^+$ (PC^+) tumor cells (red), and DAPI staining for cell nuclei (blue). Additional representative composite images from each group are shown (**G**). The densities of the investigated cell types as numbers per area are shown in (**H**) ($n = 4$ mice for Ad5/11-α$CD3_{TAT}$-Trimer + αPD1, all other groups $n = 5$ mice; mean ± SD; p values by one-way ANOVA with Tukey's post hoc analysis).

 © The Author(s)
